# Supplementary material for: Differential gene expression and alternative splicing in insect immune specificity
Source: BMC Genomics. 2014 Nov 27;15(1):1031. doi: 10.1186/1471-2164-15-1031 (PMC4302123; doi:10.1186/1471-2164-15-1031)
Supplement: Supplementary file 2 — Additional file 2: Table S2: The complete output of the DEXSeq analysis is available from http://dx.doi.org/10.6084/m9.figshare.1053092. (ZIP 44 KB) [file 12864_2014_6844_MOESM2_ESM.zip › 1778661205138218_add2.html]

/Users/eamonnmallon/Documents/Papers/alternative\_splicing/may.html


# DEXSeq differential exon usage test

## Experimental design

|  |  |  |  |  |
| --- | --- | --- | --- | --- |
| sample | colony | strain | libType | interaction |
| K61 | k | six | single-read | k6 |
| K62 | k | six | single-read | k6 |
| K63 | k | six | single-read | k6 |
| K81 | k | eight | single-read | k8 |
| K82 | k | eight | single-read | k8 |
| K83 | k | eight | single-read | k8 |
| Q61 | q | six | single-read | q6 |
| Q62 | q | six | single-read | q6 |
| Q63 | q | six | single-read | q6 |
| Q81 | q | eight | single-read | q8 |
| Q82 | q | eight | single-read | q8 |

### formulaDispersion = full

### formula0 = reduced

### formula1 = full

## testForDEU result table

|  |  |  |  |  |  |
| --- | --- | --- | --- | --- | --- |
| geneID | chr | start | end | total\_exons | exon\_changes |
| XLOC\_000588 | gi|313874739|gb|AELG01006894.1| | 1 | 1001 | 5 | 1 |
| XLOC\_000661 | gi|313875374|gb|AELG01006259.1| | 3323 | 59977 | 21 | 1 |
| XLOC\_000664 | gi|313875375|gb|AELG01006258.1| | 7896 | 35393 | 17 | 1 |
| XLOC\_000678 | gi|313875377|gb|AELG01006256.1| | 33509 | 37390 | 8 | 2 |
| XLOC\_000682 | gi|313875378|gb|AELG01006255.1| | 13180 | 28716 | 10 | 3 |
| XLOC\_000689 XLOC\_000691 | gi|313875379|gb|AELG01006254.1| | 12279 | 34190 | 34 | 12 |
| XLOC\_000707 XLOC\_000703 | gi|313875380|gb|AELG01006253.1| | 44710 | 47666 | 15 | 1 |
| XLOC\_000715 | gi|313875386|gb|AELG01006247.1| | 2357 | 8742 | 2 | 2 |
| XLOC\_000716 | gi|313875392|gb|AELG01006241.1| | 46 | 5628 | 14 | 2 |
| XLOC\_000720 | gi|313875393|gb|AELG01006240.1| | 42260 | 45024 | 6 | 1 |
| XLOC\_000721 | gi|313875393|gb|AELG01006240.1| | 166540 | 172818 | 11 | 1 |
| XLOC\_000724 | gi|313875393|gb|AELG01006240.1| | 9417 | 41591 | 9 | 2 |
| XLOC\_000726 | gi|313875393|gb|AELG01006240.1| | 51996 | 166014 | 11 | 4 |
| XLOC\_000730 XLOC\_000728 | gi|313875394|gb|AELG01006239.1| | 25594 | 55522 | 22 | 5 |
| XLOC\_000739 | gi|313875395|gb|AELG01006238.1| | 73262 | 75527 | 4 | 1 |
| XLOC\_000760 | gi|313875403|gb|AELG01006230.1| | 10172 | 16160 | 4 | 3 |
| XLOC\_000761 | gi|313875403|gb|AELG01006230.1| | 5394 | 9943 | 8 | 2 |
| XLOC\_000776 | gi|313875404|gb|AELG01006229.1| | 34275 | 45352 | 6 | 2 |
| XLOC\_000777 | gi|313875404|gb|AELG01006229.1| | 51682 | 59913 | 5 | 1 |
| XLOC\_000794 | gi|313875411|gb|AELG01006222.1| | 5887 | 9786 | 6 | 3 |
| XLOC\_000813 | gi|313875416|gb|AELG01006217.1| | 2 | 63750 | 9 | 6 |
| XLOC\_000818 | gi|313875418|gb|AELG01006215.1| | 31362 | 36205 | 13 | 2 |
| XLOC\_000827 | gi|313875418|gb|AELG01006215.1| | 67100 | 73713 | 16 | 1 |
| XLOC\_000830 | gi|313875419|gb|AELG01006214.1| | 32635 | 39923 | 8 | 1 |
| XLOC\_000833 XLOC\_000834 | gi|313875419|gb|AELG01006214.1| | 61070 | 83634 | 37 | 6 |
| XLOC\_000846 | gi|313875426|gb|AELG01006207.1| | 37914 | 43583 | 5 | 2 |
| XLOC\_000870 | gi|313875432|gb|AELG01006201.1| | 404 | 11078 | 11 | 2 |
| XLOC\_000875 XLOC\_000874 | gi|313875433|gb|AELG01006200.1| | 10276 | 19341 | 15 | 3 |
| XLOC\_000878 | gi|313875435|gb|AELG01006198.1| | 11452 | 214533 | 15 | 5 |
| XLOC\_000879 | gi|313875436|gb|AELG01006197.1| | 4931 | 8439 | 5 | 2 |
| XLOC\_000887 XLOC\_000893 | gi|313875441|gb|AELG01006192.1| | 34445 | 63392 | 42 | 2 |
| XLOC\_000891 XLOC\_000885 XLOC\_000886 XLOC\_000892 | gi|313875441|gb|AELG01006192.1| | 17244 | 33534 | 30 | 1 |
| XLOC\_000896 | gi|313875441|gb|AELG01006192.1| | 63641 | 69053 | 6 | 1 |
| XLOC\_000899 | gi|313875441|gb|AELG01006192.1| | 79897 | 83866 | 7 | 1 |
| XLOC\_000901 | gi|313875441|gb|AELG01006192.1| | 116516 | 167510 | 15 | 5 |
| XLOC\_000902 | gi|313875441|gb|AELG01006192.1| | 181204 | 206859 | 14 | 6 |
| XLOC\_000903 XLOC\_000904 XLOC\_000890 | gi|313875441|gb|AELG01006192.1| | 217644 | 306496 | 55 | 1 |
| XLOC\_000922 | gi|313875444|gb|AELG01006189.1| | 51770 | 57861 | 16 | 1 |
| XLOC\_000923 | gi|313875444|gb|AELG01006189.1| | 58960 | 69364 | 13 | 2 |
| XLOC\_000933 | gi|313875446|gb|AELG01006187.1| | 30831 | 35001 | 10 | 1 |
| XLOC\_000935 | gi|313875446|gb|AELG01006187.1| | 48997 | 57448 | 8 | 2 |
| XLOC\_000939 | gi|313875448|gb|AELG01006185.1| | 6001 | 22925 | 13 | 4 |
| XLOC\_000943 | gi|313875452|gb|AELG01006181.1| | 59 | 4110 | 12 | 1 |
| XLOC\_000960 | gi|313875458|gb|AELG01006175.1| | 1188 | 6246 | 6 | 1 |
| XLOC\_000962 | gi|313875458|gb|AELG01006175.1| | 14880 | 44539 | 18 | 1 |
| XLOC\_001013 XLOC\_001014 | gi|313875474|gb|AELG01006159.1| | 3758 | 23695 | 35 | 2 |
| XLOC\_001018 | gi|313875475|gb|AELG01006158.1| | 129 | 4234 | 7 | 1 |
| XLOC\_001019 XLOC\_001017 | gi|313875475|gb|AELG01006158.1| | 13015 | 18141 | 11 | 1 |
| XLOC\_001026 | gi|313875476|gb|AELG01006157.1| | 94707 | 99522 | 11 | 1 |
| XLOC\_001027 | gi|313875476|gb|AELG01006157.1| | 1 | 4161 | 8 | 1 |
| XLOC\_001035 XLOC\_001025 | gi|313875476|gb|AELG01006157.1| | 88446 | 93032 | 15 | 3 |
| XLOC\_001036 | gi|313875476|gb|AELG01006157.1| | 102010 | 107648 | 3 | 1 |
| XLOC\_001046 | gi|313875477|gb|AELG01006156.1| | 35858 | 43462 | 7 | 1 |
| XLOC\_001065 | gi|313875483|gb|AELG01006150.1| | 1739 | 7154 | 16 | 2 |
| XLOC\_001075 | gi|313875484|gb|AELG01006149.1| | 11773 | 24443 | 15 | 2 |
| XLOC\_001077 | gi|313875484|gb|AELG01006149.1| | 38563 | 46506 | 14 | 2 |
| XLOC\_001084 XLOC\_001086 | gi|313875486|gb|AELG01006147.1| | 65221 | 115329 | 33 | 5 |
| XLOC\_001125 | gi|313875555|gb|AELG01006078.1| | 1109 | 5954 | 6 | 1 |
| XLOC\_001150 | gi|313875574|gb|AELG01006059.1| | 101198 | 108503 | 20 | 1 |
| XLOC\_001154 | gi|313875574|gb|AELG01006059.1| | 94209 | 100193 | 15 | 1 |
| XLOC\_001165 | gi|313875575|gb|AELG01006058.1| | 48268 | 105156 | 6 | 2 |
| XLOC\_001181 | gi|313875590|gb|AELG01006043.1| | 5468 | 15901 | 3 | 2 |
| XLOC\_001234 XLOC\_001231 | gi|313875640|gb|AELG01005993.1| | 14107 | 21868 | 17 | 1 |
| XLOC\_001237 XLOC\_001242 | gi|313875640|gb|AELG01005993.1| | 48277 | 59031 | 8 | 2 |
| XLOC\_001291 | gi|313875733|gb|AELG01005901.1| | 19265 | 29038 | 17 | 2 |
| XLOC\_001295 XLOC\_001292 | gi|313875733|gb|AELG01005901.1| | 37557 | 59130 | 41 | 9 |
| XLOC\_001300 | gi|313875734|gb|AELG01005900.1| | 2 | 39121 | 12 | 5 |
| XLOC\_001354 | gi|313875749|gb|AELG01005885.1| | 130883 | 161974 | 43 | 4 |
| XLOC\_001358 | gi|313875749|gb|AELG01005885.1| | 186487 | 277788 | 34 | 10 |
| XLOC\_001367 | gi|313875753|gb|AELG01005881.1| | 18352 | 25015 | 10 | 3 |
| XLOC\_001370 | gi|313875754|gb|AELG01005880.1| | 5075 | 15036 | 7 | 3 |
| XLOC\_001371 | gi|313875754|gb|AELG01005880.1| | 17283 | 22741 | 7 | 2 |
| XLOC\_001381 | gi|313875756|gb|AELG01005878.1| | 23514 | 29207 | 3 | 1 |
| XLOC\_001398 | gi|313875765|gb|AELG01005869.1| | 53293 | 60096 | 9 | 2 |
| XLOC\_001416 XLOC\_001414 | gi|313875780|gb|AELG01005854.1| | 44765 | 54839 | 27 | 2 |
| XLOC\_001433 | gi|313875782|gb|AELG01005852.1| | 46963 | 51262 | 7 | 1 |
| XLOC\_001438 XLOC\_001448 | gi|313875782|gb|AELG01005852.1| | 80030 | 94082 | 10 | 3 |
| XLOC\_001443 | gi|313875782|gb|AELG01005852.1| | 12262 | 26243 | 14 | 2 |
| XLOC\_001444 XLOC\_001432 | gi|313875782|gb|AELG01005852.1| | 24587 | 45615 | 24 | 1 |
| XLOC\_001447 XLOC\_001437 | gi|313875782|gb|AELG01005852.1| | 73429 | 79442 | 27 | 1 |
| XLOC\_001475 XLOC\_001476 | gi|313875790|gb|AELG01005844.1| | 108394 | 116868 | 15 | 2 |
| XLOC\_001488 | gi|313875793|gb|AELG01005841.1| | 31314 | 46306 | 12 | 5 |
| XLOC\_001496 XLOC\_001506 | gi|313875794|gb|AELG01005840.1| | 68937 | 104817 | 29 | 3 |
| XLOC\_001501 | gi|313875794|gb|AELG01005840.1| | 20724 | 34951 | 14 | 2 |
| XLOC\_001521 | gi|313875795|gb|AELG01005839.1| | 82311 | 156470 | 13 | 1 |
| XLOC\_001530 XLOC\_001532 | gi|313875798|gb|AELG01005836.1| | 40425 | 52177 | 53 | 1 |
| XLOC\_001531 | gi|313875798|gb|AELG01005836.1| | 20035 | 31709 | 9 | 2 |
| XLOC\_001536 | gi|313875799|gb|AELG01005835.1| | 201638 | 220689 | 7 | 3 |
| XLOC\_001548 | gi|313875801|gb|AELG01005833.1| | 1332 | 21753 | 10 | 1 |
| XLOC\_001556 XLOC\_001558 | gi|313875804|gb|AELG01005830.1| | 4009 | 18710 | 15 | 1 |
| XLOC\_001565 | gi|313875807|gb|AELG01005827.1| | 6556 | 40438 | 10 | 2 |
| XLOC\_001593 XLOC\_001591 | gi|313875829|gb|AELG01005806.1| | 16871 | 33563 | 36 | 12 |
| XLOC\_001603 | gi|313875849|gb|AELG01005786.1| | 22371 | 30382 | 9 | 8 |
| XLOC\_001616 | gi|313875864|gb|AELG01005771.1| | 25853 | 63274 | 11 | 2 |
| XLOC\_001618 | gi|313875864|gb|AELG01005771.1| | 18267 | 25648 | 24 | 2 |
| XLOC\_001622 | gi|313875865|gb|AELG01005770.1| | 51316 | 59056 | 15 | 1 |
| XLOC\_001654 | gi|313875887|gb|AELG01005749.1| | 42726 | 48888 | 13 | 1 |
| XLOC\_001663 XLOC\_001664 XLOC\_001670 | gi|313875889|gb|AELG01005747.1| | 33703 | 62509 | 37 | 7 |
| XLOC\_001669 | gi|313875889|gb|AELG01005747.1| | 8006 | 31007 | 26 | 12 |
| XLOC\_001694 XLOC\_001686 | gi|313875890|gb|AELG01005746.1| | 52881 | 74880 | 40 | 5 |
| XLOC\_001696 | gi|313875890|gb|AELG01005746.1| | 114994 | 120693 | 8 | 2 |
| XLOC\_001715 | gi|313875893|gb|AELG01005743.1| | 3424 | 5084 | 7 | 1 |
| XLOC\_001718 | gi|313875894|gb|AELG01005742.1| | 2482 | 9941 | 6 | 1 |
| XLOC\_001723 | gi|313875897|gb|AELG01005739.1| | 10585 | 15141 | 14 | 1 |
| XLOC\_001734 | gi|313875899|gb|AELG01005737.1| | 366 | 9051 | 14 | 1 |
| XLOC\_001738 XLOC\_001736 | gi|313875900|gb|AELG01005736.1| | 9093 | 15462 | 29 | 1 |
| XLOC\_001745 | gi|313875904|gb|AELG01005732.1| | 5809 | 22416 | 9 | 1 |
| XLOC\_001752 XLOC\_001748 | gi|313875904|gb|AELG01005732.1| | 49053 | 71266 | 13 | 3 |
| XLOC\_001766 XLOC\_001778 | gi|313875908|gb|AELG01005728.1| | 67337 | 82914 | 23 | 1 |
| XLOC\_001767 XLOC\_001779 | gi|313875908|gb|AELG01005728.1| | 83249 | 100417 | 56 | 6 |
| XLOC\_001773 XLOC\_001786 | gi|313875908|gb|AELG01005728.1| | 152245 | 171834 | 28 | 5 |
| XLOC\_001775 XLOC\_001762 | gi|313875908|gb|AELG01005728.1| | 3667 | 50823 | 43 | 5 |
| XLOC\_001776 | gi|313875908|gb|AELG01005728.1| | 51002 | 55874 | 14 | 4 |
| XLOC\_001782 XLOC\_001770 | gi|313875908|gb|AELG01005728.1| | 122181 | 132360 | 25 | 3 |
| XLOC\_001793 | gi|313875909|gb|AELG01005727.1| | 28019 | 32364 | 18 | 2 |
| XLOC\_001811 | gi|313875916|gb|AELG01005720.1| | 678 | 16145 | 6 | 1 |
| XLOC\_001812 | gi|313875916|gb|AELG01005720.1| | 17105 | 46613 | 7 | 2 |
| XLOC\_001819 | gi|313875917|gb|AELG01005719.1| | 196 | 4319 | 3 | 1 |
| XLOC\_001823 XLOC\_001818 XLOC\_001817 XLOC\_001824 | gi|313875917|gb|AELG01005719.1| | 36382 | 43651 | 25 | 1 |
| XLOC\_001827 | gi|313875918|gb|AELG01005718.1| | 5607 | 19076 | 31 | 15 |
| XLOC\_001828 | gi|313875919|gb|AELG01005717.1| | 8512 | 12536 | 7 | 1 |
| XLOC\_001829 XLOC\_001832 | gi|313875919|gb|AELG01005717.1| | 12960 | 28860 | 17 | 6 |
| XLOC\_001830 | gi|313875919|gb|AELG01005717.1| | 29156 | 36308 | 17 | 2 |
| XLOC\_001833 | gi|313875919|gb|AELG01005717.1| | 46025 | 54156 | 18 | 1 |
| XLOC\_001837 | gi|313875920|gb|AELG01005716.1| | 2655 | 9446 | 9 | 5 |
| XLOC\_001838 | gi|313875920|gb|AELG01005716.1| | 35481 | 40778 | 9 | 1 |
| XLOC\_001851 XLOC\_001846 | gi|313875921|gb|AELG01005715.1| | 36899 | 42171 | 9 | 2 |
| XLOC\_001858 | gi|313875922|gb|AELG01005714.1| | 3225 | 7600 | 11 | 3 |
| XLOC\_001861 XLOC\_001872 | gi|313875923|gb|AELG01005713.1| | 10906 | 20867 | 16 | 3 |
| XLOC\_001865 | gi|313875923|gb|AELG01005713.1| | 56250 | 63731 | 11 | 1 |
| XLOC\_001871 | gi|313875923|gb|AELG01005713.1| | 4516 | 10497 | 10 | 2 |
| XLOC\_001877 | gi|313875923|gb|AELG01005713.1| | 44662 | 49912 | 11 | 1 |
| XLOC\_001878 | gi|313875923|gb|AELG01005713.1| | 78446 | 95420 | 29 | 14 |
| XLOC\_001907 | gi|313875937|gb|AELG01005699.1| | 9257 | 16610 | 8 | 1 |
| XLOC\_001915 | gi|313875938|gb|AELG01005698.1| | 9064 | 12992 | 7 | 1 |
| XLOC\_001924 XLOC\_001925 XLOC\_001918 | gi|313875938|gb|AELG01005698.1| | 31344 | 57419 | 47 | 2 |
| XLOC\_001940 | gi|313875941|gb|AELG01005695.1| | 135 | 12990 | 5 | 3 |
| XLOC\_001951 | gi|313875946|gb|AELG01005690.1| | 12317 | 24819 | 11 | 3 |
| XLOC\_001957 | gi|313875950|gb|AELG01005686.1| | 16 | 10046 | 4 | 3 |
| XLOC\_001960 | gi|313875954|gb|AELG01005682.1| | 1681 | 7485 | 9 | 1 |
| XLOC\_001962 | gi|313875954|gb|AELG01005682.1| | 25900 | 31192 | 8 | 1 |
| XLOC\_001963 | gi|313875954|gb|AELG01005682.1| | 34339 | 45398 | 16 | 2 |
| XLOC\_001965 | gi|313875954|gb|AELG01005682.1| | 54157 | 57551 | 6 | 1 |
| XLOC\_001978 XLOC\_001984 XLOC\_001977 | gi|313875956|gb|AELG01005680.1| | 47158 | 64029 | 29 | 1 |
| XLOC\_001982 | gi|313875956|gb|AELG01005680.1| | 40819 | 44874 | 4 | 3 |
| XLOC\_001985 | gi|313875956|gb|AELG01005680.1| | 64421 | 71717 | 19 | 1 |
| XLOC\_001988 XLOC\_001989 XLOC\_001990 | gi|313875963|gb|AELG01005673.1| | 1 | 14257 | 49 | 3 |
| XLOC\_002017 | gi|313875975|gb|AELG01005661.1| | 36190 | 43169 | 10 | 3 |
| XLOC\_002023 | gi|313875976|gb|AELG01005660.1| | 48174 | 60476 | 21 | 1 |
| XLOC\_002033 | gi|313875980|gb|AELG01005656.1| | 10827 | 16106 | 10 | 1 |
| XLOC\_002063 | gi|313875993|gb|AELG01005643.1| | 9303 | 14240 | 5 | 1 |
| XLOC\_002069 | gi|313875996|gb|AELG01005641.1| | 76971 | 110718 | 8 | 1 |
| XLOC\_002077 | gi|313875997|gb|AELG01005640.1| | 3739 | 12054 | 14 | 3 |
| XLOC\_002081 | gi|313875998|gb|AELG01005639.1| | 57873 | 62520 | 5 | 2 |
| XLOC\_002092 | gi|313876002|gb|AELG01005635.1| | 12390 | 29916 | 10 | 1 |
| XLOC\_002096 | gi|313876003|gb|AELG01005634.1| | 43755 | 50552 | 5 | 2 |
| XLOC\_002097 | gi|313876004|gb|AELG01005633.1| | 7 | 20501 | 13 | 2 |
| XLOC\_002098 | gi|313876005|gb|AELG01005632.1| | 21597 | 38428 | 8 | 1 |
| XLOC\_002106 | gi|313876009|gb|AELG01005628.1| | 41825 | 54975 | 8 | 2 |
| XLOC\_002112 | gi|313876014|gb|AELG01005623.1| | 60535 | 73842 | 13 | 1 |
| XLOC\_002138 | gi|313876031|gb|AELG01005606.1| | 27898 | 37990 | 14 | 2 |
| XLOC\_002139 | gi|313876031|gb|AELG01005606.1| | 21 | 2044 | 4 | 2 |
| XLOC\_002165 | gi|313876043|gb|AELG01005594.1| | 28758 | 38722 | 8 | 1 |
| XLOC\_002169 | gi|313876052|gb|AELG01005585.1| | 9030 | 35954 | 15 | 4 |
| XLOC\_002170 | gi|313876055|gb|AELG01005582.1| | 2273 | 9322 | 29 | 1 |
| XLOC\_002172 | gi|313876055|gb|AELG01005582.1| | 1 | 1914 | 9 | 1 |
| XLOC\_002203 | gi|313876118|gb|AELG01005521.1| | 40560 | 47569 | 5 | 2 |
| XLOC\_002355 XLOC\_002356 | gi|313876200|gb|AELG01005441.1| | 12 | 13152 | 7 | 1 |
| XLOC\_002360 | gi|313876201|gb|AELG01005440.1| | 20419 | 26354 | 17 | 1 |
| XLOC\_002368 | gi|313876202|gb|AELG01005439.1| | 1546 | 8556 | 9 | 2 |
| XLOC\_002374 | gi|313876206|gb|AELG01005435.1| | 15372 | 75762 | 12 | 1 |
| XLOC\_002383 XLOC\_002382 | gi|313876208|gb|AELG01005433.1| | 4957 | 52732 | 11 | 3 |
| XLOC\_002384 | gi|313876208|gb|AELG01005433.1| | 53208 | 55623 | 3 | 1 |
| XLOC\_002389 XLOC\_002388 | gi|313876209|gb|AELG01005432.1| | 563 | 13873 | 27 | 3 |
| XLOC\_002390 XLOC\_002391 | gi|313876210|gb|AELG01005431.1| | 112 | 27916 | 30 | 4 |
| XLOC\_002405 XLOC\_002399 | gi|313876211|gb|AELG01005430.1| | 42201 | 59494 | 38 | 1 |
| XLOC\_002418 | gi|313876215|gb|AELG01005426.1| | 18542 | 28542 | 5 | 2 |
| XLOC\_002421 | gi|313876217|gb|AELG01005425.1| | 37367 | 50705 | 7 | 1 |
| XLOC\_002423 | gi|313876227|gb|AELG01005415.1| | 21791 | 34240 | 12 | 5 |
| XLOC\_002429 | gi|313876236|gb|AELG01005406.1| | 72158 | 85490 | 15 | 1 |
| XLOC\_002430 | gi|313876236|gb|AELG01005406.1| | 83930 | 100610 | 29 | 4 |
| XLOC\_002441 | gi|313876238|gb|AELG01005404.1| | 86 | 21649 | 34 | 3 |
| XLOC\_002447 XLOC\_002454 | gi|313876239|gb|AELG01005403.1| | 21638 | 26870 | 23 | 1 |
| XLOC\_002455 | gi|313876239|gb|AELG01005403.1| | 27451 | 30924 | 11 | 1 |
| XLOC\_002465 | gi|313876242|gb|AELG01005400.1| | 12805 | 17706 | 7 | 2 |
| XLOC\_002476 | gi|313876244|gb|AELG01005398.1| | 2206 | 19394 | 22 | 5 |
| XLOC\_002531 | gi|313876266|gb|AELG01005377.1| | 13007 | 16545 | 9 | 1 |
| XLOC\_002545 | gi|313876268|gb|AELG01005375.1| | 33619 | 88570 | 9 | 8 |
| XLOC\_002553 | gi|313876270|gb|AELG01005373.1| | 20998 | 34359 | 14 | 1 |
| XLOC\_002556 | gi|313876271|gb|AELG01005372.1| | 58329 | 62923 | 14 | 2 |
| XLOC\_002567 | gi|313876272|gb|AELG01005371.1| | 28087 | 38660 | 19 | 2 |
| XLOC\_002574 | gi|313876274|gb|AELG01005369.1| | 43 | 29543 | 57 | 24 |
| XLOC\_002583 | gi|313876276|gb|AELG01005367.1| | 246194 | 272271 | 16 | 4 |
| XLOC\_002606 | gi|313876278|gb|AELG01005365.1| | 24043 | 30390 | 7 | 1 |
| XLOC\_002610 XLOC\_002604 | gi|313876278|gb|AELG01005365.1| | 9923 | 18113 | 21 | 2 |
| XLOC\_002614 XLOC\_002616 | gi|313876279|gb|AELG01005364.1| | 36784 | 54562 | 17 | 1 |
| XLOC\_002619 | gi|313876282|gb|AELG01005362.1| | 1 | 101766 | 21 | 4 |
| XLOC\_002624 XLOC\_002621 | gi|313876283|gb|AELG01005361.1| | 30185 | 46472 | 20 | 2 |
| XLOC\_002629 | gi|313876285|gb|AELG01005359.1| | 10124 | 25970 | 11 | 2 |
| XLOC\_002648 | gi|313876291|gb|AELG01005353.1| | 83393 | 88469 | 14 | 1 |
| XLOC\_002670 | gi|313876297|gb|AELG01005347.1| | 8409 | 16100 | 9 | 1 |
| XLOC\_002676 | gi|313876298|gb|AELG01005346.1| | 14867 | 151451 | 19 | 1 |
| XLOC\_002688 XLOC\_002689 | gi|313876301|gb|AELG01005343.1| | 75 | 62733 | 15 | 1 |
| XLOC\_002701 XLOC\_002700 | gi|313876307|gb|AELG01005338.1| | 4579 | 13768 | 18 | 1 |
| XLOC\_002710 | gi|313876310|gb|AELG01005335.1| | 45818 | 59515 | 8 | 2 |
| XLOC\_002711 | gi|313876310|gb|AELG01005335.1| | 63618 | 71827 | 8 | 1 |
| XLOC\_002720 XLOC\_002718 XLOC\_002719 | gi|313876311|gb|AELG01005334.1| | 45588 | 90725 | 53 | 5 |
| XLOC\_002724 | gi|313876312|gb|AELG01005333.1| | 8452 | 28162 | 17 | 9 |
| XLOC\_002729 | gi|313876312|gb|AELG01005333.1| | 82496 | 97012 | 8 | 4 |
| XLOC\_002735 XLOC\_002745 | gi|313876312|gb|AELG01005333.1| | 136158 | 152857 | 26 | 4 |
| XLOC\_002736 XLOC\_002746 | gi|313876312|gb|AELG01005333.1| | 154191 | 172306 | 17 | 1 |
| XLOC\_002754 XLOC\_002763 XLOC\_002762 | gi|313876313|gb|AELG01005332.1| | 30888 | 49784 | 25 | 1 |
| XLOC\_002776 | gi|313876317|gb|AELG01005328.1| | 25119 | 41806 | 11 | 2 |
| XLOC\_002777 XLOC\_002781 | gi|313876317|gb|AELG01005328.1| | 65252 | 85228 | 29 | 6 |
| XLOC\_002778 | gi|313876317|gb|AELG01005328.1| | 816 | 12691 | 11 | 1 |
| XLOC\_002784 | gi|313876318|gb|AELG01005327.1| | 13179 | 29354 | 12 | 1 |
| XLOC\_002785 XLOC\_002788 | gi|313876318|gb|AELG01005327.1| | 13459 | 20641 | 19 | 1 |
| XLOC\_002800 XLOC\_002797 | gi|313876322|gb|AELG01005323.1| | 706 | 9318 | 15 | 2 |
| XLOC\_002801 XLOC\_002798 | gi|313876322|gb|AELG01005323.1| | 9494 | 19127 | 26 | 1 |
| XLOC\_002805 XLOC\_002807 | gi|313876325|gb|AELG01005320.1| | 10217 | 14273 | 14 | 1 |
| XLOC\_002813 | gi|313876328|gb|AELG01005317.1| | 293 | 25310 | 18 | 1 |
| XLOC\_002815 | gi|313876328|gb|AELG01005317.1| | 28445 | 45396 | 17 | 1 |
| XLOC\_002816 | gi|313876328|gb|AELG01005317.1| | 49850 | 55120 | 10 | 1 |
| XLOC\_002822 | gi|313876329|gb|AELG01005316.1| | 13133 | 45608 | 19 | 2 |
| XLOC\_002834 XLOC\_002826 | gi|313876329|gb|AELG01005316.1| | 51738 | 74527 | 16 | 1 |
| XLOC\_002837 | gi|313876329|gb|AELG01005316.1| | 93136 | 99915 | 10 | 3 |
| XLOC\_002839 XLOC\_002843 | gi|313876330|gb|AELG01005315.1| | 55938 | 59596 | 22 | 1 |
| XLOC\_002841 | gi|313876330|gb|AELG01005315.1| | 7533 | 21413 | 25 | 7 |
| XLOC\_002842 | gi|313876330|gb|AELG01005315.1| | 21904 | 54410 | 25 | 7 |
| XLOC\_002847 | gi|313876332|gb|AELG01005313.1| | 8 | 51211 | 23 | 5 |
| XLOC\_002852 XLOC\_002859 XLOC\_002858 | gi|313876333|gb|AELG01005312.1| | 37669 | 46532 | 34 | 3 |
| XLOC\_002893 XLOC\_002892 | gi|313876337|gb|AELG01005308.1| | 28075 | 68454 | 21 | 6 |
| XLOC\_002900 | gi|313876340|gb|AELG01005305.1| | 4 | 6640 | 18 | 1 |
| XLOC\_002914 XLOC\_002910 | gi|313876342|gb|AELG01005303.1| | 45316 | 58547 | 36 | 1 |
| XLOC\_002918 | gi|313876343|gb|AELG01005302.1| | 5865 | 26603 | 4 | 1 |
| XLOC\_002928 | gi|313876352|gb|AELG01005294.1| | 11427 | 19482 | 7 | 3 |
| XLOC\_002933 | gi|313876354|gb|AELG01005292.1| | 2578 | 17404 | 13 | 1 |
| XLOC\_002935 | gi|313876354|gb|AELG01005292.1| | 22175 | 28032 | 8 | 1 |
| XLOC\_002937 | gi|313876355|gb|AELG01005291.1| | 71441 | 186036 | 54 | 33 |
| XLOC\_002944 XLOC\_002939 | gi|313876355|gb|AELG01005291.1| | 193699 | 204513 | 15 | 2 |
| XLOC\_002945 XLOC\_002941 XLOC\_002940 XLOC\_002942 | gi|313876355|gb|AELG01005291.1| | 205070 | 226220 | 79 | 2 |
| XLOC\_003005 | gi|313876429|gb|AELG01005218.1| | 3442 | 10526 | 14 | 2 |
| XLOC\_003015 | gi|313876434|gb|AELG01005213.1| | 9854 | 14720 | 5 | 2 |
| XLOC\_003018 | gi|313876436|gb|AELG01005211.1| | 22089 | 30612 | 13 | 1 |
| XLOC\_003025 | gi|313876451|gb|AELG01005196.1| | 13722 | 45718 | 7 | 3 |
| XLOC\_003032 XLOC\_003036 XLOC\_003035 | gi|313876452|gb|AELG01005195.1| | 29283 | 45963 | 57 | 4 |
| XLOC\_003034 | gi|313876452|gb|AELG01005195.1| | 17250 | 23998 | 15 | 1 |
| XLOC\_003046 | gi|313876453|gb|AELG01005194.1| | 62864 | 89553 | 10 | 4 |
| XLOC\_003053 | gi|313876456|gb|AELG01005191.1| | 754 | 5911 | 12 | 1 |
| XLOC\_003054 | gi|313876456|gb|AELG01005191.1| | 12334 | 14986 | 3 | 2 |
| XLOC\_003061 XLOC\_003075 XLOC\_003065 | gi|313876458|gb|AELG01005189.1| | 16205 | 28674 | 22 | 5 |
| XLOC\_003068 XLOC\_003063 | gi|313876458|gb|AELG01005189.1| | 38315 | 44446 | 15 | 2 |
| XLOC\_003080 | gi|313876459|gb|AELG01005188.1| | 18752 | 27603 | 7 | 4 |
| XLOC\_003083 | gi|313876459|gb|AELG01005188.1| | 43948 | 73814 | 7 | 2 |
| XLOC\_003123 XLOC\_003124 | gi|313876470|gb|AELG01005178.1| | 662 | 5699 | 8 | 1 |
| XLOC\_003131 | gi|313876478|gb|AELG01005170.1| | 2119 | 7087 | 8 | 1 |
| XLOC\_003145 | gi|313876481|gb|AELG01005167.1| | 301 | 12441 | 17 | 4 |
| XLOC\_003157 | gi|313876488|gb|AELG01005160.1| | 97864 | 110982 | 24 | 3 |
| XLOC\_003159 | gi|313876491|gb|AELG01005157.1| | 2199 | 14514 | 23 | 1 |
| XLOC\_003171 | gi|313876494|gb|AELG01005154.1| | 176835 | 190054 | 12 | 1 |
| XLOC\_003183 XLOC\_003186 | gi|313876501|gb|AELG01005147.1| | 38192 | 68864 | 21 | 5 |
| XLOC\_003212 | gi|313876508|gb|AELG01005141.1| | 82 | 4533 | 7 | 1 |
| XLOC\_003268 | gi|313876548|gb|AELG01005102.1| | 25774 | 50412 | 4 | 1 |
| XLOC\_003270 | gi|313876552|gb|AELG01005098.1| | 3826 | 45066 | 8 | 3 |
| XLOC\_003276 | gi|313876554|gb|AELG01005096.1| | 216 | 97800 | 20 | 1 |
| XLOC\_003312 XLOC\_003314 | gi|313876568|gb|AELG01005082.1| | 21672 | 41623 | 18 | 4 |
| XLOC\_003322 | gi|313876572|gb|AELG01005078.1| | 4253 | 9066 | 10 | 1 |
| XLOC\_003347 | gi|313876594|gb|AELG01005057.1| | 24020 | 27342 | 10 | 2 |
| XLOC\_003366 | gi|313876615|gb|AELG01005036.1| | 15803 | 27812 | 19 | 4 |
| XLOC\_003371 XLOC\_003372 | gi|313876616|gb|AELG01005035.1| | 15205 | 24153 | 12 | 1 |
| XLOC\_003406 | gi|313876628|gb|AELG01005023.1| | 23 | 56540 | 6 | 2 |
| XLOC\_003408 | gi|313876629|gb|AELG01005022.1| | 166 | 5023 | 14 | 1 |
| XLOC\_003409 | gi|313876629|gb|AELG01005022.1| | 6369 | 15083 | 14 | 3 |
| XLOC\_003410 | gi|313876630|gb|AELG01005021.1| | 45677 | 62482 | 33 | 5 |
| XLOC\_003433 | gi|313876635|gb|AELG01005016.1| | 5977 | 11394 | 6 | 1 |
| XLOC\_003434 | gi|313876635|gb|AELG01005016.1| | 14047 | 41762 | 8 | 4 |
| XLOC\_003447 | gi|313876637|gb|AELG01005014.1| | 55278 | 59637 | 14 | 1 |
| XLOC\_003467 | gi|313876639|gb|AELG01005012.1| | 29202 | 44361 | 27 | 3 |
| XLOC\_003472 | gi|313876639|gb|AELG01005012.1| | 63908 | 68577 | 6 | 1 |
| XLOC\_003491 | gi|313876640|gb|AELG01005011.1| | 24681 | 45619 | 24 | 4 |
| XLOC\_003508 | gi|313876642|gb|AELG01005009.1| | 64 | 3233 | 3 | 2 |
| XLOC\_003515 | gi|313876643|gb|AELG01005008.1| | 11398 | 15554 | 8 | 2 |
| XLOC\_003525 | gi|313876644|gb|AELG01005007.1| | 37359 | 43465 | 13 | 1 |
| XLOC\_003537 XLOC\_003522 | gi|313876644|gb|AELG01005007.1| | 19180 | 27494 | 23 | 3 |
| XLOC\_003541 XLOC\_003532 | gi|313876644|gb|AELG01005007.1| | 89625 | 100833 | 41 | 1 |
| XLOC\_003570 | gi|313876650|gb|AELG01005001.1| | 6750 | 15114 | 11 | 1 |
| XLOC\_003598 | gi|313876677|gb|AELG01004975.1| | 44524 | 46677 | 5 | 1 |
| XLOC\_003615 | gi|313876678|gb|AELG01004974.1| | 35 | 26817 | 6 | 2 |
| XLOC\_003620 | gi|313876679|gb|AELG01004973.1| | 101654 | 113325 | 32 | 10 |
| XLOC\_003629 | gi|313876684|gb|AELG01004968.1| | 10215 | 19991 | 6 | 2 |
| XLOC\_003641 XLOC\_003650 | gi|313876686|gb|AELG01004967.1| | 79423 | 90168 | 27 | 1 |
| XLOC\_003643 XLOC\_003636 | gi|313876686|gb|AELG01004967.1| | 6921 | 14283 | 33 | 1 |
| XLOC\_003654 | gi|313876687|gb|AELG01004966.1| | 10198 | 31935 | 26 | 3 |
| XLOC\_003692 | gi|313876692|gb|AELG01004961.1| | 618 | 9628 | 25 | 1 |
| XLOC\_003693 | gi|313876692|gb|AELG01004961.1| | 13894 | 18694 | 4 | 1 |
| XLOC\_003704 | gi|313876693|gb|AELG01004960.1| | 45107 | 54742 | 13 | 2 |
| XLOC\_003705 | gi|313876693|gb|AELG01004960.1| | 55749 | 61584 | 14 | 3 |
| XLOC\_003719 | gi|313876694|gb|AELG01004959.1| | 37691 | 42215 | 13 | 2 |
| XLOC\_003731 | gi|313876699|gb|AELG01004954.1| | 7425 | 16706 | 5 | 2 |
| XLOC\_003733 XLOC\_003732 XLOC\_003735 XLOC\_003734 XLOC\_003738 | gi|313876699|gb|AELG01004954.1| | 16795 | 43586 | 42 | 1 |
| XLOC\_003743 | gi|313876708|gb|AELG01004945.1| | 7673 | 45119 | 17 | 2 |
| XLOC\_003749 | gi|313876708|gb|AELG01004945.1| | 77445 | 82450 | 8 | 2 |
| XLOC\_003750 | gi|313876708|gb|AELG01004945.1| | 45206 | 49669 | 15 | 1 |
| XLOC\_003765 | gi|313876711|gb|AELG01004942.1| | 76414 | 81025 | 15 | 1 |
| XLOC\_003811 XLOC\_003815 | gi|313876777|gb|AELG01004878.1| | 46029 | 57696 | 20 | 3 |
| XLOC\_003828 XLOC\_003835 | gi|313876779|gb|AELG01004876.1| | 59415 | 68191 | 24 | 1 |
| XLOC\_003846 XLOC\_003834 | gi|313876779|gb|AELG01004876.1| | 183435 | 244165 | 33 | 1 |
| XLOC\_003847 | gi|313876779|gb|AELG01004876.1| | 244321 | 247670 | 4 | 1 |
| XLOC\_003862 XLOC\_003867 | gi|313876782|gb|AELG01004873.1| | 9752 | 32923 | 15 | 2 |
| XLOC\_003863 | gi|313876782|gb|AELG01004873.1| | 86536 | 92948 | 3 | 1 |
| XLOC\_003866 | gi|313876782|gb|AELG01004873.1| | 106441 | 113174 | 5 | 2 |
| XLOC\_003869 | gi|313876782|gb|AELG01004873.1| | 81140 | 86346 | 14 | 2 |
| XLOC\_003883 XLOC\_003881 | gi|313876785|gb|AELG01004870.1| | 8 | 29948 | 8 | 1 |
| XLOC\_003885 | gi|313876786|gb|AELG01004869.1| | 17577 | 38303 | 9 | 4 |
| XLOC\_003889 XLOC\_003893 XLOC\_003894 | gi|313876794|gb|AELG01004862.1| | 18348 | 40957 | 50 | 5 |
| XLOC\_003896 | gi|313876794|gb|AELG01004862.1| | 53146 | 120521 | 14 | 2 |
| XLOC\_003901 XLOC\_003905 | gi|313876795|gb|AELG01004861.1| | 8285 | 24050 | 25 | 3 |
| XLOC\_003917 XLOC\_003912 | gi|313876796|gb|AELG01004860.1| | 55191 | 60160 | 13 | 1 |
| XLOC\_003929 | gi|313876798|gb|AELG01004858.1| | 137395 | 142622 | 9 | 1 |
| XLOC\_003931 | gi|313876798|gb|AELG01004858.1| | 173140 | 177480 | 9 | 1 |
| XLOC\_003933 XLOC\_003949 | gi|313876798|gb|AELG01004858.1| | 194525 | 204599 | 35 | 3 |
| XLOC\_003934 | gi|313876798|gb|AELG01004858.1| | 205635 | 221107 | 15 | 7 |
| XLOC\_003938 XLOC\_003925 | gi|313876798|gb|AELG01004858.1| | 6182 | 12707 | 11 | 2 |
| XLOC\_003939 | gi|313876798|gb|AELG01004858.1| | 24985 | 114130 | 15 | 4 |
| XLOC\_003944 XLOC\_003930 | gi|313876798|gb|AELG01004858.1| | 155108 | 166693 | 31 | 2 |
| XLOC\_003950 XLOC\_003935 | gi|313876798|gb|AELG01004858.1| | 221579 | 226008 | 28 | 1 |
| XLOC\_003976 | gi|313876804|gb|AELG01004852.1| | 60003 | 63606 | 6 | 1 |
| XLOC\_003994 | gi|313876805|gb|AELG01004851.1| | 30321 | 39639 | 17 | 2 |
| XLOC\_004009 | gi|313876806|gb|AELG01004850.1| | 23591 | 38548 | 15 | 1 |
| XLOC\_004012 XLOC\_004008 | gi|313876806|gb|AELG01004850.1| | 16033 | 21478 | 18 | 1 |
| XLOC\_004027 XLOC\_004023 | gi|313876819|gb|AELG01004837.1| | 59335 | 68144 | 14 | 2 |
| XLOC\_004057 | gi|313876839|gb|AELG01004817.1| | 2 | 5124 | 5 | 3 |
| XLOC\_004060 XLOC\_004062 | gi|313876845|gb|AELG01004811.1| | 11850 | 22446 | 4 | 2 |
| XLOC\_004109 | gi|313876893|gb|AELG01004765.1| | 14470 | 59398 | 30 | 1 |
| XLOC\_004112 | gi|313876893|gb|AELG01004765.1| | 62277 | 79981 | 2 | 2 |
| XLOC\_004118 | gi|313876894|gb|AELG01004764.1| | 39300 | 45943 | 14 | 2 |
| XLOC\_004155 | gi|313876909|gb|AELG01004750.1| | 444 | 8767 | 13 | 2 |
| XLOC\_004190 | gi|313876946|gb|AELG01004713.1| | 2873 | 11218 | 11 | 1 |
| XLOC\_004224 XLOC\_004223 | gi|313876968|gb|AELG01004692.1| | 1502 | 18193 | 42 | 2 |
| XLOC\_004227 | gi|313876969|gb|AELG01004691.1| | 18684 | 193581 | 13 | 4 |
| XLOC\_004247 | gi|313876975|gb|AELG01004685.1| | 58808 | 83578 | 16 | 1 |
| XLOC\_004253 | gi|313876977|gb|AELG01004683.1| | 78192 | 92213 | 9 | 1 |
| XLOC\_004254 | gi|313876977|gb|AELG01004683.1| | 146106 | 164062 | 7 | 1 |
| XLOC\_004261 | gi|313876978|gb|AELG01004682.1| | 32224 | 43505 | 31 | 1 |
| XLOC\_004280 | gi|313876979|gb|AELG01004681.1| | 1182 | 16185 | 7 | 2 |
| XLOC\_004282 | gi|313876980|gb|AELG01004680.1| | 76291 | 81673 | 11 | 1 |
| XLOC\_004286 XLOC\_004288 | gi|313876980|gb|AELG01004680.1| | 97206 | 104640 | 26 | 1 |
| XLOC\_004292 | gi|313876982|gb|AELG01004678.1| | 25394 | 41508 | 14 | 2 |
| XLOC\_004294 XLOC\_004298 | gi|313876982|gb|AELG01004678.1| | 42066 | 63057 | 24 | 3 |
| XLOC\_004326 | gi|313876992|gb|AELG01004668.1| | 49462 | 51428 | 6 | 1 |
| XLOC\_004335 | gi|313876997|gb|AELG01004663.1| | 92685 | 98455 | 15 | 1 |
| XLOC\_004347 | gi|313877009|gb|AELG01004651.1| | 4782 | 9979 | 9 | 1 |
| XLOC\_004348 | gi|313877009|gb|AELG01004651.1| | 10189 | 21216 | 29 | 4 |
| XLOC\_004355 | gi|313877016|gb|AELG01004645.1| | 92388 | 101971 | 8 | 2 |
| XLOC\_004357 XLOC\_004366 XLOC\_004371 | gi|313877016|gb|AELG01004645.1| | 106208 | 111749 | 23 | 1 |
| XLOC\_004360 | gi|313877016|gb|AELG01004645.1| | 30444 | 50578 | 17 | 1 |
| XLOC\_004388 | gi|313877020|gb|AELG01004641.1| | 24677 | 32711 | 14 | 1 |
| XLOC\_004421 | gi|313877044|gb|AELG01004617.1| | 77838 | 96374 | 28 | 4 |
| XLOC\_004428 | gi|313877047|gb|AELG01004614.1| | 102697 | 118834 | 25 | 9 |
| XLOC\_004432 | gi|313877047|gb|AELG01004614.1| | 57802 | 70383 | 28 | 1 |
| XLOC\_004445 | gi|313877050|gb|AELG01004611.1| | 17341 | 59792 | 12 | 2 |
| XLOC\_004465 XLOC\_004462 | gi|313877055|gb|AELG01004606.1| | 16086 | 43449 | 13 | 2 |
| XLOC\_004470 | gi|313877056|gb|AELG01004605.1| | 32 | 6895 | 5 | 3 |
| XLOC\_004471 | gi|313877056|gb|AELG01004605.1| | 7560 | 25705 | 6 | 2 |
| XLOC\_004474 | gi|313877057|gb|AELG01004604.1| | 69620 | 77804 | 19 | 3 |
| XLOC\_004476 | gi|313877057|gb|AELG01004604.1| | 14775 | 68783 | 12 | 3 |
| XLOC\_004480 | gi|313877058|gb|AELG01004603.1| | 39400 | 45990 | 19 | 3 |
| XLOC\_004501 XLOC\_004504 | gi|313877059|gb|AELG01004602.1| | 804 | 11937 | 26 | 6 |
| XLOC\_004502 XLOC\_004505 | gi|313877059|gb|AELG01004602.1| | 12262 | 21142 | 15 | 1 |
| XLOC\_004518 XLOC\_004515 | gi|313877064|gb|AELG01004597.1| | 11064 | 18298 | 23 | 2 |
| XLOC\_004523 | gi|313877068|gb|AELG01004593.1| | 34871 | 45342 | 10 | 1 |
| XLOC\_004526 XLOC\_004522 | gi|313877068|gb|AELG01004593.1| | 7829 | 16698 | 38 | 5 |
| XLOC\_004542 | gi|313877079|gb|AELG01004583.1| | 34103 | 41973 | 11 | 3 |
| XLOC\_004552 | gi|313877081|gb|AELG01004581.1| | 5626 | 31319 | 10 | 1 |
| XLOC\_004554 | gi|313877081|gb|AELG01004581.1| | 34170 | 42527 | 12 | 2 |
| XLOC\_004565 XLOC\_004567 | gi|313877086|gb|AELG01004576.1| | 94 | 9197 | 18 | 1 |
| XLOC\_004572 | gi|313877087|gb|AELG01004575.1| | 74785 | 87549 | 20 | 8 |
| XLOC\_004575 | gi|313877090|gb|AELG01004572.1| | 9383 | 13503 | 12 | 1 |
| XLOC\_004593 | gi|313877101|gb|AELG01004561.1| | 67146 | 102643 | 19 | 4 |
| XLOC\_004609 | gi|313877104|gb|AELG01004558.1| | 57240 | 63492 | 27 | 1 |
| XLOC\_004626 | gi|313877115|gb|AELG01004548.1| | 79786 | 230062 | 17 | 7 |
| XLOC\_004632 | gi|313877116|gb|AELG01004547.1| | 49787 | 76697 | 44 | 2 |
| XLOC\_004635 XLOC\_004630 | gi|313877116|gb|AELG01004547.1| | 10451 | 25947 | 38 | 3 |
| XLOC\_004638 | gi|313877116|gb|AELG01004547.1| | 78430 | 90030 | 21 | 3 |
| XLOC\_004645 | gi|313877117|gb|AELG01004546.1| | 52877 | 57860 | 17 | 1 |
| XLOC\_004646 | gi|313877117|gb|AELG01004546.1| | 58497 | 63825 | 2 | 2 |
| XLOC\_004655 | gi|313877120|gb|AELG01004543.1| | 57789 | 63752 | 14 | 1 |
| XLOC\_004670 | gi|313877127|gb|AELG01004536.1| | 695 | 44760 | 18 | 3 |
| XLOC\_004684 XLOC\_004686 | gi|313877128|gb|AELG01004535.1| | 99884 | 120522 | 32 | 5 |
| XLOC\_004710 | gi|313877145|gb|AELG01004520.1| | 42635 | 49298 | 8 | 1 |
| XLOC\_004711 | gi|313877145|gb|AELG01004520.1| | 11196 | 41408 | 21 | 8 |
| XLOC\_004721 | gi|313877150|gb|AELG01004515.1| | 31570 | 36608 | 8 | 2 |
| XLOC\_004762 | gi|313877155|gb|AELG01004510.1| | 62332 | 66717 | 9 | 1 |
| XLOC\_004770 | gi|313877158|gb|AELG01004507.1| | 104509 | 121790 | 5 | 1 |
| XLOC\_004789 XLOC\_004784 | gi|313877162|gb|AELG01004505.1| | 61572 | 77197 | 27 | 11 |
| XLOC\_004796 | gi|313877163|gb|AELG01004504.1| | 12415 | 16692 | 7 | 1 |
| XLOC\_004818 XLOC\_004815 | gi|313877168|gb|AELG01004499.1| | 46060 | 81625 | 46 | 4 |
| XLOC\_004819 | gi|313877168|gb|AELG01004499.1| | 90020 | 107939 | 11 | 5 |
| XLOC\_004836 XLOC\_004845 | gi|313877170|gb|AELG01004497.1| | 19234 | 34504 | 11 | 4 |
| XLOC\_004842 | gi|313877170|gb|AELG01004497.1| | 16 | 13263 | 21 | 10 |
| XLOC\_004848 XLOC\_004841 | gi|313877170|gb|AELG01004497.1| | 60562 | 63581 | 25 | 1 |
| XLOC\_004850 | gi|313877171|gb|AELG01004496.1| | 7589 | 15240 | 21 | 2 |
| XLOC\_004852 | gi|313877171|gb|AELG01004496.1| | 30413 | 52805 | 32 | 8 |
| XLOC\_004853 | gi|313877171|gb|AELG01004496.1| | 53458 | 73255 | 42 | 19 |
| XLOC\_004854 XLOC\_004862 | gi|313877171|gb|AELG01004496.1| | 81994 | 89843 | 44 | 2 |
| XLOC\_004857 XLOC\_004863 | gi|313877171|gb|AELG01004496.1| | 96491 | 104621 | 27 | 2 |
| XLOC\_004869 | gi|313877172|gb|AELG01004495.1| | 25805 | 49237 | 44 | 25 |
| XLOC\_004897 | gi|313877181|gb|AELG01004486.1| | 5042 | 15861 | 3 | 2 |
| XLOC\_004908 | gi|313877182|gb|AELG01004485.1| | 31809 | 45347 | 12 | 2 |
| XLOC\_004909 | gi|313877182|gb|AELG01004485.1| | 50794 | 63923 | 5 | 3 |
| XLOC\_004914 XLOC\_004920 | gi|313877182|gb|AELG01004485.1| | 188600 | 194326 | 22 | 1 |
| XLOC\_004917 | gi|313877182|gb|AELG01004485.1| | 45491 | 49602 | 9 | 1 |
| XLOC\_004918 | gi|313877182|gb|AELG01004485.1| | 138949 | 168445 | 20 | 4 |
| XLOC\_004943 XLOC\_004947 | gi|313877203|gb|AELG01004466.1| | 4975 | 14416 | 21 | 2 |
| XLOC\_004970 | gi|313877209|gb|AELG01004460.1| | 19354 | 29843 | 4 | 3 |
| XLOC\_004983 XLOC\_004980 | gi|313877224|gb|AELG01004445.1| | 152181 | 158423 | 14 | 2 |
| XLOC\_004988 | gi|313877227|gb|AELG01004442.1| | 1022 | 23073 | 46 | 12 |
| XLOC\_004995 | gi|313877230|gb|AELG01004439.1| | 2052 | 9607 | 5 | 2 |
| XLOC\_004999 | gi|313877231|gb|AELG01004438.1| | 28576 | 32454 | 11 | 1 |
| XLOC\_005002 XLOC\_004998 | gi|313877231|gb|AELG01004438.1| | 16335 | 28459 | 26 | 1 |
| XLOC\_005009 | gi|313877265|gb|AELG01004437.1| | 63689 | 102029 | 4 | 1 |
| XLOC\_005027 XLOC\_005024 | gi|313877267|gb|AELG01004435.1| | 4523 | 19181 | 31 | 4 |
| XLOC\_005032 | gi|313877268|gb|AELG01004434.1| | 58562 | 67976 | 17 | 1 |
| XLOC\_005033 | gi|313877268|gb|AELG01004434.1| | 37897 | 42140 | 9 | 1 |
| XLOC\_005084 | gi|313877331|gb|AELG01004389.1| | 37 | 3250 | 7 | 1 |
| XLOC\_005122 XLOC\_005121 | gi|313877366|gb|AELG01004359.1| | 16251 | 30614 | 30 | 4 |
| XLOC\_005130 | gi|313877372|gb|AELG01004353.1| | 2471 | 50732 | 3 | 1 |
| XLOC\_005145 | gi|313877374|gb|AELG01004351.1| | 193603 | 198407 | 11 | 2 |
| XLOC\_005148 | gi|313877374|gb|AELG01004351.1| | 234171 | 251600 | 23 | 2 |
| XLOC\_005149 | gi|313877374|gb|AELG01004351.1| | 257556 | 264904 | 3 | 2 |
| XLOC\_005163 | gi|313877374|gb|AELG01004351.1| | 201864 | 215733 | 13 | 1 |
| XLOC\_005164 | gi|313877374|gb|AELG01004351.1| | 220979 | 229624 | 21 | 1 |
| XLOC\_005165 | gi|313877374|gb|AELG01004351.1| | 266084 | 271097 | 11 | 2 |
| XLOC\_005186 | gi|313877376|gb|AELG01004349.1| | 42558 | 50567 | 6 | 5 |
| XLOC\_005199 | gi|313877384|gb|AELG01004344.1| | 7836 | 138437 | 34 | 1 |
| XLOC\_005202 XLOC\_005196 | gi|313877384|gb|AELG01004344.1| | 190186 | 195826 | 17 | 2 |
| XLOC\_005243 | gi|313877390|gb|AELG01004338.1| | 14401 | 29112 | 4 | 1 |
| XLOC\_005250 | gi|313877392|gb|AELG01004336.1| | 14417 | 19243 | 11 | 2 |
| XLOC\_005279 | gi|313877398|gb|AELG01004330.1| | 2854 | 26416 | 11 | 1 |
| XLOC\_005290 | gi|313877402|gb|AELG01004326.1| | 4428 | 16019 | 14 | 3 |
| XLOC\_005291 XLOC\_005293 | gi|313877402|gb|AELG01004326.1| | 19075 | 27869 | 19 | 1 |
| XLOC\_005332 | gi|313877436|gb|AELG01004299.1| | 15808 | 18652 | 6 | 1 |
| XLOC\_005333 | gi|313877436|gb|AELG01004299.1| | 26385 | 32731 | 9 | 1 |
| XLOC\_005337 | gi|313877436|gb|AELG01004299.1| | 21645 | 25696 | 7 | 1 |
| XLOC\_005345 | gi|313877438|gb|AELG01004297.1| | 11532 | 17462 | 14 | 1 |
| XLOC\_005346 XLOC\_005342 | gi|313877438|gb|AELG01004297.1| | 18052 | 34553 | 13 | 1 |
| XLOC\_005350 | gi|313877441|gb|AELG01004294.1| | 17749 | 22699 | 3 | 1 |
| XLOC\_005354 | gi|313877444|gb|AELG01004291.1| | 35 | 11602 | 8 | 2 |
| XLOC\_005356 | gi|313877444|gb|AELG01004291.1| | 32442 | 51368 | 6 | 3 |
| XLOC\_005357 | gi|313877444|gb|AELG01004291.1| | 55579 | 86780 | 20 | 2 |
| XLOC\_005358 | gi|313877445|gb|AELG01004290.1| | 36 | 4902 | 8 | 1 |
| XLOC\_005376 | gi|313877445|gb|AELG01004290.1| | 37662 | 41880 | 12 | 1 |
| XLOC\_005380 XLOC\_005366 | gi|313877445|gb|AELG01004290.1| | 65146 | 80064 | 43 | 6 |
| XLOC\_005381 | gi|313877445|gb|AELG01004290.1| | 80936 | 84325 | 8 | 1 |
| XLOC\_005391 | gi|313877446|gb|AELG01004289.1| | 4760 | 32051 | 9 | 1 |
| XLOC\_005436 | gi|313877457|gb|AELG01004278.1| | 580 | 19826 | 18 | 2 |
| XLOC\_005442 XLOC\_005445 | gi|313877458|gb|AELG01004277.1| | 1001 | 10899 | 21 | 1 |
| XLOC\_005448 XLOC\_005444 | gi|313877458|gb|AELG01004277.1| | 23973 | 41014 | 40 | 5 |
| XLOC\_005451 | gi|313877459|gb|AELG01004276.1| | 647 | 10907 | 7 | 2 |
| XLOC\_005489 | gi|313877523|gb|AELG01004231.1| | 7202 | 27339 | 13 | 9 |
| XLOC\_005519 | gi|313877532|gb|AELG01004222.1| | 43995 | 81214 | 17 | 2 |
| XLOC\_005527 | gi|313877536|gb|AELG01004218.1| | 2668 | 13188 | 11 | 1 |
| XLOC\_005563 | gi|313877547|gb|AELG01004207.1| | 73680 | 79866 | 10 | 1 |
| XLOC\_005577 | gi|313877553|gb|AELG01004203.1| | 38063 | 48249 | 17 | 2 |
| XLOC\_005580 | gi|313877553|gb|AELG01004203.1| | 89451 | 137816 | 21 | 1 |
| XLOC\_005594 | gi|313877559|gb|AELG01004197.1| | 25240 | 36699 | 15 | 2 |
| XLOC\_005600 | gi|313877560|gb|AELG01004196.1| | 35850 | 118052 | 12 | 1 |
| XLOC\_005607 | gi|313877562|gb|AELG01004194.1| | 7854 | 18251 | 14 | 3 |
| XLOC\_005618 | gi|313877563|gb|AELG01004193.1| | 5484 | 30037 | 21 | 1 |
| XLOC\_005623 | gi|313877564|gb|AELG01004192.1| | 2669 | 20210 | 16 | 1 |
| XLOC\_005627 | gi|313877567|gb|AELG01004189.1| | 149 | 6165 | 5 | 2 |
| XLOC\_005631 | gi|313877568|gb|AELG01004188.1| | 32029 | 35645 | 17 | 1 |
| XLOC\_005658 | gi|313877575|gb|AELG01004181.1| | 9574 | 16272 | 15 | 1 |
| XLOC\_005739 | gi|313877611|gb|AELG01004151.1| | 67895 | 73837 | 12 | 1 |
| XLOC\_005755 | gi|313877613|gb|AELG01004149.1| | 65571 | 73290 | 9 | 1 |
| XLOC\_005763 XLOC\_005776 | gi|313877613|gb|AELG01004149.1| | 152319 | 156465 | 17 | 1 |
| XLOC\_005765 | gi|313877613|gb|AELG01004149.1| | 164034 | 169145 | 13 | 1 |
| XLOC\_005769 | gi|313877613|gb|AELG01004149.1| | 41974 | 55052 | 19 | 3 |
| XLOC\_005771 XLOC\_005757 | gi|313877613|gb|AELG01004149.1| | 98477 | 115620 | 36 | 2 |
| XLOC\_005791 | gi|313877615|gb|AELG01004147.1| | 23952 | 40232 | 4 | 2 |
| XLOC\_005800 | gi|313877631|gb|AELG01004131.1| | 22167 | 31882 | 20 | 1 |
| XLOC\_005812 | gi|313877632|gb|AELG01004130.1| | 905 | 35289 | 22 | 6 |
| XLOC\_005813 XLOC\_005807 | gi|313877632|gb|AELG01004130.1| | 39582 | 58142 | 26 | 2 |
| XLOC\_005817 XLOC\_005811 | gi|313877632|gb|AELG01004130.1| | 108167 | 171132 | 13 | 1 |
| XLOC\_005820 | gi|313877635|gb|AELG01004127.1| | 48413 | 55064 | 7 | 1 |
| XLOC\_005828 | gi|313877635|gb|AELG01004127.1| | 65535 | 87147 | 16 | 2 |
| XLOC\_005862 | gi|313878097|gb|AELG01004079.1| | 27045 | 44231 | 20 | 3 |
| XLOC\_005870 XLOC\_005868 | gi|313878099|gb|AELG01004078.1| | 19 | 13937 | 17 | 2 |
| XLOC\_005872 | gi|313878100|gb|AELG01004077.1| | 11867 | 20262 | 8 | 1 |
| XLOC\_005881 XLOC\_005879 | gi|313878101|gb|AELG01004076.1| | 13131 | 21111 | 10 | 4 |
| XLOC\_005886 | gi|313878102|gb|AELG01004075.1| | 24983 | 46300 | 6 | 3 |
| XLOC\_005891 | gi|313878103|gb|AELG01004074.1| | 41376 | 48955 | 21 | 4 |
| XLOC\_005894 | gi|313878103|gb|AELG01004074.1| | 13851 | 22250 | 13 | 1 |
| XLOC\_005936 | gi|313878123|gb|AELG01004054.1| | 2865 | 11893 | 30 | 2 |
| XLOC\_005939 XLOC\_005941 | gi|313878124|gb|AELG01004053.1| | 717 | 20100 | 37 | 4 |
| XLOC\_005944 | gi|313878124|gb|AELG01004053.1| | 25981 | 29796 | 15 | 1 |
| XLOC\_005966 XLOC\_005965 XLOC\_005968 XLOC\_005969 | gi|313878131|gb|AELG01004046.1| | 36 | 9978 | 29 | 2 |
| XLOC\_005970 XLOC\_005967 | gi|313878131|gb|AELG01004046.1| | 10496 | 20547 | 12 | 1 |
| XLOC\_005985 | gi|313878134|gb|AELG01004043.1| | 25059 | 36928 | 9 | 2 |
| XLOC\_005988 XLOC\_005989 XLOC\_005993 XLOC\_005994 | gi|313878137|gb|AELG01004041.1| | 13207 | 23558 | 53 | 3 |
| XLOC\_005998 XLOC\_006001 | gi|313878139|gb|AELG01004039.1| | 17810 | 38783 | 15 | 1 |
| XLOC\_006005 XLOC\_006007 | gi|313878140|gb|AELG01004038.1| | 12449 | 19625 | 35 | 2 |
| XLOC\_006009 | gi|313878141|gb|AELG01004037.1| | 1 | 17098 | 23 | 3 |
| XLOC\_006011 XLOC\_006010 | gi|313878141|gb|AELG01004037.1| | 18523 | 27466 | 30 | 3 |
| XLOC\_006013 | gi|313878143|gb|AELG01004035.1| | 1 | 15913 | 19 | 1 |
| XLOC\_006019 | gi|313878144|gb|AELG01004034.1| | 52356 | 133402 | 43 | 22 |
| XLOC\_006020 | gi|313878144|gb|AELG01004034.1| | 148339 | 201062 | 46 | 24 |
| XLOC\_006035 XLOC\_006037 | gi|313878147|gb|AELG01004031.1| | 6536 | 45979 | 51 | 2 |
| XLOC\_006038 | gi|313878148|gb|AELG01004030.1| | 13855 | 19240 | 9 | 1 |
| XLOC\_006054 | gi|313878160|gb|AELG01004018.1| | 9419 | 14891 | 14 | 2 |
| XLOC\_006055 | gi|313878160|gb|AELG01004018.1| | 15080 | 18664 | 3 | 2 |
| XLOC\_006057 | gi|313878161|gb|AELG01004017.1| | 186 | 17682 | 15 | 2 |
| XLOC\_006061 | gi|313878163|gb|AELG01004015.1| | 4665 | 13424 | 11 | 2 |
| XLOC\_006062 | gi|313878163|gb|AELG01004015.1| | 18648 | 26665 | 14 | 2 |
| XLOC\_006067 | gi|313878163|gb|AELG01004015.1| | 26743 | 38029 | 26 | 7 |
| XLOC\_006095 | gi|313878188|gb|AELG01003991.1| | 37408 | 41486 | 11 | 1 |
| XLOC\_006099 | gi|313878188|gb|AELG01003991.1| | 41911 | 48729 | 9 | 3 |
| XLOC\_006104 | gi|313878189|gb|AELG01003990.1| | 33139 | 40139 | 15 | 3 |
| XLOC\_006120 XLOC\_006116 | gi|313878196|gb|AELG01003983.1| | 19788 | 34062 | 32 | 1 |
| XLOC\_006122 XLOC\_006119 | gi|313878196|gb|AELG01003983.1| | 42814 | 64968 | 26 | 8 |
| XLOC\_006138 XLOC\_006141 | gi|313878203|gb|AELG01003976.1| | 25757 | 40115 | 16 | 1 |
| XLOC\_006142 | gi|313878203|gb|AELG01003976.1| | 53641 | 56704 | 9 | 1 |
| XLOC\_006197 XLOC\_006201 | gi|313878227|gb|AELG01003954.1| | 75781 | 89936 | 16 | 2 |
| XLOC\_006200 | gi|313878227|gb|AELG01003954.1| | 77712 | 81529 | 6 | 1 |
| XLOC\_006208 | gi|313878229|gb|AELG01003952.1| | 62272 | 65331 | 9 | 1 |
| XLOC\_006240 | gi|313878242|gb|AELG01003941.1| | 37888 | 88372 | 10 | 3 |
| XLOC\_006249 | gi|313878244|gb|AELG01003939.1| | 79827 | 110649 | 30 | 1 |
| XLOC\_006263 | gi|313878246|gb|AELG01003937.1| | 7218 | 47565 | 6 | 1 |
| XLOC\_006264 | gi|313878247|gb|AELG01003936.1| | 109464 | 143864 | 7 | 2 |
| XLOC\_006267 | gi|313878251|gb|AELG01003932.1| | 18092 | 21161 | 8 | 2 |
| XLOC\_006288 XLOC\_006289 | gi|313878252|gb|AELG01003931.1| | 69426 | 73788 | 10 | 1 |
| XLOC\_006294 | gi|313878256|gb|AELG01003927.1| | 43804 | 57301 | 13 | 2 |
| XLOC\_006296 | gi|313878256|gb|AELG01003927.1| | 59116 | 70998 | 6 | 1 |
| XLOC\_006303 | gi|313878259|gb|AELG01003924.1| | 1886 | 22877 | 25 | 2 |
| XLOC\_006326 | gi|313878261|gb|AELG01003922.1| | 253 | 15536 | 4 | 2 |
| XLOC\_006336 | gi|313878262|gb|AELG01003921.1| | 12 | 43188 | 17 | 4 |
| XLOC\_006343 XLOC\_006342 XLOC\_006344 | gi|313878266|gb|AELG01003918.1| | 1987 | 8800 | 28 | 1 |
| XLOC\_006345 | gi|313878266|gb|AELG01003918.1| | 9222 | 11321 | 2 | 2 |
| XLOC\_006351 XLOC\_006356 XLOC\_006357 | gi|313878267|gb|AELG01003917.1| | 33464 | 56361 | 44 | 7 |
| XLOC\_006353 XLOC\_006346 | gi|313878267|gb|AELG01003917.1| | 129 | 8230 | 28 | 2 |
| XLOC\_006366 XLOC\_006360 | gi|313878268|gb|AELG01003916.1| | 66481 | 83112 | 19 | 2 |
| XLOC\_006370 | gi|313878270|gb|AELG01003914.1| | 8489 | 15345 | 13 | 2 |
| XLOC\_006375 | gi|313878270|gb|AELG01003914.1| | 42046 | 56060 | 13 | 3 |
| XLOC\_006381 | gi|313878271|gb|AELG01003913.1| | 15869 | 28043 | 20 | 2 |
| XLOC\_006394 XLOC\_006404 | gi|313878284|gb|AELG01003900.1| | 117708 | 131683 | 44 | 7 |
| XLOC\_006397 | gi|313878284|gb|AELG01003900.1| | 166169 | 171381 | 8 | 2 |
| XLOC\_006401 XLOC\_006390 | gi|313878284|gb|AELG01003900.1| | 60800 | 83071 | 58 | 6 |
| XLOC\_006402 XLOC\_006392 XLOC\_006393 | gi|313878284|gb|AELG01003900.1| | 103462 | 114490 | 28 | 1 |
| XLOC\_006427 | gi|313878291|gb|AELG01003893.1| | 20607 | 100609 | 16 | 1 |
| XLOC\_006428 XLOC\_006426 | gi|313878291|gb|AELG01003893.1| | 9753 | 20584 | 23 | 4 |
| XLOC\_006434 | gi|313878294|gb|AELG01003890.1| | 32096 | 50775 | 3 | 2 |
| XLOC\_006445 XLOC\_006448 | gi|313878299|gb|AELG01003885.1| | 34671 | 42748 | 25 | 3 |
| XLOC\_006447 | gi|313878299|gb|AELG01003885.1| | 31197 | 34328 | 12 | 1 |
| XLOC\_006449 | gi|313878299|gb|AELG01003885.1| | 45573 | 64552 | 15 | 2 |
| XLOC\_006454 | gi|313878302|gb|AELG01003882.1| | 478 | 6855 | 19 | 5 |
| XLOC\_006457 | gi|313878302|gb|AELG01003882.1| | 14622 | 17121 | 11 | 1 |
| XLOC\_006469 | gi|313878304|gb|AELG01003880.1| | 8813 | 20383 | 30 | 11 |
| XLOC\_006479 | gi|313878305|gb|AELG01003879.1| | 84965 | 89715 | 11 | 1 |
| XLOC\_006484 | gi|313878306|gb|AELG01003878.1| | 12102 | 31528 | 12 | 1 |
| XLOC\_006493 XLOC\_006499 | gi|313878307|gb|AELG01003877.1| | 44648 | 71703 | 45 | 2 |
| XLOC\_006494 | gi|313878307|gb|AELG01003877.1| | 75019 | 81862 | 8 | 2 |
| XLOC\_006508 XLOC\_006512 | gi|313878308|gb|AELG01003876.1| | 59517 | 75311 | 27 | 2 |
| XLOC\_006511 | gi|313878308|gb|AELG01003876.1| | 33320 | 51286 | 13 | 1 |
| XLOC\_006517 XLOC\_006515 | gi|313878309|gb|AELG01003875.1| | 6637 | 25196 | 23 | 1 |
| XLOC\_006523 | gi|313878310|gb|AELG01003874.1| | 39444 | 48854 | 12 | 3 |
| XLOC\_006532 XLOC\_006543 | gi|313878310|gb|AELG01003874.1| | 127355 | 136079 | 14 | 1 |
| XLOC\_006544 XLOC\_006533 | gi|313878310|gb|AELG01003874.1| | 136782 | 145494 | 53 | 1 |
| XLOC\_006545 XLOC\_006534 | gi|313878310|gb|AELG01003874.1| | 146023 | 169903 | 34 | 7 |
| XLOC\_006551 XLOC\_006555 | gi|313878311|gb|AELG01003873.1| | 4470 | 14748 | 29 | 1 |
| XLOC\_006564 | gi|313878313|gb|AELG01003871.1| | 2447 | 11763 | 9 | 2 |
| XLOC\_006567 | gi|313878319|gb|AELG01003866.1| | 91667 | 106157 | 6 | 1 |
| XLOC\_006581 XLOC\_006579 | gi|313878320|gb|AELG01003865.1| | 7558 | 18644 | 31 | 1 |
| XLOC\_006582 XLOC\_006586 | gi|313878320|gb|AELG01003865.1| | 63579 | 78719 | 10 | 3 |
| XLOC\_006610 | gi|313878325|gb|AELG01003860.1| | 3103 | 51071 | 24 | 10 |
| XLOC\_006612 | gi|313878326|gb|AELG01003859.1| | 926 | 25714 | 13 | 1 |
| XLOC\_006616 | gi|313878327|gb|AELG01003858.1| | 39688 | 73668 | 9 | 1 |
| XLOC\_006617 | gi|313878327|gb|AELG01003858.1| | 75674 | 114139 | 14 | 3 |
| XLOC\_006628 XLOC\_006630 | gi|313878328|gb|AELG01003857.1| | 53061 | 65377 | 38 | 1 |
| XLOC\_006631 | gi|313878328|gb|AELG01003857.1| | 67381 | 77613 | 19 | 1 |
| XLOC\_006644 XLOC\_006642 | gi|313878330|gb|AELG01003855.1| | 55 | 6253 | 16 | 5 |
| XLOC\_006645 | gi|313878330|gb|AELG01003855.1| | 9084 | 41008 | 19 | 3 |
| XLOC\_006664 | gi|313878335|gb|AELG01003850.1| | 29510 | 36551 | 10 | 2 |
| XLOC\_006670 | gi|313878336|gb|AELG01003849.1| | 1821 | 12660 | 24 | 6 |
| XLOC\_006675 | gi|313878336|gb|AELG01003849.1| | 40691 | 53530 | 9 | 2 |
| XLOC\_006689 | gi|313878344|gb|AELG01003842.1| | 556 | 9154 | 12 | 1 |
| XLOC\_006702 | gi|313878364|gb|AELG01003822.1| | 57385 | 123762 | 28 | 6 |
| XLOC\_006709 | gi|313878368|gb|AELG01003818.1| | 40984 | 52366 | 20 | 2 |
| XLOC\_006710 XLOC\_006713 | gi|313878368|gb|AELG01003818.1| | 57446 | 59854 | 14 | 1 |
| XLOC\_006712 | gi|313878368|gb|AELG01003818.1| | 52542 | 56773 | 6 | 2 |
| XLOC\_006733 | gi|313878374|gb|AELG01003812.1| | 100398 | 107643 | 4 | 2 |
| XLOC\_006742 | gi|313878390|gb|AELG01003797.1| | 22441 | 47918 | 12 | 3 |
| XLOC\_006760 | gi|313878390|gb|AELG01003797.1| | 140814 | 163878 | 7 | 1 |
| XLOC\_006789 XLOC\_006788 XLOC\_006792 | gi|313878401|gb|AELG01003786.1| | 74876 | 85524 | 22 | 3 |
| XLOC\_006791 XLOC\_006787 | gi|313878401|gb|AELG01003786.1| | 13103 | 52575 | 16 | 5 |
| XLOC\_006797 | gi|313878402|gb|AELG01003785.1| | 90958 | 110273 | 10 | 2 |
| XLOC\_006806 | gi|313878403|gb|AELG01003784.1| | 7657 | 26534 | 7 | 5 |
| XLOC\_006827 | gi|313878409|gb|AELG01003779.1| | 654 | 23586 | 25 | 1 |
| XLOC\_006828 | gi|313878409|gb|AELG01003779.1| | 23720 | 67745 | 15 | 6 |
| XLOC\_006834 | gi|313878410|gb|AELG01003778.1| | 23699 | 28305 | 19 | 1 |
| XLOC\_006839 XLOC\_006836 | gi|313878410|gb|AELG01003778.1| | 33224 | 77890 | 38 | 3 |
| XLOC\_006858 XLOC\_006864 | gi|313878424|gb|AELG01003764.1| | 8622 | 12765 | 5 | 1 |
| XLOC\_006859 | gi|313878424|gb|AELG01003764.1| | 12971 | 16435 | 17 | 1 |
| XLOC\_006861 XLOC\_006856 | gi|313878424|gb|AELG01003764.1| | 27125 | 53701 | 25 | 3 |
| XLOC\_006869 | gi|313878425|gb|AELG01003763.1| | 8606 | 17303 | 16 | 4 |
| XLOC\_006875 XLOC\_006870 | gi|313878425|gb|AELG01003763.1| | 21380 | 72469 | 58 | 2 |
| XLOC\_006881 | gi|313878426|gb|AELG01003762.1| | 9220 | 39267 | 6 | 1 |
| XLOC\_006882 XLOC\_006890 | gi|313878426|gb|AELG01003762.1| | 41695 | 50409 | 30 | 3 |
| XLOC\_006885 | gi|313878426|gb|AELG01003762.1| | 72735 | 82203 | 15 | 3 |
| XLOC\_006886 | gi|313878426|gb|AELG01003762.1| | 86854 | 101365 | 22 | 4 |
| XLOC\_006891 | gi|313878426|gb|AELG01003762.1| | 105532 | 109424 | 10 | 1 |
| XLOC\_006908 | gi|313878435|gb|AELG01003753.1| | 1029 | 24422 | 26 | 3 |
| XLOC\_006921 XLOC\_006917 | gi|313878436|gb|AELG01003752.1| | 37865 | 43362 | 24 | 1 |
| XLOC\_006929 | gi|313878439|gb|AELG01003750.1| | 32633 | 43198 | 8 | 1 |
| XLOC\_006932 | gi|313878441|gb|AELG01003748.1| | 7061 | 15798 | 15 | 1 |
| XLOC\_006933 | gi|313878441|gb|AELG01003748.1| | 16810 | 25105 | 7 | 5 |
| XLOC\_006934 | gi|313878442|gb|AELG01003747.1| | 43913 | 56723 | 24 | 6 |
| XLOC\_006939 | gi|313878443|gb|AELG01003746.1| | 16425 | 57477 | 6 | 1 |
| XLOC\_006954 | gi|313878446|gb|AELG01003743.1| | 8900 | 15138 | 18 | 1 |
| XLOC\_006958 XLOC\_006961 | gi|313878449|gb|AELG01003740.1| | 283 | 9925 | 14 | 1 |
| XLOC\_006959 | gi|313878449|gb|AELG01003740.1| | 16820 | 37620 | 24 | 2 |
| XLOC\_006993 | gi|313878463|gb|AELG01003727.1| | 20777 | 46245 | 9 | 1 |
| XLOC\_007001 | gi|313878470|gb|AELG01003720.1| | 60580 | 125068 | 55 | 18 |
| XLOC\_007010 | gi|313878471|gb|AELG01003719.1| | 14892 | 41975 | 9 | 1 |
| XLOC\_007019 | gi|313878472|gb|AELG01003718.1| | 70417 | 86576 | 9 | 1 |
| XLOC\_007054 | gi|313878486|gb|AELG01003704.1| | 209 | 117736 | 7 | 2 |
| XLOC\_007059 | gi|313878487|gb|AELG01003703.1| | 61462 | 73947 | 10 | 1 |
| XLOC\_007068 | gi|313878488|gb|AELG01003702.1| | 3839 | 38116 | 17 | 2 |
| XLOC\_007077 | gi|313878489|gb|AELG01003701.1| | 80168 | 100520 | 31 | 16 |
| XLOC\_007084 XLOC\_007089 | gi|313878489|gb|AELG01003701.1| | 101125 | 110313 | 12 | 6 |
| XLOC\_007093 | gi|313878494|gb|AELG01003696.1| | 11223 | 38567 | 8 | 1 |
| XLOC\_007102 | gi|313878495|gb|AELG01003695.1| | 61281 | 67508 | 3 | 2 |
| XLOC\_007103 | gi|313878495|gb|AELG01003695.1| | 72576 | 92994 | 16 | 6 |
| XLOC\_007106 | gi|313878495|gb|AELG01003695.1| | 113086 | 116365 | 10 | 1 |
| XLOC\_007113 XLOC\_007105 | gi|313878495|gb|AELG01003695.1| | 96767 | 112917 | 35 | 8 |
| XLOC\_007120 | gi|313878496|gb|AELG01003694.1| | 15251 | 22259 | 10 | 1 |
| XLOC\_007129 | gi|313878499|gb|AELG01003691.1| | 1 | 9098 | 6 | 1 |
| XLOC\_007149 | gi|313878504|gb|AELG01003686.1| | 49069 | 202353 | 5 | 1 |
| XLOC\_007150 XLOC\_007145 | gi|313878504|gb|AELG01003686.1| | 204147 | 224195 | 62 | 18 |
| XLOC\_007151 | gi|313878504|gb|AELG01003686.1| | 225057 | 240139 | 18 | 2 |
| XLOC\_007176 | gi|313878512|gb|AELG01003678.1| | 80323 | 87110 | 25 | 2 |
| XLOC\_007181 | gi|313878514|gb|AELG01003676.1| | 5563 | 12531 | 17 | 1 |
| XLOC\_007185 XLOC\_007188 | gi|313878515|gb|AELG01003675.1| | 11694 | 17775 | 16 | 1 |
| XLOC\_007186 XLOC\_007189 | gi|313878515|gb|AELG01003675.1| | 20081 | 27247 | 8 | 2 |
| XLOC\_007205 | gi|313878518|gb|AELG01003673.1| | 53011 | 60830 | 16 | 4 |
| XLOC\_007211 XLOC\_007202 | gi|313878518|gb|AELG01003673.1| | 26119 | 32896 | 24 | 5 |
| XLOC\_007213 XLOC\_007208 | gi|313878518|gb|AELG01003673.1| | 68863 | 76893 | 37 | 2 |
| XLOC\_007227 | gi|313878520|gb|AELG01003671.1| | 24928 | 30197 | 8 | 2 |
| XLOC\_007232 XLOC\_007234 | gi|313878522|gb|AELG01003669.1| | 5681 | 19867 | 18 | 3 |
| XLOC\_007246 | gi|313878527|gb|AELG01003664.1| | 176775 | 189311 | 29 | 6 |
| XLOC\_007249 | gi|313878527|gb|AELG01003664.1| | 149952 | 153054 | 8 | 1 |
| XLOC\_007252 | gi|313878527|gb|AELG01003664.1| | 161102 | 166773 | 11 | 1 |
| XLOC\_007269 | gi|313878560|gb|AELG01003632.1| | 2 | 5359 | 15 | 1 |
| XLOC\_007271 | gi|313878561|gb|AELG01003631.1| | 5725 | 9465 | 15 | 1 |
| XLOC\_007286 | gi|313878566|gb|AELG01003626.1| | 836 | 6793 | 19 | 1 |
| XLOC\_007325 | gi|313878630|gb|AELG01003563.1| | 6542 | 13750 | 5 | 2 |
| XLOC\_007339 | gi|313878635|gb|AELG01003558.1| | 11489 | 22090 | 5 | 1 |
| XLOC\_007347 | gi|313878638|gb|AELG01003556.1| | 1906 | 12612 | 10 | 1 |
| XLOC\_007356 | gi|313878638|gb|AELG01003556.1| | 91350 | 104346 | 20 | 1 |
| XLOC\_007358 | gi|313878638|gb|AELG01003556.1| | 107853 | 129284 | 9 | 5 |
| XLOC\_007378 | gi|313878648|gb|AELG01003546.1| | 2165 | 11414 | 10 | 1 |
| XLOC\_007383 XLOC\_007390 | gi|313878649|gb|AELG01003545.1| | 25758 | 31653 | 13 | 1 |
| XLOC\_007416 | gi|313878668|gb|AELG01003526.1| | 142464 | 152766 | 9 | 3 |
| XLOC\_007434 | gi|313878671|gb|AELG01003523.1| | 61986 | 81960 | 32 | 6 |
| XLOC\_007438 | gi|313878671|gb|AELG01003523.1| | 17736 | 29656 | 27 | 1 |
| XLOC\_007440 | gi|313878671|gb|AELG01003523.1| | 66335 | 73425 | 13 | 1 |
| XLOC\_007476 | gi|313878693|gb|AELG01003502.1| | 6146 | 18308 | 17 | 4 |
| XLOC\_007490 XLOC\_007488 XLOC\_007487 XLOC\_007484 | gi|313878695|gb|AELG01003500.1| | 36 | 9036 | 25 | 1 |
| XLOC\_007499 XLOC\_007506 | gi|313878699|gb|AELG01003496.1| | 8586 | 18662 | 23 | 1 |
| XLOC\_007511 XLOC\_007504 | gi|313878699|gb|AELG01003496.1| | 90462 | 97535 | 19 | 2 |
| XLOC\_007520 | gi|313878701|gb|AELG01003494.1| | 24713 | 75666 | 26 | 16 |
| XLOC\_007532 | gi|313878739|gb|AELG01003457.1| | 40 | 10250 | 15 | 5 |
| XLOC\_007533 | gi|313878740|gb|AELG01003456.1| | 19420 | 31133 | 6 | 1 |
| XLOC\_007547 | gi|313878749|gb|AELG01003447.1| | 86437 | 93765 | 18 | 2 |
| XLOC\_007550 | gi|313878749|gb|AELG01003447.1| | 121156 | 126403 | 32 | 3 |
| XLOC\_007551 XLOC\_007543 XLOC\_007554 | gi|313878749|gb|AELG01003447.1| | 46 | 47490 | 50 | 5 |
| XLOC\_007558 | gi|313878750|gb|AELG01003446.1| | 157683 | 162964 | 7 | 3 |
| XLOC\_007568 | gi|313878750|gb|AELG01003446.1| | 219748 | 224451 | 10 | 1 |
| XLOC\_007604 | gi|313878768|gb|AELG01003429.1| | 14440 | 53162 | 6 | 4 |
| XLOC\_007608 | gi|313878769|gb|AELG01003428.1| | 1821 | 8797 | 8 | 1 |
| XLOC\_007616 XLOC\_007614 XLOC\_007613 | gi|313878772|gb|AELG01003425.1| | 19 | 20165 | 60 | 8 |
| XLOC\_007626 XLOC\_007632 | gi|313878776|gb|AELG01003421.1| | 20251 | 29393 | 36 | 2 |
| XLOC\_007627 | gi|313878776|gb|AELG01003421.1| | 34044 | 39196 | 12 | 1 |
| XLOC\_007630 | gi|313878776|gb|AELG01003421.1| | 62886 | 67593 | 14 | 1 |
| XLOC\_007645 XLOC\_007640 | gi|313878778|gb|AELG01003419.1| | 142676 | 152183 | 24 | 1 |
| XLOC\_007652 XLOC\_007654 | gi|313878782|gb|AELG01003415.1| | 3312 | 47170 | 23 | 1 |
| XLOC\_007659 | gi|313878784|gb|AELG01003413.1| | 34847 | 46023 | 8 | 2 |
| XLOC\_007670 | gi|313878788|gb|AELG01003409.1| | 12354 | 24617 | 5 | 2 |
| XLOC\_007673 | gi|313878789|gb|AELG01003408.1| | 49479 | 57440 | 5 | 2 |
| XLOC\_007681 | gi|313878794|gb|AELG01003403.1| | 52009 | 129226 | 6 | 2 |
| XLOC\_007682 | gi|313878794|gb|AELG01003403.1| | 129299 | 144018 | 36 | 2 |
| XLOC\_007691 | gi|313878806|gb|AELG01003391.1| | 16853 | 37548 | 22 | 1 |
| XLOC\_007703 XLOC\_007699 | gi|313878812|gb|AELG01003385.1| | 19366 | 67896 | 17 | 2 |
| XLOC\_007712 XLOC\_007710 | gi|313878832|gb|AELG01003365.1| | 22609 | 30493 | 25 | 1 |
| XLOC\_007721 | gi|313878833|gb|AELG01003364.1| | 38142 | 41892 | 8 | 1 |
| XLOC\_007746 | gi|313878834|gb|AELG01003363.1| | 27228 | 31273 | 8 | 1 |
| XLOC\_007753 | gi|313878835|gb|AELG01003362.1| | 9504 | 28383 | 15 | 1 |
| XLOC\_007757 | gi|313878837|gb|AELG01003360.1| | 9639 | 52670 | 46 | 3 |
| XLOC\_007769 | gi|313878840|gb|AELG01003357.1| | 29870 | 36193 | 17 | 1 |
| XLOC\_007771 | gi|313878840|gb|AELG01003357.1| | 67427 | 85420 | 18 | 1 |
| XLOC\_007772 XLOC\_007768 | gi|313878840|gb|AELG01003357.1| | 10735 | 22087 | 22 | 4 |
| XLOC\_007773 | gi|313878840|gb|AELG01003357.1| | 22466 | 27954 | 12 | 1 |
| XLOC\_007774 | gi|313878840|gb|AELG01003357.1| | 36553 | 41252 | 4 | 2 |
| XLOC\_007780 | gi|313878841|gb|AELG01003356.1| | 5555 | 29029 | 8 | 1 |
| XLOC\_007784 | gi|313878843|gb|AELG01003354.1| | 4350 | 9842 | 18 | 1 |
| XLOC\_007789 | gi|313878845|gb|AELG01003352.1| | 1327 | 5194 | 4 | 1 |
| XLOC\_007795 | gi|313878847|gb|AELG01003350.1| | 8726 | 16420 | 10 | 1 |
| XLOC\_007804 | gi|313878851|gb|AELG01003346.1| | 20 | 3570 | 8 | 1 |
| XLOC\_007810 | gi|313878853|gb|AELG01003344.1| | 58 | 4820 | 5 | 1 |
| XLOC\_007811 | gi|313878854|gb|AELG01003343.1| | 2644 | 23728 | 36 | 10 |
| XLOC\_007819 | gi|313878857|gb|AELG01003340.1| | 6823 | 13266 | 11 | 2 |
| XLOC\_007875 | gi|313878898|gb|AELG01003300.1| | 30970 | 46549 | 9 | 2 |
| XLOC\_007919 | gi|313878906|gb|AELG01003292.1| | 10393 | 23721 | 10 | 1 |
| XLOC\_007924 | gi|313878907|gb|AELG01003291.1| | 3675 | 22563 | 22 | 5 |
| XLOC\_007947 | gi|313878911|gb|AELG01003287.1| | 51685 | 58493 | 9 | 1 |
| XLOC\_007948 XLOC\_007950 | gi|313878911|gb|AELG01003287.1| | 70031 | 87506 | 15 | 1 |
| XLOC\_007967 | gi|313878919|gb|AELG01003280.1| | 7097 | 24551 | 9 | 8 |
| XLOC\_007968 XLOC\_007964 | gi|313878919|gb|AELG01003280.1| | 28527 | 42397 | 40 | 9 |
| XLOC\_007971 | gi|313878919|gb|AELG01003280.1| | 59594 | 62368 | 8 | 1 |
| XLOC\_008016 XLOC\_008018 | gi|313879003|gb|AELG01003198.1| | 13504 | 24775 | 34 | 3 |
| XLOC\_008019 | gi|313879003|gb|AELG01003198.1| | 29909 | 35340 | 13 | 1 |
| XLOC\_008022 | gi|313879004|gb|AELG01003197.1| | 61198 | 65874 | 4 | 2 |
| XLOC\_008031 | gi|313879005|gb|AELG01003196.1| | 35526 | 43659 | 10 | 1 |
| XLOC\_008040 | gi|313879007|gb|AELG01003194.1| | 35689 | 63500 | 9 | 1 |
| XLOC\_008051 XLOC\_008057 XLOC\_008058 | gi|313879012|gb|AELG01003189.1| | 6650 | 19318 | 54 | 1 |
| XLOC\_008052 | gi|313879012|gb|AELG01003189.1| | 28382 | 34726 | 34 | 1 |
| XLOC\_008053 XLOC\_008061 | gi|313879012|gb|AELG01003189.1| | 35270 | 73274 | 14 | 6 |
| XLOC\_008059 | gi|313879012|gb|AELG01003189.1| | 19606 | 24141 | 3 | 2 |
| XLOC\_008068 | gi|313879013|gb|AELG01003188.1| | 13 | 6289 | 10 | 1 |
| XLOC\_008074 | gi|313879014|gb|AELG01003187.1| | 59171 | 61261 | 4 | 1 |
| XLOC\_008078 | gi|313879014|gb|AELG01003187.1| | 125719 | 129459 | 16 | 1 |
| XLOC\_008081 XLOC\_008070 | gi|313879014|gb|AELG01003187.1| | 8226 | 22587 | 37 | 4 |
| XLOC\_008083 XLOC\_008072 | gi|313879014|gb|AELG01003187.1| | 41677 | 56149 | 36 | 7 |
| XLOC\_008088 | gi|313879015|gb|AELG01003186.1| | 3568 | 32334 | 23 | 2 |
| XLOC\_008094 XLOC\_008097 | gi|313879016|gb|AELG01003185.1| | 53146 | 75829 | 45 | 3 |
| XLOC\_008095 | gi|313879016|gb|AELG01003185.1| | 58605 | 62831 | 6 | 1 |
| XLOC\_008100 | gi|313879017|gb|AELG01003184.1| | 24346 | 38837 | 16 | 3 |
| XLOC\_008111 XLOC\_008120 | gi|313879024|gb|AELG01003177.1| | 13597 | 18735 | 25 | 1 |
| XLOC\_008121 | gi|313879024|gb|AELG01003177.1| | 20895 | 34264 | 25 | 1 |
| XLOC\_008124 XLOC\_008128 | gi|313879025|gb|AELG01003176.1| | 41624 | 54092 | 16 | 1 |
| XLOC\_008125 XLOC\_008129 | gi|313879025|gb|AELG01003176.1| | 54629 | 82648 | 44 | 2 |
| XLOC\_008148 XLOC\_008147 XLOC\_008136 | gi|313879027|gb|AELG01003174.1| | 10337 | 29362 | 37 | 2 |
| XLOC\_008155 XLOC\_008145 | gi|313879027|gb|AELG01003174.1| | 91256 | 97903 | 41 | 2 |
| XLOC\_008166 | gi|313879030|gb|AELG01003171.1| | 75563 | 88526 | 10 | 1 |
| XLOC\_008169 XLOC\_008164 | gi|313879030|gb|AELG01003171.1| | 38272 | 63400 | 16 | 1 |
| XLOC\_008186 | gi|313879038|gb|AELG01003163.1| | 36559 | 82374 | 22 | 2 |
| XLOC\_008205 | gi|313879041|gb|AELG01003160.1| | 33 | 7699 | 13 | 3 |
| XLOC\_008219 XLOC\_008210 | gi|313879042|gb|AELG01003159.1| | 37761 | 79701 | 66 | 6 |
| XLOC\_008237 | gi|313879045|gb|AELG01003156.1| | 7902 | 15163 | 21 | 4 |
| XLOC\_008268 | gi|313879056|gb|AELG01003146.1| | 5629 | 49869 | 22 | 2 |
| XLOC\_008299 | gi|313879073|gb|AELG01003129.1| | 4802 | 38381 | 16 | 1 |
| XLOC\_008302 | gi|313879076|gb|AELG01003126.1| | 1140 | 6519 | 5 | 3 |
| XLOC\_008331 | gi|313879115|gb|AELG01003087.1| | 8318 | 16781 | 8 | 1 |
| XLOC\_008334 XLOC\_008338 | gi|313879115|gb|AELG01003087.1| | 63050 | 69445 | 13 | 1 |
| XLOC\_008358 XLOC\_008356 | gi|313879126|gb|AELG01003076.1| | 134567 | 140620 | 10 | 1 |
| XLOC\_008361 | gi|313879132|gb|AELG01003070.1| | 47097 | 50743 | 11 | 1 |
| XLOC\_008362 XLOC\_008366 | gi|313879132|gb|AELG01003070.1| | 51325 | 57635 | 17 | 1 |
| XLOC\_008403 | gi|313879147|gb|AELG01003055.1| | 28373 | 35712 | 16 | 3 |
| XLOC\_008406 | gi|313879147|gb|AELG01003055.1| | 44540 | 48316 | 11 | 1 |
| XLOC\_008412 XLOC\_008409 | gi|313879148|gb|AELG01003054.1| | 15309 | 25239 | 22 | 1 |
| XLOC\_008432 | gi|313879157|gb|AELG01003045.1| | 13849 | 18004 | 12 | 2 |
| XLOC\_008435 | gi|313879157|gb|AELG01003045.1| | 42890 | 58063 | 17 | 1 |
| XLOC\_008450 XLOC\_008452 | gi|313879163|gb|AELG01003039.1| | 66410 | 74101 | 19 | 1 |
| XLOC\_008451 | gi|313879163|gb|AELG01003039.1| | 5420 | 44862 | 19 | 3 |
| XLOC\_008469 XLOC\_008465 | gi|313879166|gb|AELG01003037.1| | 3924 | 14617 | 17 | 5 |
| XLOC\_008477 | gi|313879167|gb|AELG01003036.1| | 50 | 9715 | 10 | 8 |
| XLOC\_008478 | gi|313879167|gb|AELG01003036.1| | 11413 | 51500 | 20 | 5 |
| XLOC\_008513 | gi|313879176|gb|AELG01003027.1| | 21687 | 46153 | 6 | 1 |
| XLOC\_008530 | gi|313879183|gb|AELG01003020.1| | 134129 | 191932 | 11 | 1 |
| XLOC\_008569 XLOC\_008574 | gi|313879212|gb|AELG01002991.1| | 24112 | 57992 | 58 | 6 |
| XLOC\_008571 | gi|313879212|gb|AELG01002991.1| | 59639 | 64484 | 6 | 3 |
| XLOC\_008588 | gi|313879215|gb|AELG01002988.1| | 105139 | 113780 | 9 | 2 |
| XLOC\_008591 | gi|313879215|gb|AELG01002988.1| | 36835 | 75721 | 34 | 3 |
| XLOC\_008596 | gi|313879215|gb|AELG01002988.1| | 122244 | 127698 | 15 | 1 |
| XLOC\_008600 | gi|313879217|gb|AELG01002986.1| | 21901 | 34676 | 8 | 2 |
| XLOC\_008604 XLOC\_008607 | gi|313879218|gb|AELG01002985.1| | 13306 | 163479 | 47 | 4 |
| XLOC\_008609 | gi|313879219|gb|AELG01002984.1| | 5304 | 21589 | 27 | 5 |
| XLOC\_008614 XLOC\_008619 | gi|313879221|gb|AELG01002982.1| | 69426 | 125503 | 25 | 1 |
| XLOC\_008618 | gi|313879221|gb|AELG01002982.1| | 65198 | 69209 | 14 | 2 |
| XLOC\_008620 | gi|313879222|gb|AELG01002981.1| | 31837 | 57533 | 14 | 6 |
| XLOC\_008640 XLOC\_008641 | gi|313879243|gb|AELG01002961.1| | 8 | 11329 | 17 | 6 |
| XLOC\_008643 | gi|313879244|gb|AELG01002960.1| | 13604 | 21888 | 20 | 1 |
| XLOC\_008671 | gi|313879265|gb|AELG01002939.1| | 24370 | 29332 | 13 | 1 |
| XLOC\_008716 | gi|313879291|gb|AELG01002914.1| | 15219 | 30340 | 17 | 1 |
| XLOC\_008724 | gi|313879299|gb|AELG01002906.1| | 5 | 13692 | 12 | 2 |
| XLOC\_008728 | gi|313879301|gb|AELG01002904.1| | 1152 | 25379 | 11 | 3 |
| XLOC\_008778 | gi|313879362|gb|AELG01002845.1| | 355 | 8974 | 9 | 2 |
| XLOC\_008781 XLOC\_008783 | gi|313879365|gb|AELG01002842.1| | 78979 | 84043 | 21 | 1 |
| XLOC\_008782 | gi|313879365|gb|AELG01002842.1| | 55622 | 75339 | 10 | 5 |
| XLOC\_008786 | gi|313879367|gb|AELG01002840.1| | 5697 | 56983 | 8 | 3 |
| XLOC\_008808 | gi|313879377|gb|AELG01002830.1| | 30649 | 105029 | 28 | 3 |
| XLOC\_008848 | gi|313879425|gb|AELG01002784.1| | 9656 | 17266 | 10 | 1 |
| XLOC\_008853 | gi|313879427|gb|AELG01002782.1| | 9289 | 18737 | 30 | 8 |
| XLOC\_008863 | gi|313879427|gb|AELG01002782.1| | 42614 | 55949 | 30 | 5 |
| XLOC\_008865 XLOC\_008856 | gi|313879427|gb|AELG01002782.1| | 63466 | 77686 | 27 | 2 |
| XLOC\_008882 | gi|313879431|gb|AELG01002778.1| | 1070 | 7335 | 10 | 1 |
| XLOC\_008885 | gi|313879432|gb|AELG01002777.1| | 7414 | 11847 | 16 | 1 |
| XLOC\_008893 | gi|313879436|gb|AELG01002773.1| | 9016 | 15495 | 19 | 1 |
| XLOC\_008910 XLOC\_008905 | gi|313879438|gb|AELG01002771.1| | 5343 | 13817 | 14 | 1 |
| XLOC\_008920 | gi|313879440|gb|AELG01002769.1| | 29409 | 38425 | 15 | 1 |
| XLOC\_008925 XLOC\_008921 | gi|313879440|gb|AELG01002769.1| | 7 | 26306 | 16 | 1 |
| XLOC\_008934 XLOC\_008941 | gi|313879443|gb|AELG01002766.1| | 52948 | 101172 | 52 | 10 |
| XLOC\_008938 | gi|313879443|gb|AELG01002766.1| | 9 | 35237 | 12 | 2 |
| XLOC\_008952 | gi|313879447|gb|AELG01002762.1| | 9731 | 16944 | 17 | 1 |
| XLOC\_008957 | gi|313879447|gb|AELG01002762.1| | 34511 | 44197 | 12 | 2 |
| XLOC\_008972 | gi|313879450|gb|AELG01002759.1| | 116009 | 124731 | 22 | 1 |
| XLOC\_008976 | gi|313879450|gb|AELG01002759.1| | 250542 | 256256 | 6 | 1 |
| XLOC\_008978 | gi|313879450|gb|AELG01002759.1| | 163424 | 174138 | 18 | 1 |
| XLOC\_008979 | gi|313879450|gb|AELG01002759.1| | 176366 | 186803 | 17 | 1 |
| XLOC\_009002 | gi|313879453|gb|AELG01002757.1| | 7978 | 14279 | 14 | 1 |
| XLOC\_009004 | gi|313879453|gb|AELG01002757.1| | 14331 | 34884 | 19 | 3 |
| XLOC\_009005 | gi|313879453|gb|AELG01002757.1| | 35082 | 39500 | 11 | 1 |
| XLOC\_009011 | gi|313879454|gb|AELG01002756.1| | 3597 | 79123 | 9 | 2 |
| XLOC\_009015 | gi|313879456|gb|AELG01002754.1| | 14514 | 20580 | 14 | 4 |
| XLOC\_009026 XLOC\_009025 | gi|313879468|gb|AELG01002742.1| | 469 | 10368 | 17 | 2 |
| XLOC\_009044 | gi|313879500|gb|AELG01002711.1| | 1 | 13584 | 28 | 2 |
| XLOC\_009056 | gi|313879504|gb|AELG01002707.1| | 65 | 5070 | 11 | 3 |
| XLOC\_009061 XLOC\_009060 | gi|313879505|gb|AELG01002706.1| | 22002 | 30069 | 31 | 3 |
| XLOC\_009065 | gi|313879508|gb|AELG01002703.1| | 300 | 21306 | 20 | 1 |
| XLOC\_009067 | gi|313879509|gb|AELG01002702.1| | 8198 | 14127 | 23 | 3 |
| XLOC\_009070 | gi|313879509|gb|AELG01002702.1| | 34566 | 43507 | 6 | 3 |
| XLOC\_009072 | gi|313879510|gb|AELG01002701.1| | 14435 | 35798 | 13 | 2 |
| XLOC\_009074 | gi|313879510|gb|AELG01002701.1| | 854 | 4192 | 2 | 2 |
| XLOC\_009077 | gi|313879510|gb|AELG01002701.1| | 61869 | 68074 | 10 | 1 |
| XLOC\_009114 | gi|313879526|gb|AELG01002686.1| | 3997 | 6709 | 6 | 1 |
| XLOC\_009126 | gi|313879528|gb|AELG01002684.1| | 16285 | 20815 | 11 | 1 |
| XLOC\_009128 | gi|313879529|gb|AELG01002683.1| | 11018 | 19587 | 24 | 4 |
| XLOC\_009135 | gi|313879529|gb|AELG01002683.1| | 82035 | 87478 | 4 | 1 |
| XLOC\_009139 | gi|313879529|gb|AELG01002683.1| | 50343 | 53989 | 8 | 1 |
| XLOC\_009145 XLOC\_009136 | gi|313879529|gb|AELG01002683.1| | 91621 | 99876 | 31 | 1 |
| XLOC\_009151 | gi|313879531|gb|AELG01002681.1| | 66062 | 71777 | 10 | 3 |
| XLOC\_009165 | gi|313879532|gb|AELG01002680.1| | 9915 | 17429 | 5 | 1 |
| XLOC\_009228 XLOC\_009230 XLOC\_009231 | gi|313879543|gb|AELG01002669.1| | 339 | 18998 | 53 | 5 |
| XLOC\_009236 | gi|313879544|gb|AELG01002668.1| | 25172 | 40425 | 31 | 5 |
| XLOC\_009255 XLOC\_009257 | gi|313879545|gb|AELG01002667.1| | 30539 | 44485 | 61 | 3 |
| XLOC\_009256 | gi|313879545|gb|AELG01002667.1| | 5177 | 15112 | 9 | 1 |
| XLOC\_009295 XLOC\_009298 | gi|313879558|gb|AELG01002655.1| | 29413 | 40300 | 11 | 2 |
| XLOC\_009296 | gi|313879558|gb|AELG01002655.1| | 625 | 14998 | 8 | 1 |
| XLOC\_009307 XLOC\_009305 XLOC\_009308 | gi|313879559|gb|AELG01002654.1| | 16472 | 62951 | 70 | 6 |
| XLOC\_009320 XLOC\_009322 | gi|313879560|gb|AELG01002653.1| | 26848 | 30911 | 12 | 1 |
| XLOC\_009349 | gi|313879572|gb|AELG01002642.1| | 12646 | 70813 | 24 | 16 |
| XLOC\_009353 | gi|313879572|gb|AELG01002642.1| | 94921 | 107388 | 17 | 2 |
| XLOC\_009381 | gi|313879575|gb|AELG01002639.1| | 28846 | 34053 | 11 | 1 |
| XLOC\_009401 | gi|313879577|gb|AELG01002637.1| | 14878 | 31299 | 22 | 1 |
| XLOC\_009402 | gi|313879577|gb|AELG01002637.1| | 39756 | 45482 | 18 | 1 |
| XLOC\_009408 | gi|313879578|gb|AELG01002636.1| | 46786 | 52916 | 8 | 1 |
| XLOC\_009424 XLOC\_009419 | gi|313879583|gb|AELG01002631.1| | 20764 | 32979 | 17 | 1 |
| XLOC\_009425 | gi|313879583|gb|AELG01002631.1| | 111174 | 143041 | 15 | 6 |
| XLOC\_009434 | gi|313879584|gb|AELG01002630.1| | 41836 | 86803 | 7 | 2 |
| XLOC\_009447 | gi|313879584|gb|AELG01002630.1| | 310728 | 333381 | 17 | 7 |
| XLOC\_009458 | gi|313879590|gb|AELG01002624.1| | 61514 | 87803 | 19 | 1 |
| XLOC\_009459 XLOC\_009466 | gi|313879590|gb|AELG01002624.1| | 89945 | 97987 | 23 | 1 |
| XLOC\_009461 | gi|313879590|gb|AELG01002624.1| | 116763 | 119090 | 3 | 2 |
| XLOC\_009470 | gi|313879590|gb|AELG01002624.1| | 130212 | 134626 | 4 | 1 |
| XLOC\_009482 | gi|313879593|gb|AELG01002622.1| | 33494 | 75214 | 4 | 1 |
| XLOC\_009500 XLOC\_009505 | gi|313879596|gb|AELG01002619.1| | 122357 | 126895 | 18 | 1 |
| XLOC\_009511 XLOC\_009521 XLOC\_009520 | gi|313879596|gb|AELG01002619.1| | 187447 | 199469 | 5 | 1 |
| XLOC\_009523 | gi|313879597|gb|AELG01002618.1| | 38835 | 67984 | 28 | 3 |
| XLOC\_009530 | gi|313879598|gb|AELG01002617.1| | 20253 | 33182 | 23 | 1 |
| XLOC\_009533 | gi|313879600|gb|AELG01002615.1| | 20565 | 32521 | 5 | 1 |
| XLOC\_009539 XLOC\_009540 XLOC\_009538 | gi|313879602|gb|AELG01002613.1| | 1331 | 11960 | 12 | 1 |
| XLOC\_009559 | gi|313879606|gb|AELG01002609.1| | 712 | 14400 | 10 | 2 |
| XLOC\_009567 | gi|313879608|gb|AELG01002607.1| | 16777 | 20723 | 10 | 2 |
| XLOC\_009579 XLOC\_009578 XLOC\_009581 | gi|313879610|gb|AELG01002605.1| | 1818 | 17704 | 34 | 3 |
| XLOC\_009593 XLOC\_009597 | gi|313879627|gb|AELG01002589.1| | 1814 | 16771 | 51 | 2 |
| XLOC\_009612 XLOC\_009613 | gi|313879629|gb|AELG01002587.1| | 258 | 52210 | 7 | 1 |
| XLOC\_009615 | gi|313879630|gb|AELG01002586.1| | 3810 | 10310 | 10 | 1 |
| XLOC\_009627 XLOC\_009626 | gi|313879632|gb|AELG01002584.1| | 4691 | 12635 | 37 | 1 |
| XLOC\_009638 | gi|313879635|gb|AELG01002582.1| | 1403 | 5883 | 16 | 1 |
| XLOC\_009644 XLOC\_009643 XLOC\_009649 | gi|313879636|gb|AELG01002581.1| | 7275 | 18221 | 34 | 2 |
| XLOC\_009665 | gi|313879637|gb|AELG01002580.1| | 17061 | 19898 | 3 | 1 |
| XLOC\_009666 | gi|313879637|gb|AELG01002580.1| | 20695 | 28482 | 14 | 1 |
| XLOC\_009667 | gi|313879637|gb|AELG01002580.1| | 35146 | 42525 | 19 | 2 |
| XLOC\_009676 XLOC\_009678 | gi|313879639|gb|AELG01002578.1| | 23664 | 33650 | 30 | 3 |
| XLOC\_009677 XLOC\_009674 | gi|313879639|gb|AELG01002578.1| | 6 | 35906 | 55 | 4 |
| XLOC\_009679 | gi|313879640|gb|AELG01002577.1| | 35089 | 48447 | 21 | 1 |
| XLOC\_009688 XLOC\_009684 | gi|313879641|gb|AELG01002576.1| | 13300 | 22665 | 14 | 1 |
| XLOC\_009713 | gi|313879644|gb|AELG01002573.1| | 450 | 15373 | 18 | 7 |
| XLOC\_009714 | gi|313879644|gb|AELG01002573.1| | 15533 | 32203 | 24 | 2 |
| XLOC\_009719 XLOC\_009724 | gi|313879645|gb|AELG01002572.1| | 26898 | 37637 | 16 | 2 |
| XLOC\_009721 XLOC\_009726 | gi|313879645|gb|AELG01002572.1| | 43615 | 49798 | 20 | 2 |
| XLOC\_009730 | gi|313879650|gb|AELG01002567.1| | 6993 | 12568 | 7 | 4 |
| XLOC\_009732 | gi|313879651|gb|AELG01002566.1| | 51382 | 60177 | 11 | 1 |
| XLOC\_009733 XLOC\_009735 | gi|313879651|gb|AELG01002566.1| | 1 | 49287 | 41 | 1 |
| XLOC\_009739 | gi|313879652|gb|AELG01002565.1| | 17620 | 20495 | 11 | 1 |
| XLOC\_009743 | gi|313879652|gb|AELG01002565.1| | 20950 | 23286 | 7 | 1 |
| XLOC\_009746 XLOC\_009744 XLOC\_009740 | gi|313879652|gb|AELG01002565.1| | 24176 | 36351 | 34 | 2 |
| XLOC\_009748 | gi|313879653|gb|AELG01002564.1| | 4434 | 16332 | 18 | 4 |
| XLOC\_009750 XLOC\_009753 | gi|313879653|gb|AELG01002564.1| | 31494 | 56994 | 16 | 1 |
| XLOC\_009752 XLOC\_009749 | gi|313879653|gb|AELG01002564.1| | 16580 | 31374 | 31 | 1 |
| XLOC\_009766 | gi|313879659|gb|AELG01002558.1| | 28580 | 39672 | 9 | 2 |
| XLOC\_009772 | gi|313879662|gb|AELG01002555.1| | 87524 | 97614 | 15 | 1 |
| XLOC\_009777 XLOC\_009783 | gi|313879662|gb|AELG01002555.1| | 120970 | 148272 | 44 | 2 |
| XLOC\_009780 XLOC\_009774 XLOC\_009773 | gi|313879662|gb|AELG01002555.1| | 98283 | 109088 | 22 | 1 |
| XLOC\_009782 | gi|313879662|gb|AELG01002555.1| | 116501 | 119944 | 8 | 1 |
| XLOC\_009804 | gi|313879675|gb|AELG01002543.1| | 11 | 24344 | 6 | 4 |
| XLOC\_009805 | gi|313879676|gb|AELG01002542.1| | 17770 | 26506 | 11 | 1 |
| XLOC\_009809 | gi|313879676|gb|AELG01002542.1| | 52978 | 59261 | 4 | 1 |
| XLOC\_009819 | gi|313879685|gb|AELG01002533.1| | 13566 | 150345 | 37 | 1 |
| XLOC\_009827 XLOC\_009824 | gi|313879686|gb|AELG01002532.1| | 38820 | 57174 | 34 | 2 |
| XLOC\_009842 XLOC\_009845 | gi|313879694|gb|AELG01002525.1| | 52393 | 136375 | 59 | 12 |
| XLOC\_009846 | gi|313879694|gb|AELG01002525.1| | 158694 | 165165 | 20 | 3 |
| XLOC\_009853 | gi|313879695|gb|AELG01002524.1| | 5217 | 10976 | 2 | 2 |
| XLOC\_009904 | gi|313879741|gb|AELG01002479.1| | 99151 | 103815 | 10 | 1 |
| XLOC\_009916 XLOC\_009915 | gi|313879744|gb|AELG01002476.1| | 614 | 6738 | 5 | 2 |
| XLOC\_009944 | gi|313879780|gb|AELG01002442.1| | 32 | 7216 | 9 | 3 |
| XLOC\_009952 XLOC\_009945 XLOC\_009949 | gi|313879781|gb|AELG01002441.1| | 5395 | 25145 | 35 | 4 |
| XLOC\_009955 | gi|313879782|gb|AELG01002440.1| | 10058 | 13465 | 6 | 1 |
| XLOC\_009962 XLOC\_009977 | gi|313879783|gb|AELG01002439.1| | 99207 | 130036 | 35 | 4 |
| XLOC\_009967 | gi|313879783|gb|AELG01002439.1| | 184682 | 188950 | 9 | 1 |
| XLOC\_009981 XLOC\_009965 | gi|313879783|gb|AELG01002439.1| | 150798 | 162779 | 34 | 2 |
| XLOC\_009982 | gi|313879783|gb|AELG01002439.1| | 164338 | 173312 | 10 | 1 |
| XLOC\_009984 XLOC\_009985 XLOC\_009983 XLOC\_009966 | gi|313879783|gb|AELG01002439.1| | 173965 | 183428 | 56 | 2 |
| XLOC\_010003 XLOC\_009995 | gi|313879784|gb|AELG01002438.1| | 51215 | 92245 | 19 | 2 |
| XLOC\_010006 | gi|313879784|gb|AELG01002438.1| | 133400 | 139506 | 19 | 4 |
| XLOC\_010013 | gi|313879784|gb|AELG01002438.1| | 162414 | 166479 | 9 | 1 |
| XLOC\_010021 | gi|313879786|gb|AELG01002436.1| | 19874 | 29883 | 4 | 3 |
| XLOC\_010026 | gi|313879793|gb|AELG01002429.1| | 4158 | 37629 | 7 | 1 |
| XLOC\_010038 | gi|313879795|gb|AELG01002427.1| | 20932 | 31373 | 23 | 3 |
| XLOC\_010043 | gi|313879797|gb|AELG01002425.1| | 24 | 111270 | 14 | 2 |
| XLOC\_010046 | gi|313879798|gb|AELG01002424.1| | 118774 | 121205 | 10 | 1 |
| XLOC\_010049 | gi|313879798|gb|AELG01002424.1| | 223675 | 239526 | 14 | 2 |
| XLOC\_010064 XLOC\_010048 XLOC\_010050 | gi|313879798|gb|AELG01002424.1| | 212383 | 227990 | 25 | 3 |
| XLOC\_010082 | gi|313879799|gb|AELG01002423.1| | 27730 | 35080 | 11 | 4 |
| XLOC\_010091 XLOC\_010088 | gi|313879800|gb|AELG01002422.1| | 36647 | 94809 | 29 | 1 |
| XLOC\_010101 | gi|313879808|gb|AELG01002416.1| | 1 | 26917 | 4 | 1 |
| XLOC\_010104 | gi|313879812|gb|AELG01002415.1| | 42706 | 50701 | 2 | 2 |
| XLOC\_010109 | gi|313879813|gb|AELG01002414.1| | 3 | 27769 | 5 | 2 |
| XLOC\_010128 XLOC\_010123 | gi|313879818|gb|AELG01002409.1| | 5270 | 20143 | 16 | 1 |
| XLOC\_010133 XLOC\_010126 | gi|313879818|gb|AELG01002409.1| | 77845 | 86751 | 27 | 1 |
| XLOC\_010134 | gi|313879818|gb|AELG01002409.1| | 170939 | 184128 | 18 | 2 |
| XLOC\_010144 | gi|313879821|gb|AELG01002406.1| | 191 | 8429 | 17 | 5 |
| XLOC\_010146 | gi|313879821|gb|AELG01002406.1| | 28621 | 69635 | 14 | 3 |
| XLOC\_010149 | gi|313879821|gb|AELG01002406.1| | 127176 | 142347 | 7 | 1 |
| XLOC\_010156 | gi|313879821|gb|AELG01002406.1| | 18317 | 24814 | 10 | 1 |
| XLOC\_010177 | gi|313879830|gb|AELG01002397.1| | 80414 | 89337 | 5 | 1 |
| XLOC\_010201 XLOC\_010198 | gi|313879840|gb|AELG01002388.1| | 112767 | 123495 | 33 | 4 |
| XLOC\_010202 XLOC\_010199 | gi|313879840|gb|AELG01002388.1| | 123817 | 132783 | 29 | 8 |
| XLOC\_010226 XLOC\_010235 | gi|313879849|gb|AELG01002379.1| | 11268 | 14728 | 10 | 2 |
| XLOC\_010228 | gi|313879849|gb|AELG01002379.1| | 19403 | 27470 | 21 | 3 |
| XLOC\_010240 | gi|313879849|gb|AELG01002379.1| | 63315 | 73917 | 13 | 2 |
| XLOC\_010244 | gi|313879849|gb|AELG01002379.1| | 87844 | 100157 | 6 | 3 |
| XLOC\_010252 XLOC\_010254 | gi|313879850|gb|AELG01002378.1| | 8358 | 23800 | 29 | 1 |
| XLOC\_010253 XLOC\_010255 | gi|313879850|gb|AELG01002378.1| | 24082 | 34586 | 17 | 1 |
| XLOC\_010268 | gi|313879853|gb|AELG01002375.1| | 119726 | 124578 | 8 | 3 |
| XLOC\_010269 | gi|313879853|gb|AELG01002375.1| | 154068 | 347532 | 7 | 6 |
| XLOC\_010276 | gi|313879853|gb|AELG01002375.1| | 41041 | 115464 | 32 | 1 |
| XLOC\_010277 | gi|313879853|gb|AELG01002375.1| | 361630 | 373562 | 13 | 1 |
| XLOC\_010293 | gi|313879857|gb|AELG01002371.1| | 7055 | 16907 | 10 | 1 |
| XLOC\_010294 | gi|313879857|gb|AELG01002371.1| | 18055 | 25394 | 15 | 1 |
| XLOC\_010311 | gi|313879859|gb|AELG01002369.1| | 28798 | 34459 | 15 | 1 |
| XLOC\_010314 | gi|313879861|gb|AELG01002368.1| | 9081 | 18240 | 25 | 1 |
| XLOC\_010352 | gi|313879868|gb|AELG01002361.1| | 20993 | 28292 | 10 | 1 |
| XLOC\_010358 | gi|313879869|gb|AELG01002360.1| | 1707 | 9022 | 8 | 3 |
| XLOC\_010364 XLOC\_010361 | gi|313879869|gb|AELG01002360.1| | 23053 | 33168 | 27 | 1 |
| XLOC\_010366 | gi|313879870|gb|AELG01002359.1| | 254 | 3635 | 7 | 1 |
| XLOC\_010372 | gi|313879870|gb|AELG01002359.1| | 18440 | 28270 | 10 | 1 |
| XLOC\_010375 XLOC\_010377 | gi|313879871|gb|AELG01002358.1| | 8994 | 26213 | 27 | 1 |
| XLOC\_010376 | gi|313879871|gb|AELG01002358.1| | 27190 | 34686 | 22 | 1 |
| XLOC\_010403 | gi|313879875|gb|AELG01002354.1| | 66695 | 71509 | 8 | 4 |
| XLOC\_010409 | gi|313879877|gb|AELG01002352.1| | 5348 | 16623 | 6 | 1 |
| XLOC\_010416 XLOC\_010412 | gi|313879878|gb|AELG01002351.1| | 28971 | 50750 | 46 | 4 |
| XLOC\_010450 XLOC\_010449 | gi|313879894|gb|AELG01002335.1| | 4993 | 30353 | 27 | 1 |
| XLOC\_010470 | gi|313879933|gb|AELG01002298.1| | 21132 | 29281 | 9 | 2 |
| XLOC\_010476 | gi|313879933|gb|AELG01002298.1| | 37233 | 71621 | 10 | 1 |
| XLOC\_010486 XLOC\_010489 | gi|313879935|gb|AELG01002296.1| | 141028 | 148799 | 17 | 1 |
| XLOC\_010495 | gi|313879937|gb|AELG01002294.1| | 435 | 11654 | 5 | 3 |
| XLOC\_010537 XLOC\_010536 XLOC\_010526 XLOC\_010530 | gi|313879946|gb|AELG01002285.1| | 9853 | 31411 | 33 | 5 |
| XLOC\_010549 XLOC\_010546 | gi|313879951|gb|AELG01002281.1| | 3245 | 9457 | 29 | 1 |
| XLOC\_010559 | gi|313879954|gb|AELG01002278.1| | 19259 | 22711 | 3 | 1 |
| XLOC\_010560 XLOC\_010558 | gi|313879954|gb|AELG01002278.1| | 8304 | 17584 | 13 | 5 |
| XLOC\_010576 | gi|313879958|gb|AELG01002274.1| | 43160 | 47688 | 6 | 1 |
| XLOC\_010591 | gi|313879959|gb|AELG01002273.1| | 129 | 3652 | 4 | 1 |
| XLOC\_010595 XLOC\_010594 XLOC\_010588 | gi|313879959|gb|AELG01002273.1| | 43758 | 88285 | 61 | 4 |
| XLOC\_010597 XLOC\_010589 | gi|313879959|gb|AELG01002273.1| | 95289 | 97647 | 9 | 2 |
| XLOC\_010604 XLOC\_010607 | gi|313879960|gb|AELG01002272.1| | 553 | 12951 | 37 | 3 |
| XLOC\_010608 | gi|313879960|gb|AELG01002272.1| | 13344 | 19139 | 15 | 3 |
| XLOC\_010620 XLOC\_010616 XLOC\_010619 | gi|313879961|gb|AELG01002271.1| | 52 | 10860 | 53 | 4 |
| XLOC\_010630 XLOC\_010637 | gi|313879962|gb|AELG01002270.1| | 4205 | 7367 | 6 | 1 |
| XLOC\_010632 XLOC\_010633 XLOC\_010625 | gi|313879962|gb|AELG01002270.1| | 12504 | 40109 | 36 | 1 |
| XLOC\_010635 | gi|313879962|gb|AELG01002270.1| | 60427 | 76984 | 35 | 5 |
| XLOC\_010646 | gi|313879963|gb|AELG01002269.1| | 17097 | 22753 | 19 | 1 |
| XLOC\_010655 | gi|313879965|gb|AELG01002267.1| | 12627 | 29059 | 14 | 1 |
| XLOC\_010657 | gi|313879965|gb|AELG01002267.1| | 50122 | 56985 | 10 | 1 |
| XLOC\_010663 XLOC\_010668 | gi|313879966|gb|AELG01002266.1| | 34068 | 46017 | 21 | 1 |
| XLOC\_010697 XLOC\_010702 | gi|313879969|gb|AELG01002264.1| | 29532 | 48909 | 50 | 3 |
| XLOC\_010698 XLOC\_010703 | gi|313879969|gb|AELG01002264.1| | 49107 | 65650 | 31 | 1 |
| XLOC\_010700 | gi|313879969|gb|AELG01002264.1| | 3167 | 17266 | 20 | 3 |
| XLOC\_010708 | gi|313879970|gb|AELG01002263.1| | 6011 | 14287 | 8 | 3 |
| XLOC\_010713 XLOC\_010710 | gi|313879971|gb|AELG01002262.1| | 14089 | 17821 | 16 | 1 |
| XLOC\_010729 | gi|313879976|gb|AELG01002257.1| | 21567 | 35200 | 30 | 2 |
| XLOC\_010733 | gi|313879977|gb|AELG01002256.1| | 12981 | 68265 | 5 | 4 |
| XLOC\_010734 XLOC\_010746 | gi|313879977|gb|AELG01002256.1| | 93901 | 106845 | 29 | 2 |
| XLOC\_010758 | gi|313879978|gb|AELG01002255.1| | 32099 | 104474 | 23 | 2 |
| XLOC\_010761 | gi|313879978|gb|AELG01002255.1| | 147798 | 155034 | 4 | 1 |
| XLOC\_010770 | gi|313879978|gb|AELG01002255.1| | 140708 | 146080 | 11 | 1 |
| XLOC\_010780 | gi|313879981|gb|AELG01002252.1| | 7186 | 79372 | 19 | 2 |
| XLOC\_010784 XLOC\_010781 | gi|313879981|gb|AELG01002252.1| | 83160 | 87823 | 24 | 1 |
| XLOC\_010789 | gi|313879982|gb|AELG01002251.1| | 13549 | 46220 | 15 | 1 |
| XLOC\_010796 | gi|313879985|gb|AELG01002248.1| | 8933 | 14044 | 15 | 1 |
| XLOC\_010807 XLOC\_010806 | gi|313880001|gb|AELG01002233.1| | 425 | 4116 | 15 | 1 |
| XLOC\_010814 XLOC\_010816 | gi|313880007|gb|AELG01002227.1| | 26582 | 30348 | 10 | 1 |
| XLOC\_010815 | gi|313880007|gb|AELG01002227.1| | 30601 | 35838 | 16 | 2 |
| XLOC\_010819 | gi|313880008|gb|AELG01002226.1| | 9262 | 12373 | 12 | 1 |
| XLOC\_010830 | gi|313880009|gb|AELG01002225.1| | 1212 | 6851 | 8 | 1 |
| XLOC\_010831 XLOC\_010828 | gi|313880009|gb|AELG01002225.1| | 7513 | 17003 | 21 | 1 |
| XLOC\_010834 XLOC\_010835 | gi|313880010|gb|AELG01002224.1| | 18115 | 41375 | 24 | 6 |
| XLOC\_010838 | gi|313880012|gb|AELG01002222.1| | 51636 | 57084 | 9 | 1 |
| XLOC\_010842 | gi|313880013|gb|AELG01002221.1| | 4306 | 32366 | 8 | 2 |
| XLOC\_010845 XLOC\_010849 | gi|313880013|gb|AELG01002221.1| | 44011 | 75169 | 71 | 33 |
| XLOC\_010846 | gi|313880013|gb|AELG01002221.1| | 143686 | 150345 | 7 | 1 |
| XLOC\_010850 | gi|313880013|gb|AELG01002221.1| | 75258 | 105738 | 40 | 2 |
| XLOC\_010854 XLOC\_010847 | gi|313880013|gb|AELG01002221.1| | 150536 | 164694 | 12 | 1 |
| XLOC\_010863 | gi|313880017|gb|AELG01002218.1| | 1 | 40370 | 24 | 5 |
| XLOC\_010867 XLOC\_010871 | gi|313880018|gb|AELG01002217.1| | 812 | 12001 | 33 | 3 |
| XLOC\_010868 XLOC\_010872 XLOC\_010873 | gi|313880018|gb|AELG01002217.1| | 12268 | 40659 | 58 | 13 |
| XLOC\_010876 XLOC\_010887 | gi|313880022|gb|AELG01002213.1| | 16172 | 34623 | 29 | 1 |
| XLOC\_010877 | gi|313880022|gb|AELG01002213.1| | 35490 | 42277 | 14 | 2 |
| XLOC\_010909 | gi|313880033|gb|AELG01002202.1| | 414 | 11213 | 22 | 4 |
| XLOC\_010916 | gi|313880033|gb|AELG01002202.1| | 25142 | 41505 | 19 | 1 |
| XLOC\_010935 | gi|313880040|gb|AELG01002195.1| | 30459 | 44339 | 12 | 4 |
| XLOC\_010936 | gi|313880040|gb|AELG01002195.1| | 49864 | 56276 | 13 | 1 |
| XLOC\_010965 XLOC\_010972 | gi|313880046|gb|AELG01002189.1| | 1215 | 35877 | 18 | 2 |
| XLOC\_010968 | gi|313880046|gb|AELG01002189.1| | 52015 | 73342 | 20 | 1 |
| XLOC\_010985 | gi|313880051|gb|AELG01002185.1| | 9601 | 27672 | 6 | 1 |
| XLOC\_010998 | gi|313880059|gb|AELG01002177.1| | 467 | 15200 | 25 | 1 |
| XLOC\_011003 XLOC\_011010 XLOC\_011007 | gi|313880059|gb|AELG01002177.1| | 48916 | 59362 | 16 | 1 |
| XLOC\_011018 | gi|313880061|gb|AELG01002175.1| | 14221 | 32473 | 11 | 3 |
| XLOC\_011022 | gi|313880063|gb|AELG01002173.1| | 7473 | 25411 | 34 | 3 |
| XLOC\_011051 XLOC\_011060 | gi|313880067|gb|AELG01002169.1| | 97355 | 108311 | 31 | 1 |
| XLOC\_011052 | gi|313880067|gb|AELG01002169.1| | 30730 | 36296 | 10 | 1 |
| XLOC\_011053 | gi|313880067|gb|AELG01002169.1| | 38516 | 45948 | 3 | 1 |
| XLOC\_011055 | gi|313880067|gb|AELG01002169.1| | 54411 | 63314 | 26 | 4 |
| XLOC\_011070 | gi|313880072|gb|AELG01002164.1| | 49 | 4063 | 4 | 1 |
| XLOC\_011071 | gi|313880073|gb|AELG01002163.1| | 31329 | 55341 | 10 | 4 |
| XLOC\_011110 | gi|313880101|gb|AELG01002136.1| | 15347 | 36039 | 6 | 1 |
| XLOC\_011160 | gi|313880156|gb|AELG01002084.1| | 1 | 2306 | 3 | 2 |
| XLOC\_011182 | gi|313880186|gb|AELG01002055.1| | 505 | 14627 | 6 | 1 |
| XLOC\_011188 | gi|313880197|gb|AELG01002044.1| | 3144 | 54057 | 26 | 1 |
| XLOC\_011225 | gi|313880205|gb|AELG01002036.1| | 14104 | 26007 | 18 | 2 |
| XLOC\_011228 | gi|313880207|gb|AELG01002035.1| | 3298 | 8681 | 14 | 1 |
| XLOC\_011234 | gi|313880207|gb|AELG01002035.1| | 12328 | 20631 | 14 | 5 |
| XLOC\_011251 | gi|313880210|gb|AELG01002032.1| | 147 | 12305 | 16 | 1 |
| XLOC\_011261 | gi|313880212|gb|AELG01002030.1| | 3881 | 29286 | 17 | 2 |
| XLOC\_011267 XLOC\_011269 | gi|313880217|gb|AELG01002025.1| | 3670 | 40932 | 50 | 1 |
| XLOC\_011297 XLOC\_011291 | gi|313880226|gb|AELG01002017.1| | 15270 | 33450 | 26 | 5 |
| XLOC\_011302 | gi|313880227|gb|AELG01002016.1| | 98 | 5137 | 10 | 1 |
| XLOC\_011304 | gi|313880227|gb|AELG01002016.1| | 5372 | 9916 | 12 | 2 |
| XLOC\_011326 | gi|313880247|gb|AELG01001996.1| | 68665 | 126401 | 11 | 1 |
| XLOC\_011335 | gi|313880262|gb|AELG01001982.1| | 59026 | 89337 | 16 | 1 |
| XLOC\_011371 | gi|313880275|gb|AELG01001969.1| | 5 | 2860 | 7 | 1 |
| XLOC\_011372 XLOC\_011373 | gi|313880275|gb|AELG01001969.1| | 3086 | 16125 | 34 | 7 |
| XLOC\_011375 | gi|313880276|gb|AELG01001968.1| | 10566 | 15622 | 10 | 1 |
| XLOC\_011378 | gi|313880276|gb|AELG01001968.1| | 27924 | 39882 | 23 | 1 |
| XLOC\_011394 XLOC\_011396 | gi|313880277|gb|AELG01001967.1| | 94307 | 103382 | 18 | 1 |
| XLOC\_011398 | gi|313880277|gb|AELG01001967.1| | 107965 | 117856 | 24 | 1 |
| XLOC\_011412 | gi|313880279|gb|AELG01001965.1| | 28800 | 40954 | 27 | 3 |
| XLOC\_011436 | gi|313880289|gb|AELG01001955.1| | 7505 | 25197 | 10 | 3 |
| XLOC\_011445 | gi|313880296|gb|AELG01001949.1| | 134 | 30634 | 28 | 1 |
| XLOC\_011446 | gi|313880296|gb|AELG01001949.1| | 40079 | 45657 | 7 | 1 |
| XLOC\_011455 XLOC\_011454 XLOC\_011460 | gi|313880298|gb|AELG01001947.1| | 27825 | 37432 | 39 | 1 |
| XLOC\_011459 | gi|313880298|gb|AELG01001947.1| | 17730 | 26236 | 8 | 1 |
| XLOC\_011467 | gi|313880299|gb|AELG01001946.1| | 1 | 6255 | 9 | 1 |
| XLOC\_011469 XLOC\_011473 | gi|313880300|gb|AELG01001945.1| | 7406 | 13064 | 15 | 1 |
| XLOC\_011472 | gi|313880300|gb|AELG01001945.1| | 2363 | 6414 | 10 | 1 |
| XLOC\_011481 | gi|313880301|gb|AELG01001944.1| | 33524 | 39253 | 20 | 2 |
| XLOC\_011506 | gi|313880312|gb|AELG01001933.1| | 3135 | 8737 | 9 | 1 |
| XLOC\_011512 XLOC\_011515 | gi|313880320|gb|AELG01001926.1| | 34 | 4562 | 16 | 2 |
| XLOC\_011513 | gi|313880320|gb|AELG01001926.1| | 4807 | 7699 | 7 | 1 |
| XLOC\_011524 | gi|313880322|gb|AELG01001924.1| | 1 | 57381 | 25 | 11 |
| XLOC\_011553 | gi|313880330|gb|AELG01001917.1| | 3956 | 9259 | 10 | 3 |
| XLOC\_011562 | gi|313880332|gb|AELG01001915.1| | 23133 | 45493 | 25 | 1 |
| XLOC\_011598 | gi|313880334|gb|AELG01001913.1| | 32671 | 37960 | 2 | 2 |
| XLOC\_011630 | gi|313880345|gb|AELG01001903.1| | 94 | 12447 | 28 | 1 |
| XLOC\_011632 | gi|313880345|gb|AELG01001903.1| | 31306 | 35389 | 8 | 1 |
| XLOC\_011637 | gi|313880345|gb|AELG01001903.1| | 26333 | 30906 | 9 | 1 |
| XLOC\_011644 | gi|313880346|gb|AELG01001902.1| | 205 | 8377 | 14 | 3 |
| XLOC\_011659 | gi|313880347|gb|AELG01001901.1| | 13488 | 33523 | 12 | 2 |
| XLOC\_011660 XLOC\_011662 | gi|313880347|gb|AELG01001901.1| | 34122 | 40301 | 15 | 2 |
| XLOC\_011666 | gi|313880348|gb|AELG01001900.1| | 6952 | 11056 | 7 | 1 |
| XLOC\_011677 | gi|313880349|gb|AELG01001899.1| | 22787 | 28899 | 3 | 1 |
| XLOC\_011678 XLOC\_011683 | gi|313880349|gb|AELG01001899.1| | 47073 | 56006 | 14 | 1 |
| XLOC\_011693 | gi|313880353|gb|AELG01001895.1| | 14324 | 28986 | 10 | 1 |
| XLOC\_011698 XLOC\_011700 | gi|313880354|gb|AELG01001894.1| | 53406 | 74890 | 11 | 1 |
| XLOC\_011701 | gi|313880355|gb|AELG01001893.1| | 7619 | 11502 | 14 | 1 |
| XLOC\_011703 | gi|313880355|gb|AELG01001893.1| | 4 | 7460 | 23 | 1 |
| XLOC\_011704 | gi|313880355|gb|AELG01001893.1| | 11702 | 21730 | 24 | 6 |
| XLOC\_011721 | gi|313880357|gb|AELG01001891.1| | 51315 | 83410 | 18 | 6 |
| XLOC\_011723 | gi|313880357|gb|AELG01001891.1| | 89454 | 120505 | 4 | 3 |
| XLOC\_011744 XLOC\_011739 | gi|313880373|gb|AELG01001876.1| | 524 | 7100 | 15 | 1 |
| XLOC\_011746 | gi|313880373|gb|AELG01001876.1| | 10553 | 15699 | 16 | 1 |
| XLOC\_011747 | gi|313880373|gb|AELG01001876.1| | 17030 | 26161 | 15 | 3 |
| XLOC\_011761 | gi|313880374|gb|AELG01001875.1| | 73758 | 77628 | 4 | 2 |
| XLOC\_011764 | gi|313880374|gb|AELG01001875.1| | 27930 | 43640 | 11 | 1 |
| XLOC\_011770 XLOC\_011762 | gi|313880374|gb|AELG01001875.1| | 78863 | 96721 | 31 | 7 |
| XLOC\_011771 | gi|313880374|gb|AELG01001875.1| | 99283 | 109225 | 31 | 11 |
| XLOC\_011779 | gi|313880375|gb|AELG01001874.1| | 19 | 3537 | 7 | 1 |
| XLOC\_011794 XLOC\_011790 | gi|313880377|gb|AELG01001872.1| | 54162 | 77916 | 28 | 6 |
| XLOC\_011804 | gi|313880386|gb|AELG01001864.1| | 24360 | 39029 | 21 | 10 |
| XLOC\_011815 | gi|313880387|gb|AELG01001863.1| | 33585 | 40257 | 7 | 1 |
| XLOC\_011822 | gi|313880387|gb|AELG01001863.1| | 77848 | 92185 | 27 | 2 |
| XLOC\_011830 XLOC\_011825 | gi|313880387|gb|AELG01001863.1| | 97895 | 118523 | 53 | 17 |
| XLOC\_011834 XLOC\_011837 | gi|313880388|gb|AELG01001862.1| | 553 | 9189 | 30 | 2 |
| XLOC\_011836 | gi|313880388|gb|AELG01001862.1| | 17705 | 23728 | 11 | 3 |
| XLOC\_011848 | gi|313880410|gb|AELG01001840.1| | 2 | 4091 | 8 | 1 |
| XLOC\_011849 | gi|313880414|gb|AELG01001836.1| | 24259 | 60714 | 7 | 1 |
| XLOC\_011862 | gi|313880423|gb|AELG01001828.1| | 24603 | 38798 | 4 | 1 |
| XLOC\_011875 | gi|313880424|gb|AELG01001827.1| | 27086 | 35972 | 13 | 1 |
| XLOC\_011878 | gi|313880424|gb|AELG01001827.1| | 50130 | 96801 | 18 | 1 |
| XLOC\_011880 XLOC\_011870 | gi|313880424|gb|AELG01001827.1| | 100024 | 108033 | 19 | 1 |
| XLOC\_011888 | gi|313880429|gb|AELG01001822.1| | 58580 | 79345 | 3 | 2 |
| XLOC\_011894 | gi|313880433|gb|AELG01001818.1| | 28739 | 32434 | 11 | 1 |
| XLOC\_011895 | gi|313880433|gb|AELG01001818.1| | 1 | 23211 | 55 | 9 |
| XLOC\_011915 XLOC\_011921 | gi|313880436|gb|AELG01001815.1| | 45376 | 80441 | 33 | 5 |
| XLOC\_011916 XLOC\_011924 XLOC\_011931 | gi|313880436|gb|AELG01001815.1| | 156696 | 210651 | 29 | 3 |
| XLOC\_011925 | gi|313880436|gb|AELG01001815.1| | 211058 | 214091 | 7 | 1 |
| XLOC\_011935 XLOC\_011933 | gi|313880437|gb|AELG01001814.1| | 3400 | 33123 | 50 | 10 |
| XLOC\_011951 XLOC\_011955 | gi|313880440|gb|AELG01001811.1| | 42801 | 60703 | 15 | 1 |
| XLOC\_011952 | gi|313880440|gb|AELG01001811.1| | 61524 | 68415 | 6 | 1 |
| XLOC\_011953 XLOC\_011948 | gi|313880440|gb|AELG01001811.1| | 15531 | 25138 | 24 | 1 |
| XLOC\_011960 | gi|313880441|gb|AELG01001810.1| | 45302 | 48395 | 8 | 2 |
| XLOC\_011980 XLOC\_011982 XLOC\_011979 | gi|313880447|gb|AELG01001805.1| | 35934 | 48772 | 41 | 7 |
| XLOC\_011990 | gi|313880449|gb|AELG01001803.1| | 26544 | 94048 | 22 | 5 |
| XLOC\_011991 | gi|313880449|gb|AELG01001803.1| | 95074 | 100411 | 10 | 1 |
| XLOC\_012012 | gi|313880457|gb|AELG01001796.1| | 19355 | 35505 | 10 | 1 |
| XLOC\_012030 XLOC\_012028 | gi|313880458|gb|AELG01001795.1| | 13 | 12646 | 24 | 1 |
| XLOC\_012033 | gi|313880459|gb|AELG01001794.1| | 3574 | 12075 | 24 | 1 |
| XLOC\_012039 XLOC\_012043 | gi|313880459|gb|AELG01001794.1| | 42518 | 52645 | 13 | 1 |
| XLOC\_012042 XLOC\_012035 | gi|313880459|gb|AELG01001794.1| | 29963 | 34765 | 18 | 1 |
| XLOC\_012050 | gi|313880460|gb|AELG01001793.1| | 26863 | 46271 | 28 | 2 |
| XLOC\_012060 | gi|313880460|gb|AELG01001793.1| | 106438 | 110950 | 16 | 1 |
| XLOC\_012074 XLOC\_012094 | gi|313880466|gb|AELG01001787.1| | 39311 | 46072 | 19 | 1 |
| XLOC\_012075 | gi|313880466|gb|AELG01001787.1| | 53776 | 60958 | 12 | 1 |
| XLOC\_012076 XLOC\_012077 XLOC\_012096 | gi|313880466|gb|AELG01001787.1| | 61476 | 77062 | 32 | 1 |
| XLOC\_012079 | gi|313880466|gb|AELG01001787.1| | 94553 | 103670 | 11 | 5 |
| XLOC\_012088 | gi|313880466|gb|AELG01001787.1| | 194702 | 203033 | 30 | 1 |
| XLOC\_012090 | gi|313880466|gb|AELG01001787.1| | 235661 | 251443 | 40 | 2 |
| XLOC\_012092 | gi|313880466|gb|AELG01001787.1| | 267528 | 270497 | 2 | 2 |
| XLOC\_012093 | gi|313880466|gb|AELG01001787.1| | 45 | 36637 | 9 | 7 |
| XLOC\_012095 | gi|313880466|gb|AELG01001787.1| | 46410 | 52883 | 9 | 1 |
| XLOC\_012099 | gi|313880466|gb|AELG01001787.1| | 87697 | 92295 | 10 | 1 |
| XLOC\_012104 XLOC\_012085 | gi|313880466|gb|AELG01001787.1| | 136500 | 165341 | 40 | 1 |
| XLOC\_012107 | gi|313880466|gb|AELG01001787.1| | 212946 | 216600 | 11 | 1 |
| XLOC\_012108 | gi|313880466|gb|AELG01001787.1| | 216953 | 232090 | 15 | 1 |
| XLOC\_012121 | gi|313880467|gb|AELG01001786.1| | 27362 | 34027 | 6 | 1 |
| XLOC\_012122 XLOC\_012119 | gi|313880467|gb|AELG01001786.1| | 4254 | 17312 | 45 | 4 |
| XLOC\_012123 | gi|313880467|gb|AELG01001786.1| | 17690 | 22160 | 15 | 1 |
| XLOC\_012134 XLOC\_012137 | gi|313880470|gb|AELG01001783.1| | 7471 | 27953 | 27 | 1 |
| XLOC\_012136 | gi|313880470|gb|AELG01001783.1| | 8318 | 12133 | 14 | 1 |
| XLOC\_012138 | gi|313880471|gb|AELG01001782.1| | 26431 | 30010 | 12 | 1 |
| XLOC\_012140 XLOC\_012139 XLOC\_012148 | gi|313880471|gb|AELG01001782.1| | 33294 | 39075 | 16 | 1 |
| XLOC\_012149 XLOC\_012141 | gi|313880471|gb|AELG01001782.1| | 39232 | 43876 | 19 | 3 |
| XLOC\_012151 XLOC\_012144 | gi|313880471|gb|AELG01001782.1| | 52651 | 58572 | 14 | 1 |
| XLOC\_012157 | gi|313880472|gb|AELG01001781.1| | 3087 | 16609 | 6 | 3 |
| XLOC\_012165 | gi|313880476|gb|AELG01001778.1| | 15173 | 19022 | 7 | 2 |
| XLOC\_012174 XLOC\_012171 XLOC\_012173 | gi|313880477|gb|AELG01001777.1| | 40676 | 98149 | 55 | 8 |
| XLOC\_012177 | gi|313880478|gb|AELG01001776.1| | 14132 | 19845 | 10 | 6 |
| XLOC\_012179 XLOC\_012180 XLOC\_012185 | gi|313880478|gb|AELG01001776.1| | 30450 | 52526 | 59 | 2 |
| XLOC\_012193 | gi|313880480|gb|AELG01001774.1| | 92918 | 96314 | 6 | 1 |
| XLOC\_012195 XLOC\_012190 | gi|313880480|gb|AELG01001774.1| | 99858 | 108539 | 17 | 1 |
| XLOC\_012214 | gi|313880492|gb|AELG01001763.1| | 95897 | 123827 | 9 | 4 |
| XLOC\_012226 | gi|313880497|gb|AELG01001758.1| | 6763 | 17325 | 5 | 2 |
| XLOC\_012247 | gi|313880505|gb|AELG01001750.1| | 38456 | 47974 | 5 | 1 |
| XLOC\_012248 XLOC\_012254 | gi|313880506|gb|AELG01001749.1| | 426 | 5878 | 18 | 1 |
| XLOC\_012251 | gi|313880506|gb|AELG01001749.1| | 39075 | 43000 | 6 | 1 |
| XLOC\_012255 | gi|313880506|gb|AELG01001749.1| | 19147 | 25680 | 19 | 7 |
| XLOC\_012256 | gi|313880506|gb|AELG01001749.1| | 26566 | 32549 | 19 | 2 |
| XLOC\_012265 XLOC\_012264 | gi|313880510|gb|AELG01001746.1| | 382 | 78050 | 50 | 4 |
| XLOC\_012272 XLOC\_012271 | gi|313880514|gb|AELG01001742.1| | 24188 | 37513 | 17 | 2 |
| XLOC\_012282 | gi|313880515|gb|AELG01001741.1| | 34610 | 72025 | 20 | 3 |
| XLOC\_012284 | gi|313880515|gb|AELG01001741.1| | 25749 | 34366 | 13 | 1 |
| XLOC\_012306 XLOC\_012313 | gi|313880525|gb|AELG01001731.1| | 2931 | 27859 | 41 | 6 |
| XLOC\_012307 | gi|313880525|gb|AELG01001731.1| | 27969 | 31887 | 8 | 2 |
| XLOC\_012318 | gi|313880525|gb|AELG01001731.1| | 73579 | 83087 | 14 | 1 |
| XLOC\_012319 XLOC\_012312 | gi|313880525|gb|AELG01001731.1| | 83188 | 92785 | 29 | 2 |
| XLOC\_012328 XLOC\_012331 | gi|313880526|gb|AELG01001730.1| | 18188 | 32697 | 9 | 5 |
| XLOC\_012329 | gi|313880526|gb|AELG01001730.1| | 988 | 7981 | 5 | 4 |
| XLOC\_012338 | gi|313880527|gb|AELG01001729.1| | 13770 | 40829 | 29 | 3 |
| XLOC\_012344 | gi|313880529|gb|AELG01001727.1| | 116604 | 130233 | 20 | 2 |
| XLOC\_012355 | gi|313880533|gb|AELG01001723.1| | 19929 | 32226 | 4 | 2 |
| XLOC\_012379 XLOC\_012377 | gi|313880540|gb|AELG01001716.1| | 661 | 15561 | 33 | 5 |
| XLOC\_012394 | gi|313880548|gb|AELG01001708.1| | 454 | 21397 | 22 | 12 |
| XLOC\_012395 XLOC\_012397 XLOC\_012396 | gi|313880548|gb|AELG01001708.1| | 25974 | 63180 | 36 | 7 |
| XLOC\_012403 XLOC\_012404 XLOC\_012407 | gi|313880551|gb|AELG01001705.1| | 18345 | 32198 | 38 | 1 |
| XLOC\_012412 | gi|313880556|gb|AELG01001700.1| | 1 | 9831 | 15 | 2 |
| XLOC\_012413 | gi|313880557|gb|AELG01001699.1| | 28352 | 33758 | 7 | 3 |
| XLOC\_012418 | gi|313880566|gb|AELG01001690.1| | 2970 | 15711 | 13 | 2 |
| XLOC\_012419 | gi|313880566|gb|AELG01001690.1| | 16415 | 41134 | 21 | 3 |
| XLOC\_012420 | gi|313880566|gb|AELG01001690.1| | 41524 | 53672 | 16 | 1 |
| XLOC\_012421 XLOC\_012429 | gi|313880566|gb|AELG01001690.1| | 69583 | 76888 | 11 | 1 |
| XLOC\_012427 | gi|313880566|gb|AELG01001690.1| | 58999 | 66942 | 10 | 2 |
| XLOC\_012457 | gi|313880574|gb|AELG01001682.1| | 9265 | 16086 | 3 | 3 |
| XLOC\_012468 | gi|313880582|gb|AELG01001675.1| | 19341 | 28271 | 6 | 2 |
| XLOC\_012470 | gi|313880585|gb|AELG01001672.1| | 196307 | 226785 | 9 | 1 |
| XLOC\_012482 XLOC\_012485 | gi|313880592|gb|AELG01001665.1| | 50123 | 61059 | 22 | 1 |
| XLOC\_012484 | gi|313880592|gb|AELG01001665.1| | 71465 | 76973 | 5 | 1 |
| XLOC\_012500 | gi|313880609|gb|AELG01001648.1| | 2800 | 6634 | 4 | 1 |
| XLOC\_012508 | gi|313880615|gb|AELG01001642.1| | 44173 | 48864 | 9 | 2 |
| XLOC\_012513 XLOC\_012511 XLOC\_012510 | gi|313880615|gb|AELG01001642.1| | 54350 | 61929 | 38 | 1 |
| XLOC\_012522 XLOC\_012520 XLOC\_012519 | gi|313880620|gb|AELG01001637.1| | 2721 | 25069 | 40 | 3 |
| XLOC\_012610 XLOC\_012608 | gi|313880662|gb|AELG01001595.1| | 13153 | 25297 | 31 | 1 |
| XLOC\_012615 XLOC\_012617 | gi|313880664|gb|AELG01001593.1| | 1858 | 8018 | 26 | 1 |
| XLOC\_012621 | gi|313880666|gb|AELG01001591.1| | 45739 | 53950 | 13 | 1 |
| XLOC\_012624 | gi|313880666|gb|AELG01001591.1| | 31278 | 37427 | 11 | 3 |
| XLOC\_012672 | gi|313880704|gb|AELG01001553.1| | 62679 | 85522 | 6 | 1 |
| XLOC\_012716 | gi|313880719|gb|AELG01001538.1| | 1113 | 23415 | 21 | 1 |
| XLOC\_012738 XLOC\_012730 | gi|313880720|gb|AELG01001537.1| | 12127 | 25657 | 18 | 5 |
| XLOC\_012741 | gi|313880720|gb|AELG01001537.1| | 48424 | 75990 | 14 | 5 |
| XLOC\_012761 | gi|313880723|gb|AELG01001534.1| | 16622 | 62004 | 18 | 5 |
| XLOC\_012780 | gi|313880729|gb|AELG01001528.1| | 50180 | 91429 | 9 | 1 |
| XLOC\_012789 | gi|313880731|gb|AELG01001526.1| | 101267 | 121990 | 9 | 3 |
| XLOC\_012801 XLOC\_012799 | gi|313880733|gb|AELG01001524.1| | 5431 | 47805 | 13 | 1 |
| XLOC\_012828 | gi|313880779|gb|AELG01001479.1| | 6141 | 14521 | 10 | 3 |
| XLOC\_012831 | gi|313880779|gb|AELG01001479.1| | 30687 | 95918 | 31 | 8 |
| XLOC\_012832 | gi|313880779|gb|AELG01001479.1| | 97605 | 106324 | 20 | 1 |
| XLOC\_012837 | gi|313880780|gb|AELG01001478.1| | 40512 | 48106 | 12 | 1 |
| XLOC\_012851 | gi|313880782|gb|AELG01001476.1| | 19353 | 30698 | 22 | 4 |
| XLOC\_012867 XLOC\_012859 | gi|313880783|gb|AELG01001475.1| | 14696 | 23520 | 34 | 1 |
| XLOC\_012871 | gi|313880784|gb|AELG01001474.1| | 5293 | 21775 | 7 | 1 |
| XLOC\_012880 | gi|313880785|gb|AELG01001473.1| | 493 | 6927 | 7 | 2 |
| XLOC\_012887 XLOC\_012890 XLOC\_012889 | gi|313880799|gb|AELG01001459.1| | 6521 | 18451 | 27 | 1 |
| XLOC\_012888 | gi|313880799|gb|AELG01001459.1| | 62 | 4963 | 10 | 1 |
| XLOC\_012892 | gi|313880800|gb|AELG01001458.1| | 8262 | 18958 | 16 | 4 |
| XLOC\_012893 | gi|313880800|gb|AELG01001458.1| | 44 | 7628 | 12 | 2 |
| XLOC\_012898 XLOC\_012900 | gi|313880802|gb|AELG01001456.1| | 10899 | 32806 | 42 | 10 |
| XLOC\_012908 | gi|313880803|gb|AELG01001455.1| | 4077 | 18964 | 5 | 2 |
| XLOC\_012954 | gi|313880852|gb|AELG01001406.1| | 151 | 12058 | 14 | 1 |
| XLOC\_012957 | gi|313880852|gb|AELG01001406.1| | 36080 | 48082 | 13 | 3 |
| XLOC\_012958 XLOC\_012955 | gi|313880852|gb|AELG01001406.1| | 12515 | 29026 | 20 | 1 |
| XLOC\_012962 | gi|313880853|gb|AELG01001405.1| | 40095 | 53315 | 15 | 4 |
| XLOC\_012964 | gi|313880853|gb|AELG01001405.1| | 73300 | 80179 | 11 | 1 |
| XLOC\_012968 XLOC\_012965 | gi|313880853|gb|AELG01001405.1| | 97329 | 105878 | 20 | 2 |
| XLOC\_012985 | gi|313880854|gb|AELG01001404.1| | 10433 | 16507 | 11 | 1 |
| XLOC\_013000 XLOC\_013007 | gi|313880855|gb|AELG01001403.1| | 84909 | 108207 | 63 | 17 |
| XLOC\_013015 XLOC\_013016 | gi|313880856|gb|AELG01001402.1| | 5264 | 16851 | 22 | 1 |
| XLOC\_013021 XLOC\_013018 | gi|313880857|gb|AELG01001401.1| | 17249 | 22039 | 10 | 1 |
| XLOC\_013046 | gi|313880865|gb|AELG01001393.1| | 9174 | 77608 | 8 | 3 |
| XLOC\_013051 XLOC\_013054 | gi|313880868|gb|AELG01001390.1| | 61540 | 80447 | 20 | 1 |
| XLOC\_013056 | gi|313880868|gb|AELG01001390.1| | 180114 | 192194 | 12 | 1 |
| XLOC\_013070 XLOC\_013078 | gi|313880880|gb|AELG01001378.1| | 29506 | 41820 | 27 | 3 |
| XLOC\_013074 | gi|313880880|gb|AELG01001378.1| | 84021 | 100189 | 8 | 1 |
| XLOC\_013075 | gi|313880880|gb|AELG01001378.1| | 101260 | 110783 | 13 | 6 |
| XLOC\_013077 XLOC\_013076 XLOC\_013069 | gi|313880880|gb|AELG01001378.1| | 14554 | 27663 | 40 | 2 |
| XLOC\_013081 | gi|313880880|gb|AELG01001378.1| | 50307 | 74113 | 12 | 1 |
| XLOC\_013090 | gi|313880881|gb|AELG01001377.1| | 39133 | 54881 | 14 | 1 |
| XLOC\_013091 | gi|313880881|gb|AELG01001377.1| | 1 | 13012 | 18 | 4 |
| XLOC\_013096 XLOC\_013098 | gi|313880882|gb|AELG01001376.1| | 2449 | 17302 | 19 | 7 |
| XLOC\_013097 XLOC\_013100 | gi|313880882|gb|AELG01001376.1| | 19593 | 45798 | 28 | 2 |
| XLOC\_013136 | gi|313880948|gb|AELG01001310.1| | 49669 | 55004 | 14 | 1 |
| XLOC\_013138 | gi|313880948|gb|AELG01001310.1| | 66631 | 89179 | 26 | 3 |
| XLOC\_013143 | gi|313880952|gb|AELG01001307.1| | 52551 | 60436 | 10 | 2 |
| XLOC\_013144 XLOC\_013150 | gi|313880952|gb|AELG01001307.1| | 66459 | 83660 | 21 | 4 |
| XLOC\_013161 XLOC\_013166 | gi|313880953|gb|AELG01001306.1| | 51260 | 72900 | 31 | 1 |
| XLOC\_013162 | gi|313880953|gb|AELG01001306.1| | 650 | 12321 | 30 | 7 |
| XLOC\_013163 | gi|313880953|gb|AELG01001306.1| | 19908 | 42285 | 49 | 2 |
| XLOC\_013176 | gi|313880957|gb|AELG01001302.1| | 2112 | 10189 | 9 | 6 |
| XLOC\_013190 | gi|313880959|gb|AELG01001300.1| | 41319 | 44826 | 12 | 1 |
| XLOC\_013210 | gi|313880963|gb|AELG01001296.1| | 22940 | 36401 | 13 | 2 |
| XLOC\_013213 | gi|313880963|gb|AELG01001296.1| | 18233 | 22024 | 7 | 1 |
| XLOC\_013234 XLOC\_013236 XLOC\_013239 | gi|313880967|gb|AELG01001293.1| | 22068 | 43429 | 33 | 4 |
| XLOC\_013241 | gi|313880968|gb|AELG01001292.1| | 52144 | 75519 | 6 | 2 |
| XLOC\_013244 | gi|313880969|gb|AELG01001291.1| | 3879 | 17202 | 32 | 8 |
| XLOC\_013245 | gi|313880969|gb|AELG01001291.1| | 17503 | 45859 | 31 | 5 |
| XLOC\_013283 | gi|313880995|gb|AELG01001265.1| | 16 | 6329 | 13 | 2 |
| XLOC\_013286 | gi|313880995|gb|AELG01001265.1| | 19068 | 29248 | 14 | 5 |
| XLOC\_013288 XLOC\_013290 | gi|313880995|gb|AELG01001265.1| | 46105 | 62596 | 10 | 4 |
| XLOC\_013299 XLOC\_013297 | gi|313880996|gb|AELG01001264.1| | 6589 | 28777 | 17 | 1 |
| XLOC\_013300 | gi|313880996|gb|AELG01001264.1| | 7863 | 11968 | 12 | 1 |
| XLOC\_013309 XLOC\_013312 | gi|313881001|gb|AELG01001259.1| | 141485 | 147718 | 32 | 1 |
| XLOC\_013317 | gi|313881004|gb|AELG01001256.1| | 23664 | 33462 | 11 | 2 |
| XLOC\_013324 XLOC\_013330 XLOC\_013329 | gi|313881005|gb|AELG01001255.1| | 53651 | 75461 | 63 | 5 |
| XLOC\_013325 XLOC\_013331 | gi|313881005|gb|AELG01001255.1| | 76570 | 139524 | 29 | 4 |
| XLOC\_013328 | gi|313881005|gb|AELG01001255.1| | 44140 | 50178 | 14 | 1 |
| XLOC\_013337 XLOC\_013343 | gi|313881006|gb|AELG01001254.1| | 4605 | 39426 | 22 | 1 |
| XLOC\_013342 | gi|313881006|gb|AELG01001254.1| | 99 | 4308 | 12 | 1 |
| XLOC\_013344 XLOC\_013338 | gi|313881006|gb|AELG01001254.1| | 49119 | 57492 | 28 | 2 |
| XLOC\_013345 XLOC\_013339 | gi|313881006|gb|AELG01001254.1| | 58606 | 75006 | 19 | 1 |
| XLOC\_013351 | gi|313881007|gb|AELG01001253.1| | 79759 | 83528 | 10 | 1 |
| XLOC\_013355 | gi|313881007|gb|AELG01001253.1| | 95376 | 103088 | 3 | 2 |
| XLOC\_013357 | gi|313881007|gb|AELG01001253.1| | 107123 | 129897 | 11 | 2 |
| XLOC\_013358 | gi|313881007|gb|AELG01001253.1| | 10511 | 24238 | 26 | 2 |
| XLOC\_013381 XLOC\_013377 | gi|313881016|gb|AELG01001244.1| | 22907 | 34262 | 41 | 1 |
| XLOC\_013389 | gi|313881021|gb|AELG01001239.1| | 2581 | 24453 | 23 | 16 |
| XLOC\_013392 | gi|313881022|gb|AELG01001238.1| | 221 | 2876 | 8 | 2 |
| XLOC\_013401 | gi|313881023|gb|AELG01001237.1| | 29648 | 39948 | 13 | 2 |
| XLOC\_013409 | gi|313881043|gb|AELG01001217.1| | 17 | 4423 | 14 | 1 |
| XLOC\_013413 XLOC\_013414 | gi|313881056|gb|AELG01001204.1| | 83 | 5257 | 18 | 1 |
| XLOC\_013419 | gi|313881060|gb|AELG01001201.1| | 75475 | 81023 | 13 | 1 |
| XLOC\_013422 XLOC\_013423 | gi|313881061|gb|AELG01001200.1| | 4565 | 40044 | 38 | 1 |
| XLOC\_013436 | gi|313881063|gb|AELG01001198.1| | 2910 | 7875 | 16 | 2 |
| XLOC\_013440 | gi|313881064|gb|AELG01001197.1| | 118703 | 122630 | 11 | 1 |
| XLOC\_013441 XLOC\_013439 | gi|313881064|gb|AELG01001197.1| | 122699 | 128214 | 12 | 1 |
| XLOC\_013454 | gi|313881070|gb|AELG01001191.1| | 13540 | 17492 | 8 | 1 |
| XLOC\_013457 XLOC\_013465 | gi|313881070|gb|AELG01001191.1| | 38820 | 48231 | 23 | 3 |
| XLOC\_013462 | gi|313881070|gb|AELG01001191.1| | 2633 | 13475 | 9 | 1 |
| XLOC\_013468 XLOC\_013460 XLOC\_013467 XLOC\_013461 | gi|313881070|gb|AELG01001191.1| | 62014 | 72176 | 49 | 1 |
| XLOC\_013488 | gi|313881074|gb|AELG01001187.1| | 69513 | 78077 | 14 | 3 |
| XLOC\_013489 | gi|313881074|gb|AELG01001187.1| | 195 | 8045 | 22 | 3 |
| XLOC\_013493 XLOC\_013502 | gi|313881075|gb|AELG01001186.1| | 16409 | 45080 | 58 | 1 |
| XLOC\_013512 | gi|313881082|gb|AELG01001179.1| | 12761 | 24203 | 2 | 2 |
| XLOC\_013513 | gi|313881084|gb|AELG01001177.1| | 154885 | 216983 | 7 | 3 |
| XLOC\_013524 | gi|313881094|gb|AELG01001168.1| | 1 | 32052 | 13 | 3 |
| XLOC\_013527 | gi|313881096|gb|AELG01001166.1| | 2245 | 15892 | 15 | 4 |
| XLOC\_013529 XLOC\_013533 | gi|313881096|gb|AELG01001166.1| | 119380 | 134776 | 19 | 2 |
| XLOC\_013534 | gi|313881096|gb|AELG01001166.1| | 140975 | 149137 | 3 | 1 |
| XLOC\_013574 | gi|313881111|gb|AELG01001151.1| | 67137 | 83700 | 3 | 3 |
| XLOC\_013637 | gi|313881147|gb|AELG01001115.1| | 35388 | 38772 | 11 | 1 |
| XLOC\_013639 | gi|313881147|gb|AELG01001115.1| | 59190 | 64302 | 10 | 1 |
| XLOC\_013675 XLOC\_013674 | gi|313881151|gb|AELG01001111.1| | 29812 | 57723 | 22 | 3 |
| XLOC\_013682 | gi|313881154|gb|AELG01001108.1| | 18251 | 27363 | 7 | 1 |
| XLOC\_013713 XLOC\_013716 | gi|313881187|gb|AELG01001076.1| | 5266 | 9906 | 9 | 1 |
| XLOC\_013717 | gi|313881187|gb|AELG01001076.1| | 13639 | 19054 | 10 | 1 |
| XLOC\_013733 | gi|313881191|gb|AELG01001072.1| | 18843 | 22976 | 17 | 2 |
| XLOC\_013739 XLOC\_013734 | gi|313881192|gb|AELG01001071.1| | 1677 | 5081 | 9 | 1 |
| XLOC\_013742 XLOC\_013736 | gi|313881192|gb|AELG01001071.1| | 15579 | 40295 | 46 | 3 |
| XLOC\_013775 | gi|313881200|gb|AELG01001063.1| | 9266 | 22103 | 20 | 4 |
| XLOC\_013777 | gi|313881200|gb|AELG01001063.1| | 32591 | 41058 | 21 | 1 |
| XLOC\_013792 | gi|313881201|gb|AELG01001062.1| | 11739 | 18089 | 11 | 3 |
| XLOC\_013815 XLOC\_013814 XLOC\_013824 | gi|313881210|gb|AELG01001054.1| | 95993 | 108527 | 35 | 1 |
| XLOC\_013817 XLOC\_013825 | gi|313881210|gb|AELG01001054.1| | 111712 | 147362 | 19 | 1 |
| XLOC\_013818 XLOC\_013827 | gi|313881210|gb|AELG01001054.1| | 1450 | 6762 | 4 | 1 |
| XLOC\_013819 | gi|313881210|gb|AELG01001054.1| | 7384 | 15926 | 20 | 5 |
| XLOC\_013820 XLOC\_013821 XLOC\_013812 | gi|313881210|gb|AELG01001054.1| | 16743 | 55761 | 49 | 1 |
| XLOC\_013830 | gi|313881216|gb|AELG01001048.1| | 1859 | 5476 | 3 | 1 |
| XLOC\_013837 | gi|313881217|gb|AELG01001047.1| | 8380 | 16359 | 10 | 1 |
| XLOC\_013842 | gi|313881218|gb|AELG01001046.1| | 35493 | 40412 | 5 | 2 |
| XLOC\_013845 XLOC\_013855 | gi|313881218|gb|AELG01001046.1| | 67291 | 76509 | 25 | 4 |
| XLOC\_013848 | gi|313881218|gb|AELG01001046.1| | 154 | 8076 | 7 | 2 |
| XLOC\_013853 | gi|313881218|gb|AELG01001046.1| | 41431 | 57056 | 9 | 4 |
| XLOC\_013863 | gi|313881219|gb|AELG01001045.1| | 13196 | 24091 | 10 | 1 |
| XLOC\_013870 | gi|313881220|gb|AELG01001044.1| | 5004 | 18611 | 11 | 2 |
| XLOC\_013877 | gi|313881221|gb|AELG01001043.1| | 392698 | 417950 | 26 | 2 |
| XLOC\_013889 XLOC\_013890 XLOC\_013872 | gi|313881221|gb|AELG01001043.1| | 357108 | 372279 | 47 | 2 |
| XLOC\_013895 XLOC\_013894 XLOC\_013880 XLOC\_013881 | gi|313881221|gb|AELG01001043.1| | 430388 | 445999 | 34 | 1 |
| XLOC\_013914 | gi|313881235|gb|AELG01001031.1| | 52833 | 65052 | 18 | 3 |
| XLOC\_013917 XLOC\_013922 | gi|313881235|gb|AELG01001031.1| | 70124 | 81547 | 24 | 2 |
| XLOC\_013921 | gi|313881235|gb|AELG01001031.1| | 26624 | 52053 | 11 | 1 |
| XLOC\_013952 | gi|313881245|gb|AELG01001021.1| | 11005 | 34884 | 18 | 2 |
| XLOC\_013956 | gi|313881246|gb|AELG01001020.1| | 35581 | 66831 | 45 | 2 |
| XLOC\_013957 | gi|313881246|gb|AELG01001020.1| | 68981 | 79177 | 10 | 2 |
| XLOC\_013973 | gi|313881247|gb|AELG01001019.1| | 23578 | 34132 | 15 | 2 |
| XLOC\_013974 XLOC\_013971 | gi|313881247|gb|AELG01001019.1| | 14698 | 19700 | 12 | 1 |
| XLOC\_013980 | gi|313881251|gb|AELG01001015.1| | 41214 | 47070 | 6 | 1 |
| XLOC\_013987 XLOC\_013986 | gi|313881252|gb|AELG01001014.1| | 17652 | 64836 | 42 | 7 |
| XLOC\_013998 | gi|313881256|gb|AELG01001010.1| | 42752 | 58254 | 13 | 3 |
| XLOC\_014003 XLOC\_014002 XLOC\_014009 | gi|313881256|gb|AELG01001010.1| | 96799 | 116109 | 27 | 2 |
| XLOC\_014005 XLOC\_013996 | gi|313881256|gb|AELG01001010.1| | 20967 | 26585 | 22 | 1 |
| XLOC\_014006 | gi|313881256|gb|AELG01001010.1| | 27341 | 31409 | 16 | 1 |
| XLOC\_014024 | gi|313881259|gb|AELG01001008.1| | 11518 | 17426 | 15 | 2 |
| XLOC\_014039 | gi|313881260|gb|AELG01001007.1| | 78382 | 97174 | 13 | 5 |
| XLOC\_014058 | gi|313881268|gb|AELG01000999.1| | 32360 | 47717 | 18 | 1 |
| XLOC\_014063 | gi|313881268|gb|AELG01000999.1| | 1 | 5948 | 18 | 2 |
| XLOC\_014066 XLOC\_014057 | gi|313881268|gb|AELG01000999.1| | 17568 | 26128 | 22 | 1 |
| XLOC\_014076 | gi|313881269|gb|AELG01000998.1| | 6322 | 10195 | 10 | 1 |
| XLOC\_014083 XLOC\_014082 XLOC\_014080 | gi|313881270|gb|AELG01000997.1| | 10462 | 24983 | 38 | 1 |
| XLOC\_014102 XLOC\_014109 | gi|313881272|gb|AELG01000995.1| | 10079 | 36202 | 76 | 1 |
| XLOC\_014112 | gi|313881272|gb|AELG01000995.1| | 67686 | 71971 | 7 | 1 |
| XLOC\_014118 XLOC\_014115 | gi|313881273|gb|AELG01000994.1| | 23057 | 40738 | 18 | 5 |
| XLOC\_014121 XLOC\_014116 | gi|313881273|gb|AELG01000994.1| | 59710 | 98412 | 44 | 7 |
| XLOC\_014130 | gi|313881274|gb|AELG01000993.1| | 6049 | 10189 | 14 | 1 |
| XLOC\_014131 | gi|313881275|gb|AELG01000992.1| | 151 | 4209 | 2 | 2 |
| XLOC\_014139 | gi|313881275|gb|AELG01000992.1| | 37765 | 47153 | 12 | 2 |
| XLOC\_014142 | gi|313881275|gb|AELG01000992.1| | 17540 | 24442 | 13 | 8 |
| XLOC\_014148 | gi|313881276|gb|AELG01000991.1| | 61623 | 66438 | 8 | 2 |
| XLOC\_014164 | gi|313881278|gb|AELG01000989.1| | 104840 | 141894 | 8 | 1 |
| XLOC\_014171 | gi|313881279|gb|AELG01000988.1| | 5911 | 32502 | 11 | 2 |
| XLOC\_014172 | gi|313881280|gb|AELG01000987.1| | 18910 | 55326 | 15 | 2 |
| XLOC\_014173 XLOC\_014178 | gi|313881280|gb|AELG01000987.1| | 55770 | 75516 | 45 | 1 |
| XLOC\_014174 XLOC\_014180 XLOC\_014181 | gi|313881280|gb|AELG01000987.1| | 84640 | 102084 | 60 | 6 |
| XLOC\_014179 XLOC\_014185 | gi|313881280|gb|AELG01000987.1| | 78651 | 84497 | 10 | 2 |
| XLOC\_014189 | gi|313881281|gb|AELG01000986.1| | 9059 | 16649 | 5 | 4 |
| XLOC\_014195 XLOC\_014194 | gi|313881282|gb|AELG01000985.1| | 16580 | 21792 | 14 | 1 |
| XLOC\_014212 | gi|313881315|gb|AELG01000978.1| | 14543 | 36709 | 56 | 29 |
| XLOC\_014222 XLOC\_014233 | gi|313881316|gb|AELG01000977.1| | 87449 | 99893 | 24 | 1 |
| XLOC\_014225 | gi|313881316|gb|AELG01000977.1| | 120663 | 127246 | 14 | 1 |
| XLOC\_014230 XLOC\_014231 XLOC\_014220 | gi|313881316|gb|AELG01000977.1| | 40230 | 62959 | 53 | 1 |
| XLOC\_014235 | gi|313881316|gb|AELG01000977.1| | 127325 | 130818 | 3 | 2 |
| XLOC\_014236 | gi|313881316|gb|AELG01000977.1| | 164791 | 202627 | 21 | 6 |
| XLOC\_014238 XLOC\_014246 | gi|313881316|gb|AELG01000977.1| | 207825 | 220536 | 4 | 3 |
| XLOC\_014260 | gi|313881322|gb|AELG01000971.1| | 37968 | 57515 | 15 | 6 |
| XLOC\_014262 | gi|313881323|gb|AELG01000970.1| | 34 | 17827 | 9 | 2 |
| XLOC\_014276 XLOC\_014289 | gi|313881324|gb|AELG01000969.1| | 223681 | 279173 | 31 | 5 |
| XLOC\_014279 XLOC\_014268 | gi|313881324|gb|AELG01000969.1| | 83495 | 104713 | 30 | 4 |
| XLOC\_014280 | gi|313881324|gb|AELG01000969.1| | 106398 | 115261 | 7 | 4 |
| XLOC\_014287 XLOC\_014273 | gi|313881324|gb|AELG01000969.1| | 148837 | 194640 | 49 | 12 |
| XLOC\_014310 | gi|313881330|gb|AELG01000963.1| | 90193 | 105686 | 32 | 5 |
| XLOC\_014332 XLOC\_014329 | gi|313881335|gb|AELG01000960.1| | 34492 | 105599 | 20 | 9 |
| XLOC\_014348 XLOC\_014349 XLOC\_014352 XLOC\_014351 | gi|313881338|gb|AELG01000957.1| | 36624 | 86407 | 43 | 2 |
| XLOC\_014367 | gi|313881347|gb|AELG01000948.1| | 540 | 20144 | 37 | 18 |
| XLOC\_014371 | gi|313881347|gb|AELG01000948.1| | 31119 | 40636 | 4 | 3 |
| XLOC\_014382 | gi|313881348|gb|AELG01000947.1| | 239869 | 244691 | 14 | 1 |
| XLOC\_014384 | gi|313881348|gb|AELG01000947.1| | 180711 | 184608 | 10 | 2 |
| XLOC\_014400 | gi|313881359|gb|AELG01000936.1| | 4551 | 10666 | 14 | 1 |
| XLOC\_014418 XLOC\_014427 | gi|313881361|gb|AELG01000934.1| | 88235 | 95512 | 19 | 1 |
| XLOC\_014429 XLOC\_014420 | gi|313881361|gb|AELG01000934.1| | 99247 | 118785 | 45 | 13 |
| XLOC\_014432 | gi|313881361|gb|AELG01000934.1| | 129362 | 136464 | 14 | 1 |
| XLOC\_014433 XLOC\_014422 | gi|313881361|gb|AELG01000934.1| | 137712 | 144385 | 18 | 2 |
| XLOC\_014452 | gi|313881375|gb|AELG01000920.1| | 2419 | 56691 | 45 | 2 |
| XLOC\_014466 | gi|313881385|gb|AELG01000912.1| | 20049 | 28794 | 12 | 1 |
| XLOC\_014517 XLOC\_014513 | gi|313881418|gb|AELG01000880.1| | 55635 | 63757 | 14 | 7 |
| XLOC\_014521 | gi|313881419|gb|AELG01000879.1| | 24096 | 28593 | 12 | 1 |
| XLOC\_014529 XLOC\_014530 | gi|313881421|gb|AELG01000877.1| | 18 | 25322 | 24 | 2 |
| XLOC\_014533 XLOC\_014532 | gi|313881422|gb|AELG01000876.1| | 24 | 20892 | 18 | 2 |
| XLOC\_014534 | gi|313881423|gb|AELG01000875.1| | 2214 | 4684 | 11 | 1 |
| XLOC\_014550 XLOC\_014545 | gi|313881431|gb|AELG01000869.1| | 2244 | 53395 | 26 | 5 |
| XLOC\_014568 | gi|313881434|gb|AELG01000866.1| | 27939 | 35879 | 11 | 1 |
| XLOC\_014573 | gi|313881434|gb|AELG01000866.1| | 25080 | 27692 | 9 | 1 |
| XLOC\_014594 | gi|313881436|gb|AELG01000864.1| | 3922 | 12383 | 8 | 2 |
| XLOC\_014598 | gi|313881436|gb|AELG01000864.1| | 77883 | 82985 | 17 | 3 |
| XLOC\_014618 | gi|313881436|gb|AELG01000864.1| | 165607 | 170278 | 5 | 1 |
| XLOC\_014630 XLOC\_014638 | gi|313881437|gb|AELG01000863.1| | 10498 | 19597 | 27 | 4 |
| XLOC\_014631 XLOC\_014632 XLOC\_014640 | gi|313881437|gb|AELG01000863.1| | 22855 | 48162 | 41 | 1 |
| XLOC\_014641 | gi|313881437|gb|AELG01000863.1| | 91839 | 100297 | 16 | 1 |
| XLOC\_014653 | gi|313881438|gb|AELG01000862.1| | 35854 | 38922 | 9 | 1 |
| XLOC\_014672 XLOC\_014675 | gi|313881443|gb|AELG01000857.1| | 44 | 8024 | 17 | 2 |
| XLOC\_014681 XLOC\_014687 | gi|313881447|gb|AELG01000853.1| | 31464 | 72590 | 30 | 3 |
| XLOC\_014684 XLOC\_014690 | gi|313881447|gb|AELG01000853.1| | 1 | 9507 | 21 | 2 |
| XLOC\_014685 | gi|313881447|gb|AELG01000853.1| | 10640 | 25995 | 6 | 1 |
| XLOC\_014696 | gi|313881448|gb|AELG01000852.1| | 34027 | 37505 | 12 | 1 |
| XLOC\_014701 XLOC\_014714 | gi|313881448|gb|AELG01000852.1| | 81811 | 95971 | 26 | 1 |
| XLOC\_014702 XLOC\_014715 | gi|313881448|gb|AELG01000852.1| | 92229 | 103130 | 47 | 1 |
| XLOC\_014735 | gi|313881453|gb|AELG01000847.1| | 39273 | 45736 | 12 | 1 |
| XLOC\_014742 | gi|313881453|gb|AELG01000847.1| | 63899 | 68570 | 8 | 2 |
| XLOC\_014750 | gi|313881454|gb|AELG01000846.1| | 1635 | 9232 | 6 | 1 |
| XLOC\_014755 XLOC\_014762 | gi|313881463|gb|AELG01000837.1| | 38521 | 47397 | 28 | 1 |
| XLOC\_014767 XLOC\_014763 XLOC\_014756 XLOC\_014768 | gi|313881463|gb|AELG01000837.1| | 50374 | 76112 | 30 | 1 |
| XLOC\_014777 | gi|313881464|gb|AELG01000836.1| | 119772 | 126324 | 20 | 2 |
| XLOC\_014780 | gi|313881464|gb|AELG01000836.1| | 19422 | 62118 | 19 | 5 |
| XLOC\_014786 | gi|313881464|gb|AELG01000836.1| | 139603 | 159971 | 9 | 4 |
| XLOC\_014798 | gi|313881466|gb|AELG01000834.1| | 66965 | 72970 | 13 | 2 |
| XLOC\_014799 | gi|313881466|gb|AELG01000834.1| | 116662 | 132479 | 20 | 2 |
| XLOC\_014837 XLOC\_014831 | gi|313881474|gb|AELG01000826.1| | 7469 | 36545 | 60 | 16 |
| XLOC\_014841 | gi|313881474|gb|AELG01000826.1| | 71189 | 128297 | 25 | 1 |
| XLOC\_014842 XLOC\_014833 | gi|313881474|gb|AELG01000826.1| | 131136 | 135259 | 24 | 1 |
| XLOC\_014859 XLOC\_014864 | gi|313881475|gb|AELG01000825.1| | 24374 | 32957 | 20 | 5 |
| XLOC\_014895 | gi|313881504|gb|AELG01000796.1| | 25 | 7231 | 8 | 2 |
| XLOC\_014913 | gi|313881514|gb|AELG01000786.1| | 48831 | 57673 | 6 | 1 |
| XLOC\_014920 | gi|313881514|gb|AELG01000786.1| | 104845 | 111985 | 8 | 1 |
| XLOC\_014941 | gi|313881524|gb|AELG01000776.1| | 31150 | 56696 | 8 | 5 |
| XLOC\_014950 | gi|313881529|gb|AELG01000771.1| | 57930 | 80038 | 47 | 2 |
| XLOC\_014958 | gi|313881529|gb|AELG01000771.1| | 4813 | 16266 | 15 | 1 |
| XLOC\_014964 XLOC\_014953 | gi|313881529|gb|AELG01000771.1| | 109989 | 122192 | 29 | 6 |
| XLOC\_014986 | gi|313881530|gb|AELG01000770.1| | 40253 | 54782 | 36 | 2 |
| XLOC\_014990 XLOC\_014985 XLOC\_014989 | gi|313881530|gb|AELG01000770.1| | 22831 | 39616 | 59 | 4 |
| XLOC\_015004 | gi|313881531|gb|AELG01000769.1| | 15727 | 37778 | 16 | 3 |
| XLOC\_015005 | gi|313881531|gb|AELG01000769.1| | 40867 | 53253 | 16 | 5 |
| XLOC\_015009 | gi|313881532|gb|AELG01000768.1| | 22613 | 47023 | 47 | 6 |
| XLOC\_015031 XLOC\_015030 XLOC\_015035 | gi|313881535|gb|AELG01000765.1| | 21174 | 36959 | 26 | 1 |
| XLOC\_015032 | gi|313881535|gb|AELG01000765.1| | 45213 | 50501 | 5 | 2 |
| XLOC\_015034 | gi|313881535|gb|AELG01000765.1| | 4900 | 17496 | 19 | 4 |
| XLOC\_015036 | gi|313881535|gb|AELG01000765.1| | 38084 | 44489 | 16 | 1 |
| XLOC\_015039 XLOC\_015043 | gi|313881536|gb|AELG01000764.1| | 47776 | 55082 | 38 | 1 |
| XLOC\_015067 | gi|313881547|gb|AELG01000753.1| | 1022 | 11925 | 13 | 3 |
| XLOC\_015091 | gi|313881549|gb|AELG01000751.1| | 38029 | 63941 | 24 | 1 |
| XLOC\_015098 | gi|313881550|gb|AELG01000750.1| | 9499 | 16233 | 9 | 1 |
| XLOC\_015104 XLOC\_015107 | gi|313881551|gb|AELG01000749.1| | 8 | 29143 | 45 | 1 |
| XLOC\_015113 XLOC\_015116 | gi|313881553|gb|AELG01000747.1| | 42923 | 84376 | 40 | 1 |
| XLOC\_015122 XLOC\_015121 | gi|313881554|gb|AELG01000746.1| | 189 | 13276 | 22 | 1 |
| XLOC\_015123 | gi|313881554|gb|AELG01000746.1| | 16780 | 27795 | 9 | 7 |
| XLOC\_015143 | gi|313881557|gb|AELG01000743.1| | 45778 | 53663 | 17 | 3 |
| XLOC\_015151 | gi|313881565|gb|AELG01000735.1| | 10717 | 16575 | 16 | 1 |
| XLOC\_015154 | gi|313881566|gb|AELG01000734.1| | 1997 | 24416 | 6 | 1 |
| XLOC\_015161 | gi|313881571|gb|AELG01000729.1| | 18733 | 26806 | 4 | 1 |
| XLOC\_015170 | gi|313881572|gb|AELG01000728.1| | 64774 | 69385 | 20 | 1 |
| XLOC\_015181 | gi|313881572|gb|AELG01000728.1| | 28843 | 32398 | 5 | 1 |
| XLOC\_015193 XLOC\_015180 | gi|313881572|gb|AELG01000728.1| | 18202 | 25725 | 19 | 3 |
| XLOC\_015196 | gi|313881573|gb|AELG01000727.1| | 645 | 8325 | 7 | 2 |
| XLOC\_015213 | gi|313881577|gb|AELG01000723.1| | 38064 | 57498 | 10 | 1 |
| XLOC\_015231 | gi|313881580|gb|AELG01000720.1| | 17498 | 43333 | 12 | 1 |
| XLOC\_015234 | gi|313881582|gb|AELG01000718.1| | 25039 | 42785 | 14 | 1 |
| XLOC\_015240 XLOC\_015238 | gi|313881584|gb|AELG01000716.1| | 69279 | 97820 | 20 | 1 |
| XLOC\_015268 | gi|313881587|gb|AELG01000713.1| | 84937 | 114623 | 26 | 9 |
| XLOC\_015284 | gi|313881589|gb|AELG01000711.1| | 2332 | 6501 | 11 | 1 |
| XLOC\_015288 | gi|313881590|gb|AELG01000710.1| | 5278 | 7335 | 5 | 1 |
| XLOC\_015307 XLOC\_015304 | gi|313881598|gb|AELG01000702.1| | 8703 | 17491 | 19 | 5 |
| XLOC\_015321 | gi|313881600|gb|AELG01000700.1| | 23920 | 71037 | 22 | 1 |
| XLOC\_015322 | gi|313881600|gb|AELG01000700.1| | 83491 | 96434 | 7 | 1 |
| XLOC\_015340 XLOC\_015339 | gi|313881607|gb|AELG01000693.1| | 28467 | 78533 | 9 | 2 |
| XLOC\_015344 | gi|313881612|gb|AELG01000688.1| | 80925 | 87508 | 6 | 1 |
| XLOC\_015353 | gi|313881614|gb|AELG01000686.1| | 17325 | 22462 | 13 | 1 |
| XLOC\_015355 | gi|313881614|gb|AELG01000686.1| | 1547 | 16549 | 6 | 3 |
| XLOC\_015376 XLOC\_015380 | gi|313881619|gb|AELG01000681.1| | 21582 | 31033 | 29 | 2 |
| XLOC\_015377 | gi|313881619|gb|AELG01000681.1| | 31654 | 102909 | 24 | 1 |
| XLOC\_015397 | gi|313881621|gb|AELG01000679.1| | 27416 | 37147 | 10 | 2 |
| XLOC\_015400 XLOC\_015398 | gi|313881621|gb|AELG01000679.1| | 45218 | 52952 | 45 | 1 |
| XLOC\_015411 XLOC\_015407 | gi|313881622|gb|AELG01000678.1| | 11059 | 99241 | 38 | 2 |
| XLOC\_015428 XLOC\_015438 | gi|313881624|gb|AELG01000676.1| | 148810 | 154763 | 19 | 1 |
| XLOC\_015429 | gi|313881624|gb|AELG01000676.1| | 155122 | 163443 | 14 | 2 |
| XLOC\_015441 | gi|313881624|gb|AELG01000676.1| | 190187 | 231574 | 23 | 1 |
| XLOC\_015444 XLOC\_015447 | gi|313881625|gb|AELG01000675.1| | 8033 | 19598 | 35 | 1 |
| XLOC\_015448 | gi|313881625|gb|AELG01000675.1| | 27498 | 88433 | 10 | 1 |
| XLOC\_015456 | gi|313881627|gb|AELG01000673.1| | 4963 | 103460 | 12 | 2 |
| XLOC\_015457 | gi|313881627|gb|AELG01000673.1| | 105810 | 185678 | 7 | 3 |
| XLOC\_015477 | gi|313881640|gb|AELG01000660.1| | 6048 | 16373 | 15 | 2 |
| XLOC\_015479 | gi|313881640|gb|AELG01000660.1| | 126 | 4704 | 11 | 2 |
| XLOC\_015491 XLOC\_015487 | gi|313881641|gb|AELG01000659.1| | 13254 | 26615 | 38 | 1 |
| XLOC\_015503 XLOC\_015496 | gi|313881642|gb|AELG01000658.1| | 71310 | 85697 | 32 | 1 |
| XLOC\_015504 | gi|313881642|gb|AELG01000658.1| | 88245 | 90062 | 6 | 1 |
| XLOC\_015527 XLOC\_015525 | gi|313881651|gb|AELG01000649.1| | 44362 | 55136 | 33 | 1 |
| XLOC\_015539 | gi|313881657|gb|AELG01000643.1| | 10581 | 19133 | 9 | 3 |
| XLOC\_015547 XLOC\_015546 XLOC\_015541 XLOC\_015540 | gi|313881657|gb|AELG01000643.1| | 19247 | 50874 | 66 | 8 |
| XLOC\_015548 | gi|313881657|gb|AELG01000643.1| | 57415 | 64594 | 18 | 1 |
| XLOC\_015557 | gi|313881661|gb|AELG01000639.1| | 66181 | 80513 | 9 | 3 |
| XLOC\_015561 | gi|313881661|gb|AELG01000639.1| | 40704 | 50317 | 7 | 1 |
| XLOC\_015571 XLOC\_015574 | gi|313881671|gb|AELG01000629.1| | 4657 | 7739 | 12 | 1 |
| XLOC\_015572 | gi|313881671|gb|AELG01000629.1| | 8232 | 14536 | 12 | 3 |
| XLOC\_015589 | gi|313881672|gb|AELG01000628.1| | 157733 | 166062 | 10 | 1 |
| XLOC\_015590 | gi|313881672|gb|AELG01000628.1| | 167586 | 174941 | 11 | 3 |
| XLOC\_015597 XLOC\_015580 | gi|313881672|gb|AELG01000628.1| | 27902 | 36465 | 37 | 1 |
| XLOC\_015598 | gi|313881672|gb|AELG01000628.1| | 37296 | 39450 | 6 | 1 |
| XLOC\_015603 XLOC\_015586 | gi|313881672|gb|AELG01000628.1| | 102518 | 115380 | 53 | 1 |
| XLOC\_015619 XLOC\_015618 | gi|313881679|gb|AELG01000621.1| | 5989 | 16762 | 26 | 2 |
| XLOC\_015630 | gi|313881680|gb|AELG01000620.1| | 42247 | 48822 | 6 | 1 |
| XLOC\_015635 | gi|313881681|gb|AELG01000619.1| | 938 | 16273 | 9 | 2 |
| XLOC\_015642 | gi|313881684|gb|AELG01000616.1| | 6597 | 20926 | 4 | 2 |
| XLOC\_015652 | gi|313881685|gb|AELG01000615.1| | 43634 | 70722 | 10 | 3 |
| XLOC\_015706 | gi|313881730|gb|AELG01000570.1| | 15237 | 22867 | 6 | 2 |
| XLOC\_015709 | gi|313881735|gb|AELG01000565.1| | 2745 | 24888 | 20 | 1 |
| XLOC\_015735 | gi|313881751|gb|AELG01000549.1| | 4558 | 53600 | 11 | 2 |
| XLOC\_015755 XLOC\_015758 | gi|313881757|gb|AELG01000543.1| | 57098 | 65374 | 7 | 1 |
| XLOC\_015785 | gi|313881761|gb|AELG01000539.1| | 25438 | 29552 | 5 | 1 |
| XLOC\_015787 | gi|313881761|gb|AELG01000539.1| | 45142 | 50325 | 8 | 1 |
| XLOC\_015791 XLOC\_015786 | gi|313881761|gb|AELG01000539.1| | 30265 | 44684 | 41 | 2 |
| XLOC\_015797 | gi|313881762|gb|AELG01000538.1| | 94663 | 102808 | 9 | 2 |
| XLOC\_015835 | gi|313881797|gb|AELG01000503.1| | 26596 | 51740 | 31 | 10 |
| XLOC\_015895 XLOC\_015894 | gi|313881871|gb|AELG01000429.1| | 1197 | 8277 | 10 | 1 |
| XLOC\_015901 XLOC\_015900 | gi|313881872|gb|AELG01000428.1| | 72654 | 93109 | 40 | 1 |
| XLOC\_015934 | gi|313881896|gb|AELG01000404.1| | 56464 | 70875 | 10 | 1 |
| XLOC\_015938 | gi|313881897|gb|AELG01000403.1| | 29363 | 33962 | 7 | 1 |
| XLOC\_015952 | gi|313881935|gb|AELG01000365.1| | 3555 | 6081 | 11 | 1 |
| XLOC\_015964 | gi|313881938|gb|AELG01000362.1| | 14448 | 22527 | 20 | 2 |
| XLOC\_015970 XLOC\_015972 | gi|313881939|gb|AELG01000361.1| | 7852 | 23431 | 20 | 1 |
| XLOC\_016003 | gi|313881944|gb|AELG01000356.1| | 7946 | 13576 | 17 | 1 |
| XLOC\_016008 | gi|313881946|gb|AELG01000354.1| | 8 | 41081 | 21 | 1 |
| XLOC\_016009 | gi|313881947|gb|AELG01000353.1| | 31129 | 41440 | 5 | 1 |
| XLOC\_016059 | gi|313881978|gb|AELG01000322.1| | 35990 | 40368 | 12 | 1 |
| XLOC\_016061 XLOC\_016060 XLOC\_016055 | gi|313881978|gb|AELG01000322.1| | 41948 | 45890 | 20 | 2 |
| XLOC\_016065 | gi|313881982|gb|AELG01000318.1| | 27243 | 37350 | 5 | 1 |
| XLOC\_016096 | gi|313881993|gb|AELG01000307.1| | 32639 | 43236 | 10 | 3 |
| XLOC\_016106 | gi|313881994|gb|AELG01000306.1| | 2438 | 11237 | 17 | 4 |
| XLOC\_016114 XLOC\_016107 | gi|313881995|gb|AELG01000305.1| | 405 | 8204 | 29 | 1 |
| XLOC\_016119 | gi|313881996|gb|AELG01000304.1| | 41777 | 59020 | 16 | 3 |
| XLOC\_016138 XLOC\_016132 | gi|313881997|gb|AELG01000303.1| | 27628 | 44777 | 50 | 1 |
| XLOC\_016184 XLOC\_016185 | gi|313882005|gb|AELG01000295.1| | 7209 | 26688 | 10 | 3 |
| XLOC\_016237 | gi|313882023|gb|AELG01000277.1| | 78792 | 84798 | 19 | 1 |
| XLOC\_016245 | gi|313882029|gb|AELG01000271.1| | 13083 | 60699 | 6 | 4 |
| XLOC\_016259 | gi|313882043|gb|AELG01000257.1| | 39357 | 81506 | 14 | 2 |
| XLOC\_016270 | gi|313882046|gb|AELG01000254.1| | 56 | 15455 | 18 | 1 |
| XLOC\_016273 | gi|313882046|gb|AELG01000254.1| | 72589 | 76654 | 5 | 1 |
| XLOC\_016274 XLOC\_016271 | gi|313882046|gb|AELG01000254.1| | 16981 | 68843 | 16 | 1 |
| XLOC\_016281 | gi|313882059|gb|AELG01000241.1| | 17410 | 31444 | 7 | 2 |
| XLOC\_016283 | gi|313882059|gb|AELG01000241.1| | 61651 | 64367 | 8 | 1 |
| XLOC\_016289 XLOC\_016293 XLOC\_016294 XLOC\_016296 | gi|313882060|gb|AELG01000240.1| | 67369 | 78016 | 27 | 5 |
| XLOC\_016292 XLOC\_016287 | gi|313882060|gb|AELG01000240.1| | 42723 | 54692 | 28 | 1 |
| XLOC\_016298 | gi|313882067|gb|AELG01000233.1| | 69249 | 83714 | 5 | 1 |
| XLOC\_016317 | gi|313882069|gb|AELG01000231.1| | 29015 | 75721 | 36 | 8 |
| XLOC\_016320 XLOC\_016315 XLOC\_016319 | gi|313882069|gb|AELG01000231.1| | 8959 | 17454 | 41 | 1 |
| XLOC\_016326 | gi|313882069|gb|AELG01000231.1| | 82523 | 87597 | 3 | 2 |
| XLOC\_016335 XLOC\_016334 | gi|313882072|gb|AELG01000228.1| | 10519 | 100554 | 26 | 4 |
| XLOC\_016340 | gi|313882075|gb|AELG01000225.1| | 20778 | 26424 | 10 | 1 |
| XLOC\_016349 | gi|313882080|gb|AELG01000220.1| | 1667 | 5362 | 5 | 1 |
| XLOC\_016353 | gi|313882083|gb|AELG01000217.1| | 162 | 22495 | 11 | 1 |
| XLOC\_016355 | gi|313882086|gb|AELG01000214.1| | 6852 | 31288 | 12 | 1 |
| XLOC\_016373 XLOC\_016372 | gi|313882112|gb|AELG01000188.1| | 84822 | 107824 | 25 | 2 |
| XLOC\_016380 | gi|313882114|gb|AELG01000186.1| | 53260 | 58653 | 10 | 2 |
| XLOC\_016391 XLOC\_016392 | gi|313882123|gb|AELG01000177.1| | 24526 | 26674 | 13 | 1 |
| XLOC\_016401 XLOC\_016403 | gi|313882143|gb|AELG01000157.1| | 36385 | 44540 | 29 | 1 |
| XLOC\_016402 | gi|313882143|gb|AELG01000157.1| | 6 | 24072 | 5 | 1 |
| XLOC\_016417 | gi|313882144|gb|AELG01000156.1| | 103371 | 139415 | 13 | 1 |
| XLOC\_016419 | gi|313882144|gb|AELG01000156.1| | 152324 | 165940 | 7 | 3 |
| XLOC\_016445 | gi|313882149|gb|AELG01000151.1| | 61378 | 66499 | 7 | 1 |
| XLOC\_016458 | gi|313882152|gb|AELG01000148.1| | 16369 | 25688 | 8 | 2 |
| XLOC\_016466 | gi|313882165|gb|AELG01000135.1| | 3627 | 10189 | 18 | 1 |
| XLOC\_016477 XLOC\_016469 | gi|313882166|gb|AELG01000134.1| | 25600 | 31611 | 12 | 1 |
| XLOC\_016498 | gi|313882169|gb|AELG01000131.1| | 45452 | 61978 | 30 | 5 |
| XLOC\_016500 | gi|313882169|gb|AELG01000131.1| | 86334 | 96614 | 34 | 4 |
| XLOC\_016505 XLOC\_016497 | gi|313882169|gb|AELG01000131.1| | 32920 | 44400 | 48 | 2 |
| XLOC\_016510 | gi|313882170|gb|AELG01000130.1| | 7328 | 23080 | 26 | 2 |
| XLOC\_016513 XLOC\_016512 XLOC\_016519 XLOC\_016518 | gi|313882171|gb|AELG01000129.1| | 12255 | 36538 | 60 | 2 |
| XLOC\_016514 XLOC\_016521 | gi|313882171|gb|AELG01000129.1| | 52316 | 78939 | 21 | 6 |
| XLOC\_016516 | gi|313882171|gb|AELG01000129.1| | 91560 | 97481 | 8 | 1 |
| XLOC\_016517 XLOC\_016524 | gi|313882171|gb|AELG01000129.1| | 101455 | 107140 | 15 | 2 |
| XLOC\_016536 | gi|313882173|gb|AELG01000127.1| | 10420 | 15355 | 12 | 1 |
| XLOC\_016540 | gi|313882173|gb|AELG01000127.1| | 15562 | 22327 | 10 | 1 |
| XLOC\_016545 | gi|313882174|gb|AELG01000126.1| | 25549 | 33308 | 9 | 5 |
| XLOC\_016546 | gi|313882174|gb|AELG01000126.1| | 5 | 6782 | 16 | 1 |
| XLOC\_016552 | gi|313882175|gb|AELG01000125.1| | 20582 | 28801 | 13 | 1 |
| XLOC\_016556 | gi|313882175|gb|AELG01000125.1| | 78320 | 93272 | 12 | 1 |
| XLOC\_016558 | gi|313882175|gb|AELG01000125.1| | 108465 | 119822 | 20 | 1 |
| XLOC\_016564 | gi|313882175|gb|AELG01000125.1| | 34493 | 44342 | 6 | 1 |
| XLOC\_016574 XLOC\_016566 | gi|313882175|gb|AELG01000125.1| | 53272 | 75067 | 27 | 7 |
| XLOC\_016604 XLOC\_016602 | gi|313882178|gb|AELG01000122.1| | 42963 | 55607 | 41 | 1 |
| XLOC\_016628 | gi|313882193|gb|AELG01000107.1| | 117683 | 125232 | 11 | 1 |
| XLOC\_016643 | gi|313882208|gb|AELG01000092.1| | 6674 | 15324 | 16 | 2 |
| XLOC\_016655 | gi|313882211|gb|AELG01000089.1| | 107587 | 139436 | 13 | 1 |
| XLOC\_016709 | gi|313882226|gb|AELG01000074.1| | 4007 | 13206 | 26 | 4 |
| XLOC\_016715 XLOC\_016723 | gi|313882226|gb|AELG01000074.1| | 95824 | 100838 | 15 | 1 |
| XLOC\_016718 | gi|313882226|gb|AELG01000074.1| | 14607 | 20246 | 11 | 1 |
| XLOC\_016724 XLOC\_016716 | gi|313882226|gb|AELG01000074.1| | 102007 | 117068 | 38 | 5 |
| XLOC\_016734 | gi|313882227|gb|AELG01000073.1| | 1028 | 18948 | 21 | 3 |
| XLOC\_016737 | gi|313882227|gb|AELG01000073.1| | 25751 | 32186 | 25 | 5 |
| XLOC\_016752 | gi|313882230|gb|AELG01000070.1| | 16238 | 22704 | 18 | 3 |
| XLOC\_016763 XLOC\_016764 | gi|313882241|gb|AELG01000059.1| | 29061 | 35453 | 6 | 1 |
| XLOC\_016766 | gi|313882242|gb|AELG01000058.1| | 2720 | 13287 | 11 | 1 |
| XLOC\_016768 XLOC\_016772 | gi|313882242|gb|AELG01000058.1| | 19070 | 29301 | 23 | 5 |
| XLOC\_016771 | gi|313882242|gb|AELG01000058.1| | 13485 | 18618 | 19 | 1 |
| XLOC\_016773 | gi|313882242|gb|AELG01000058.1| | 79435 | 191571 | 10 | 1 |
| XLOC\_016787 | gi|313882244|gb|AELG01000056.1| | 81126 | 91451 | 36 | 2 |
| XLOC\_016790 | gi|313882245|gb|AELG01000055.1| | 46907 | 51590 | 11 | 1 |
| XLOC\_016820 | gi|313882252|gb|AELG01000048.1| | 18364 | 41358 | 9 | 1 |
| XLOC\_016844 XLOC\_016846 | gi|313882267|gb|AELG01000033.1| | 3004 | 15662 | 21 | 1 |
| XLOC\_016845 | gi|313882267|gb|AELG01000033.1| | 16521 | 19492 | 14 | 1 |
| XLOC\_016860 | gi|313882272|gb|AELG01000028.1| | 17092 | 30162 | 9 | 2 |
| XLOC\_016861 | gi|313882272|gb|AELG01000028.1| | 31655 | 42262 | 13 | 2 |
| XLOC\_016868 | gi|313882280|gb|AELG01000020.1| | 43990 | 47461 | 2 | 2 |
| XLOC\_016869 | gi|313882280|gb|AELG01000020.1| | 48065 | 52898 | 7 | 1 |
| XLOC\_016876 | gi|313882280|gb|AELG01000020.1| | 53140 | 66397 | 39 | 2 |
| XLOC\_016878 | gi|313882280|gb|AELG01000020.1| | 69285 | 73010 | 15 | 1 |
| XLOC\_016886 | gi|313882283|gb|AELG01000017.1| | 50133 | 62018 | 17 | 1 |
| XLOC\_016890 | gi|313882283|gb|AELG01000017.1| | 43574 | 47837 | 8 | 1 |
| XLOC\_016894 | gi|313882284|gb|AELG01000016.1| | 42571 | 53058 | 5 | 3 |
| XLOC\_016895 | gi|313882284|gb|AELG01000016.1| | 64642 | 103580 | 6 | 2 |
| XLOC\_016896 XLOC\_016903 | gi|313882284|gb|AELG01000016.1| | 114873 | 144101 | 24 | 6 |
| XLOC\_016897 XLOC\_016904 XLOC\_016898 | gi|313882284|gb|AELG01000016.1| | 144161 | 169612 | 57 | 4 |
| XLOC\_016900 | gi|313882284|gb|AELG01000016.1| | 174948 | 208478 | 16 | 1 |
| XLOC\_016918 | gi|313882287|gb|AELG01000013.1| | 19937 | 27288 | 4 | 2 |
| XLOC\_016942 | gi|313882295|gb|AELG01000005.1| | 10255 | 24584 | 18 | 1 |
| XLOC\_016946 | gi|313882296|gb|AELG01000004.1| | 13983 | 27744 | 19 | 2 |
